# Supplementary material for: NMRProcFlow: a graphical and interactive tool dedicated to 1D spectra processing for NMR-based metabolomics
Source: Metabolomics. 2017 Feb 17;13(4):36. doi: 10.1007/s11306-017-1178-y (PMC5313591; doi:10.1007/s11306-017-1178-y)
Supplement: Supplementary file 1 — Supplementary material 1 (PDF 586 KB) [file 11306_2017_1178_MOESM1_ESM.pdf]

Supplemental Information

NMRProcFlow: A graphical and interactive tool dedicated to metabolomics for 1D NMR spectra processing

D. Jacob, C. Deborde, M. Lefebvre, M. Maucourt, A. Moing
UMR1332 Biologie du Fruit et Pathologie, INRA, Univ. Bordeaux,
Plateforme Métabolome Bordeaux-MetaboHUB,
71 avenue Edouard Bourlaux, 33140 Villenave d'Ornon, France
e-mail : daniel.jacob@inra.fr

Corresponding author: **Daniel Jacob,** daniel.jacob@inra.fr

S1 - Description of the implemented processing methods

Our criterion for the choice of methods for each spectra processing stage (baseline, alignment, binning ...) to implement in our application were those that:

1. were evaluated as effective according to our tests,
2. can be applied to both a small zone (<0.5 ppm) or a large zone (several ppm),
3. when applied on several dozen of spectra require only few seconds in their execution (implying an appropriate implementation) in order to allow a fluid use of the application.

When these methods were already implemented within a R package, then we integrated it. Otherwise, we implemented them ourselves in R / C ++.

Given the nature of the 1D NMR spectra and due to the diversity of problems encountered during the various stages of processing:

- *baseline correction, ppm calibration, removal of solvents and other contaminants, re-alignment of areas having high variations in chemical shifts between spectra, …*

and depending on:

- *the biological context (humans, plants, micro-organisms), the type of sample source (tissue or biofluid such as plasma, urine, plant extracts ...), the analytical protocol (choice of NMR sequence, use of additives for calibration and / or quantification, use of buffer solution to stabilize pH, etc ...),*

It is essential to process this type of data, with an interactive interface that enables spectra visualization. Being the heart of our approach, this aspect of interactive visualization is central to our method choices.

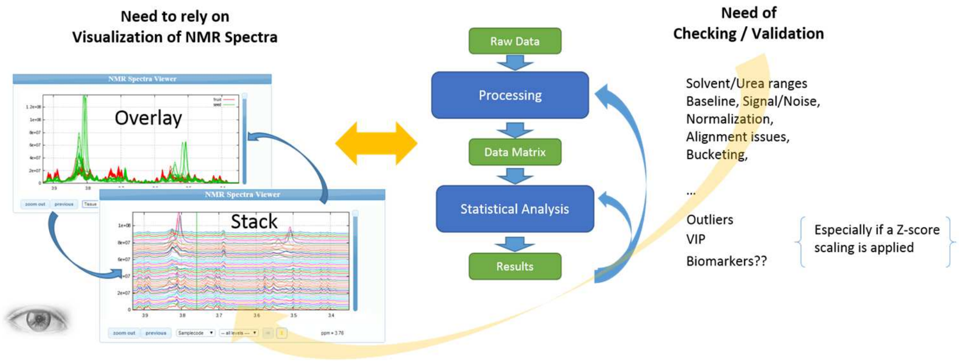

Thus NMRProcFlow provides a complete set of tools for processing 1D NMR data, the whole embedded within an interface allowing to interact with a spectra viewer.

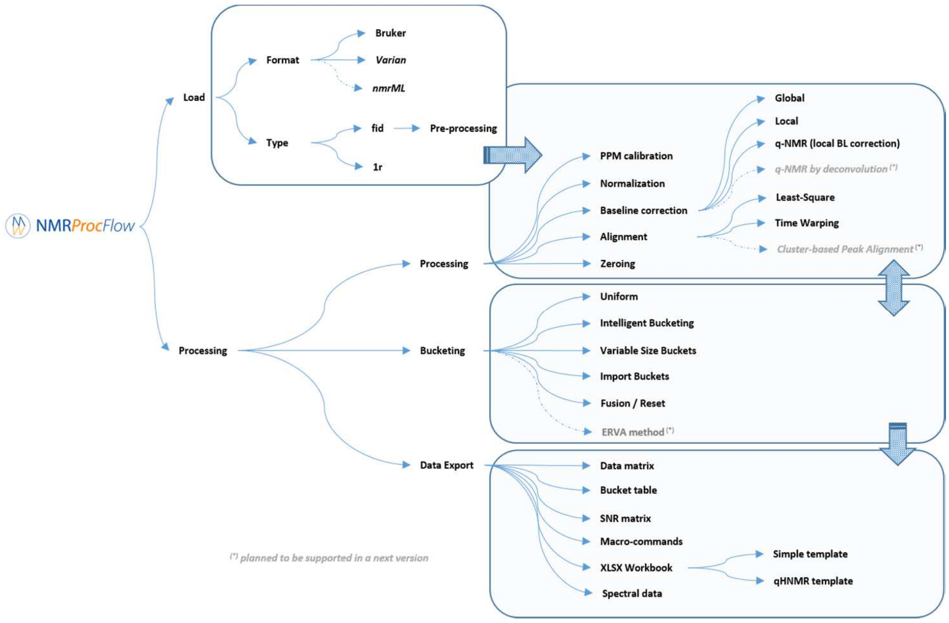

1 - Spectra pre-processing

(See Supplementary Information S2 p22)

The spectral preprocessing for 1D NMR can be automatically applied in case the input raw data are FID, either from a Bruker or Varian spectrometer. The term pre-processing designates here the transformation of the NMR spectrum from time domain to frequency domain, including the phase correction and the fast Fourier-transform (FFT).

From the work of Rousseau et al (2011), the adapted then implemented algorithm follows the steps (i.e. from FID to the corresponding real spectrum) depicted by the figure below:

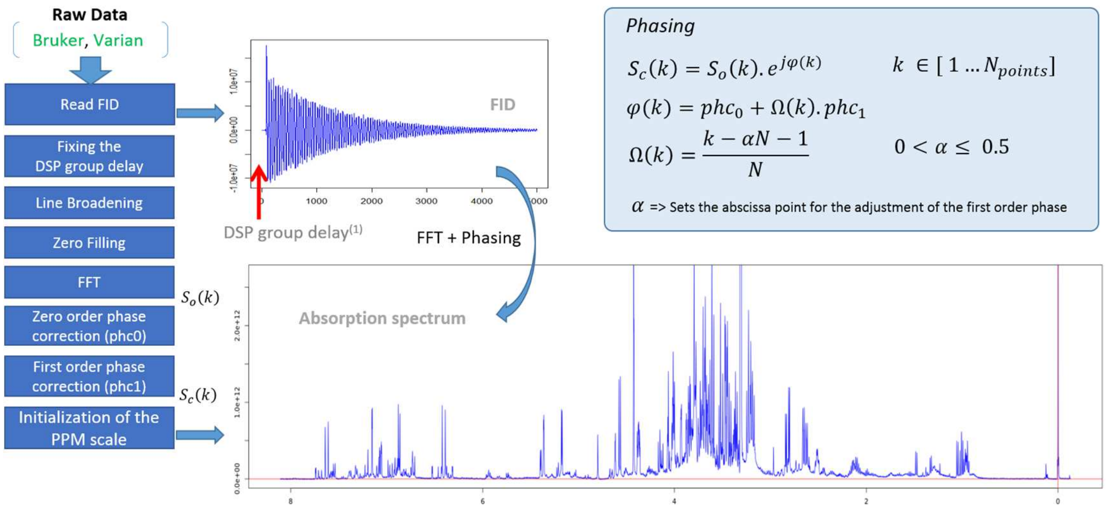

(1) In the case of Bruker spectra a death time or group delay can be observed in the FID.

Note: You can optimize the parameters to apply on your own raw spectra from the small application online at the URL http://www.bordeaux.inra.fr/pmb/spec/.

2 - Baseline correction

(See Supplementary Information S2 p 30)

Two types of Baseline correction were implemented: Global and Local. To be more efficient, both methods need to estimate the noise level, and by default the spectral or ppm range considered, is between 10.2 and 10.5 ppm. But users can choose another spectral range if some signal is present in this area.

2.1 - **Global baseline correction**

The global baseline correction was based on [Bao et al., 2012], but only two phases were implemented: i) Continuous Wavelet Transform (CWT), and ii) the sliding window algorithm. The user must choose the correction level, from 'soft' up to 'high'.

2.2 - **Local baseline correction**

The airPLS (adaptive iteratively reweighted penalized least squares) algorithm based on [Zhang et al., 2010] is a baseline correction algorithm that works completely on its own, and that only requires a “detail” parameter for the algorithm, called Lambda. Because this Lambda parameter can vary within a very large range (from 10 up to 1.e+06), we converted this parameter within a more convenient scale for the user, called 'level correction factor' chosen by the user from '1' (soft) up to '6' (high). The lower this level correction factor is set, the smoother baseline will be. Conversely, the higher this level correction factor is set, the more baseline will be corrected in details. To be more

efficient, the algorithm needs to estimate the noise level; by default the spectral or ppm range considered is between 10.2 and 10.5 ppm. However, users can choose another spectral range if some signal is present in this area.

2.3 - **q-HNMR baseline correction**

To quantify target compounds, a simple and efficient method is to fit the baseline–of a single resonance or a resonance pattern of the corresponding compounds and integrate these selected resonances. These resonance patterns or parts of them are chosen such that their peaks are not or little affected (i.e. in a smoothly way) by a possible overlap with a neighboring peak. In the figure below, the two targeted compounds (asparagine and aspartate) exhibit each a doublet of which only their baseline are affected by the trailing signal of a high intensity nearby lorentzian.

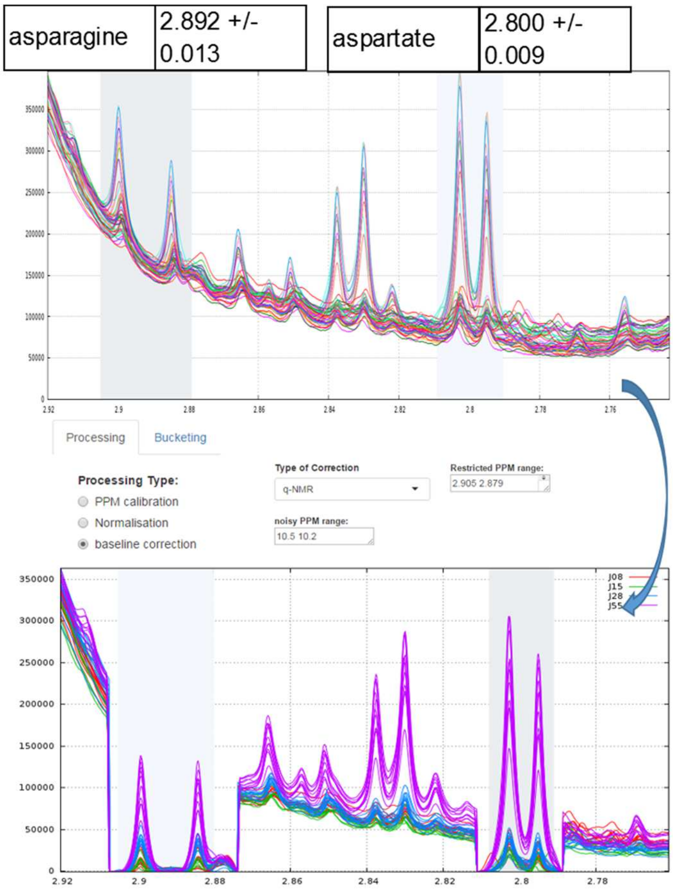

Note: This approach based on a local baseline correction applied on a specific pattern (singlet, doublet and triplet) works well when patterns are not overlapped (Moing et al. 2004). To address the issue in crowded regions with heavy peak overlap, a quantification method based on a local signal deconvolution seems more appropriate (Zheng et al. 2011). The implementation of such a method is planned in a future version of NMRprocFlow in order to cover these types of more complex needs but nevertheless widespread.

Which method to choose for baseline correction?

The spectra processing step consists in preserving as much as possible the variance between samples relative to the chemical compound signature ("useful" variance, i.e. informative) contained in the NMR spectra while reducing other types of variance induced by different sources of bias such as baseline ("unuseful" variance, i.e. non informative).

Nevertheless in the Metabolic Fingerprinting approach, we can accept a loss of "useful" variance, provided that each spectrum is equally affected (in %) by this loss (i.e. this does not affect the between-sample variances), if as a counterpart we can suppress all non-informative variances that unequally affect each spectrum (the variances being additional). The 'Local' baseline method is very efficient in that case, and this method can be applied to a large ppm range. In the figure below, the area ‘1’ is depending on the correction level.

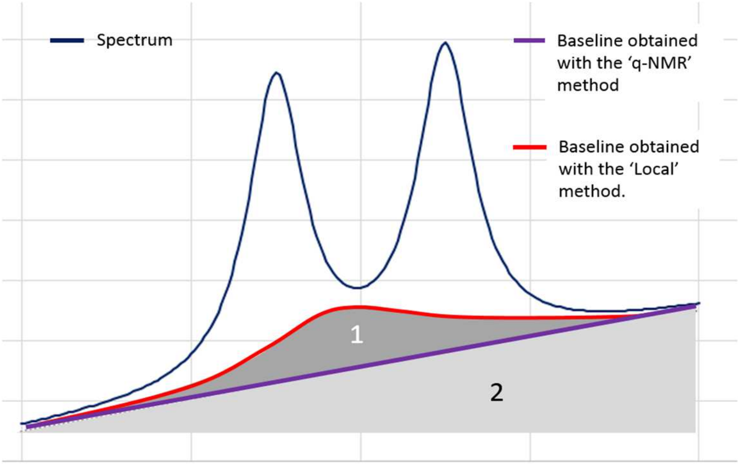

However, in the Targeted Metabolomics approach, a loss of "useful" variance even if it is slight cannot be tolerated; otherwise it also implies the loss of the 'absolute' feature of the quantification. In that case, the 'q-NMR' method is more relevant but it is to be applied to a small ppm range, i.e. a compound pattern (singlet, doublet or triplet).

3 - Spectra alignment

(See Supplementary Information S2 p 32)

The alignment step is undoubtedly one of the most tedious to solve. The misalignments are the results of changes in chemical shifts of NMR peaks largely due to differences in pH, ionic strength and other physicochemical interactions. To solve this prickly problem, we implemented two alignment methods, one based on a Least- Squares algorithm, and the other based on a Parametric Time Warping (PTW).

3.1 - **Least- Squares algorithm for alignment**

To align a set of spectra, we need to choose or to define a reference spectrum. The user can align spectra either based on a particular spectrum chosen within the spectra set, or based on the average spectrum. In this latter case, the re-alignment procedure is executed three times, the average spectrum being recalculated at each time.

In order to limit the relative ppm shift between the spectra to be realigned and the reference spectrum, the user can set this limit by adjusting the parameter 'Relative shift max.', that sets the maximum shift between spectra and the reference. The range goes from 0 (no ppm shift allowed) up to 1 (maximum ppm shift equal to 100% of the selected ppm range).

3.2 - **Parametric Time Warping for alignment**

The implementation is based on the R package 'ptw' (Bloemberg et al. 2010). In this method, the spectra alignment consists in approximating the ‘time’ (ppm in our case) axis of the reference signal by applying a polynomial transformation of the ‘time’ (i.e. ppm) axis of the sample to be aligned.

$$\hat{S}(t_k) = S(w(t_k))$$

where w() is the warping function. For more details, refer on the valuable explanations in Wehrens (2011).

Warnings: Wehrens R. (2011) highlights the fact that (§ 3.3.2) "alignment methods that are too flexible (such as PTW) may be led astray by the presence [so by the absence] of extra peaks, especially when these are of high intensity".

Note: In order to solve the different alignment cases, we have planned to include some other methods such as CluPA (Vu et al. 2013) or RUNAS (Alonso et al. 2014). We are currently testing these methods in order to evaluate their strengths and weaknesses compared to those we already implemented.

Which alignment method to choose?

Compliant with the NMRProcFlow philosophy and due to the diversity of problems encountered, for spectra alignment we chose the interactive approach. It means interval by interval, each interval being chosen by the user. We preconize to choose in priority the Least-Square method applied on spectra segments where intensities decrease to zero on both sides. It means that a baseline correction is also greatly preconized before attempting any alignment. Because the Least-Square method just applies a shift between spectra segments, this does not alter the lineshape of peaks.

Regarding Targeted Metabolomics, this point is crucial for an absolute quantification. Nevertheless, when in presence of highly overlapped peaks and/or a highly misaligned zone, it could be valuable to apply the PTW method even if some peaks are altered in order to have at least an estimation, either of the concentration (Targeted Metabolomics) or of the relative integration value (Metabolic Fingerprinting).

4 - Binning

(See Supplementary Information S2 p 36)

An NMR spectrum may contain several thousands of points, and therefore of variables. In order to reduce the data dimensionality binning or bucketing is commonly used. In binning, the spectra are divided into bins (so called buckets) and the total area (sum of each resonance intensity) within each bin is calculated to represent the original spectrum. The more simple approach (**Uniform bucketing**) consists in dividing all spectra with uniform spectral width (typically 0.01 to 0.04 ppm).

Due to the arbitrary division of spectrum, one bin may contain pieces from two or more resonances and one resonance may appear in two bins which may affect the data analysis. We have chosen to implement the Adaptive, Intelligent Binning method [De Meyer et al. 2008] that attempts to split the spectra so that each area common to all spectra contains the same single resonance, i.e. belonging to the same metabolite (**Intelligent bucketing**). In such methods, the width of each bucket is then determined by the maximum difference of chemical shifts among all spectra.

Another way for obtaining buckets is to choose the ppm ranges the user wants to integrate (**Variable size bucketing**). This method is typically used for the Targeted Metabolomics approach where only few resonances corresponding to targeted compounds are selected with their size depending on the signal pattern.

Which binning method to choose?

Regarding the Targeted Metabolomics approach, clearly the ‘Variable size bucketing’ has to be chosen for reasons given above. Regarding the Metabolic Fingerprinting approach, the choice is depending if the alignment has been done or not. If yes and even in a imperfect way, the Intelligent bucketing is clearly the most efficient. In case the alignment remains a too tedious task to be accomplished, one can choose the uniform bucketing as the most fast and simple (given that 'intelligence' will change nothing in the matter). But in this latter case, ***we strongly recommend after any subsequent statistical analyses to check the quality of the buckets especially those highlighted***

from Discriminant Analysis (e.g PLS-DA, OSC-PLS-DA ...). This has to be done with a critical eye by visualizing the overlaid spectra for these buckets, and thus ensuring that it is not due to a local misalignment.

5 - Buckets integration

The integration of buckets or zones of interest for targeted analysis is carried out according to the traditional trapezoid method. Knowing that a NMR spectrum is discretized at constant step, the formula is straightforward and described as follows.

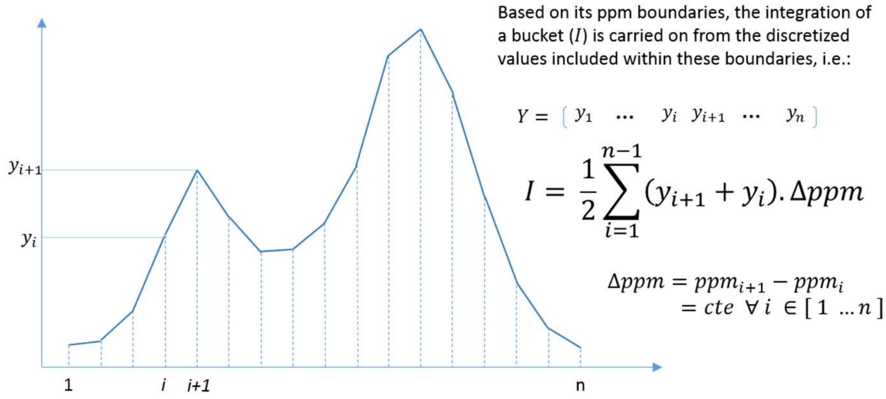

Based on its ppm boundaries, the integration of a bucket (I) is carried on from the discretized values included within these boundaries, i.e.:

$$Y = [\, y_1 \;\cdots\; y_i \;\; y_{i+1} \;\cdots\; y_n \,]$$

$$I = \frac{1}{2}\sum_{i=1}^{n-1}(y_{i+1} + y_i).\Delta ppm$$

$$\Delta ppm = ppm_{i+1} - ppm_i = cte \;\; \forall\, i \in [\, 1 \ldots n\,]$$

6 - Bucket normalization

(See Supplementary Information S2 p 40)

Before bucket data export, in order to make all spectra comparable with each other, the variations of the overall concentrations of samples have to be taken into account. We propose three normalization methods. In NMR metabolomics, the total intensity normalization (called the Constant Sum Normalization) is often used so that all spectra correspond to the same overall concentration. It simply consists in normalizing the total intensity of each individual spectrum to a same value.

Other methods such as Probabilistic Quotient Normalization [Dieterle et al. 2006] assume that biologically interesting concentration changes influence only parts of the NMR spectrum, while dilution effects will affect all metabolite signals. Probabilistic Quotient Normalization (PQN) starts by the calculation of a reference spectrum based on the median spectrum. Next, for each variable of interest the quotient of a given test spectrum and reference spectrum is calculated and the median of all quotients is estimated. Finally, all variables of the test spectrum are divided by the median quotient. An internal reference can be used to normalize the data. For example, an electronic reference (ERETIC, (see Akoka et al. 1999), or ERETIC2 generated with TopSpin software) can be used

for this purpose. The integral value of each bucket will be divided by the integral value of the ppm range given as reference.

Which normalization method to choose?

We suggest the reference [Kohl et al. 2012] as a good review that could be read with great profit.

References

Alonso, A., Rodríguez, M., Vinaixa, M., Tortosa, R., Correig, X., Julià, A., Marsal, S. (2014) Focus: a robust workflow for one-dimensional NMR spectral analysis. Anal Chem. 21;86(2):1160-9. doi: 10.1021/ac403110u

Akoka S1, Barantin L, Trierweiler M. (1999) Concentration Measurement by Proton NMR Using the ERETIC Method, Anal. Chem 71(13):2554-7. doi: 10.1021/ac981422i.

Bao, Q., Feng, J., Chen, F., Mao, W., Liu, Z., Liu, K., et al. (2012).A new automatic baseline correction method based on iterative method. Journal of Magnetic Resonance, 218, 35–43.

Bloemberg, T.G., Gerretzen, J., Wouters, H.J.P., Gloerich, J., van Dael, M., Wessels, H.J.C.T., et al. (2010). Improved parametric time warping for proteomics. Chemometrics and Intelligent Laboratory Systems, 104(1), 65-74.

De Meyer, T., Sinnaeve, D., Van Gasse, B., Tsiporkova, E., Rietzschel, E. R., De Buyzere, M. L., et al. (2008). MR-based characterization of metabolic alterations in hypertension using an adaptive, intelligent binning algorithm. Analytical Chemistry, 80(10), 3783–3790.

Dieterle F., Ross A., Schlotterbeck G. and Senn H. (2006). Probabilistic Quotient Normalization as Robust Method to Account for Dilution of Complex Biological Mixtures. Application in 1H NMR Metabonomics. Analytical Chemistry, 78:4281-4290.

Kohl SM, Klein MS, Hochrein J, Oefner PJ, Spang R, Gronwald W. (2012) State-of-the art data normalization methods improve NMR-based metabolomic analysis, Metabolomics 146-160, DOI:10.1007/s11306-011-0350-z

Moing, A., Maucourt, M., Renaud, C., Gaudillere, M., Brouquisse, R., Lebouteiller, B., Gousset-Dupont, A., Vidal, J., Granot, D., Denoyes-Rothan, B., Lerceteau-Kohler, E., Rolin, D. (2004) Quantitative metabolic profiling by 1-dimensional H-1-NMR analyses: application to plant genetics and functional genomics. Functional Plant Biology 31, 889-902.

Rousseau, R., Verleysen, M., Boulanger, B., von Sachs, R. et al (2011) Statistical_contribution_to_the_analysis_of_metabonomics_data_in_1H_NMR_spectroscopy, Thesis, https://www.researchgate.net/publication/233776575_Statistical_contribution_to_the_analysis_of_metabonomics_data_in_1H_NMR_spectroscopy (Last visit on January 2, 2017)

Vu T.N., Valkenborg D., Smets K., Verwaest K.A., Dommisse R., Lemière F., Verschoren A., Goethals B., Laukens K. An integrated workflow for robust alignment and simplified quantitative analysis of NMR spectrometry data. BMC Bioinformatics. 2011;12:405

Wehrens R. (2011) Chemometrics with R: Multivariate Data Analysis in the Natural Sciences and Life Sciences, Ed Springer-Verlag Berlin Heidelberg

Zhang Z, Chen S, and Liang Y-Z (2010) Baseline correction using adaptive iteratively reweighted penalized least squares, Analyst, 2010,135, 1138-1146. doi:10.1039/B922045C

Zheng, C., Zhang, S., Ragg, S., Raftery, D. and Vitek, O. (2011) Identification and quantification of metabolites in 1H NMR spectra by Bayesian model selection. Bioinformatics Vol. 27 (12) 1637:1644, doi:10.1093/bioinformatics/btr118.
