# Supplementary material for: NMRProcFlow: a graphical and interactive tool dedicated to 1D spectra processing for NMR-based metabolomics
Source: Metabolomics. 2017 Feb 17;13(4):36. doi: 10.1007/s11306-017-1178-y (PMC5313591; doi:10.1007/s11306-017-1178-y)
Supplement: Supplementary file 2 — Supplementary material 2 (PDF 7518 KB) [file 11306_2017_1178_MOESM2_ESM.pdf]

Supplemental Information
NMRProcFlow: A graphical and interactive tool dedicated to metabolomics for 1D NMR spectra processing

D. Jacob, C. Deborde, M. Lefebvre, M. Maucourt, A. Moing
UMR1332 Biologie du Fruit et Pathologie, INRA, Univ. Bordeaux,
Plateforme Métabolome Bordeaux-MetaboHUB,
71 avenue Edouard Bourlaux, 33140 Villenave d'Ornon, France
e-mail : daniel.jacob@inra.fr

Corresponding author: **Daniel Jacob,** daniel.jacob@inra.fr

S2 – NMRProcFlow User's documentation

(C) Copyright INRA UMR 1332 BFP - MetaboHub 2016

INRA SCIENCE & IMPACT

METABOHUB

NMRProcFlow

User's documentation

Contents

About NMRProcFlow 3

Overview 5

Metabolomics approaches 8

Metabolic Fingerprinting 10

Targeted Metabolomics 13

Data preparation phase 16

View the spectra 23

Interactive data processing 26

Spectra processing 29

Bucketing 36

Data Export 40

Restore a session 45

Batch mode execution 49

Examples in action 53

Download 56

Virtual Appliance 57

Docker Images 59

(C) Copyright INRA UMR 1332 BFP - MetaboHub 2016

About NMRProcFlow

© Copyright 2015-2017

- INRA UMR 1332 BFP, Bordeaux Metabolomics Facility
- France

Funded by:

- INRA UMR 1332 BFP, Bordeaux Metabolomics Facility
- the ANR-11-INBS-0010 grant (MetaboHUB)

Main contributors

- Daniel Jacob
- Catherine Deborde
- Marie Lefebvre
- Michaël Maucourt

Special thanks to Alain Girard (INRA Bordeaux) for designing the logo.

Call for contribution

We have been developing this software since 2015 and this requires a long and continuous effort. Firstly, our aim was to fulfill our own needs in the matter of NMR spectra processing, and we assume that it also meets your needs. Because no one can claim to have innate knowledge, we believe it is more beneficial for all to share our expertise. That's why we decided to give an open access of this software. So, this software is now a little yours too.

- An easy way to contribute is to keep trace of problems encountered with NMRProcFlow, and send them to us by email. Thus, it is a good way to ensure the development and the continual improvement of the NMRProcFlow system.
- Another way to contribute is to send us your suggestions about new functionalities that would be advisable to develop in priority, and those that must be improved.
- A third way to contribute is to propose your own R scripts or packages you would like to be integrated within the software in order to enrich the functionnalities.
- A last way to contribute is to become an NMRProcFlow developer (as soon as the code source will be opened).

Contact the maintainers: NMRProcFlow Team

(C) Copyright INRA UMR 1332 BFP - MetaboHub 2016

Training

NMRProcFlow has been developed to meet some expert needs. Although a non-expert user can use it, basic skills in NMR spectra processing is nevertheless required to take full advantage of its possibilities. Given that NMRProcFlow will do what you ask it to do, no matter if this is coherent or absurd, we think it is unfortunately not a software that can fill such a lack, but indeed an appropriate training (Weber et al 2015). This is why we are thinking about training courses for the year 2017 on the theme "Using NMRProcFlow to analyze 1H-NMR metabolomic data", covering data handling/processing applied to both targeted and untargeted approaches.

It should be noted that the online version of NMRProcFlow is not dedicated to intensive use and therefore is not suitable for a workshop with more than 10 simultaneous sessions. Also, we highly recommend the local installation of the application. For those who would like to organize training sessions or workshops where NMRProcFlow would be used as a support tool, please, contact the NMRProcFlow Team to see which strategy would be best suited.

Weber, Ralf J. M., Winder, Catherine L., Larcombe, Lee D., Dunn, Warwick B., Viant, Mark R. (2015) Training needs in metabolomics, Metabolomics 11:784-786. doi:10.1007/s11306-015-0815-6

Futur work

We have planned to develop some functionalities in order to meet particular needs

- Accept the new nmrML format as a valid input format for NMR spectra (FID and 1r) (ongoing work)
- Carry on the quantification (targeted metabolomics - qHNMR) based on signal deconvolution i.e. on line shape analysis of the signal (under study)
- Combine NMRProcFlow with the computing power of the workflow4metabolomics infrastructure.

License

- GNU GENERAL PUBLIC LICENSE Version 3, 29 June 2007 - See http://www.gnu.org/licenses/ for more details.

(C) Copyright INRA UMR 1332 BFP - MetaboHub 2016

Overview

An easy GUI tool dedicated to 1D NMR spectra processing (1H & 13C) for metabolomics

---

NMRProcFlow is an open source software that greatly helps spectra processing. It was built by involving NMR spectroscopists eager to have a quick and easy tool to use.

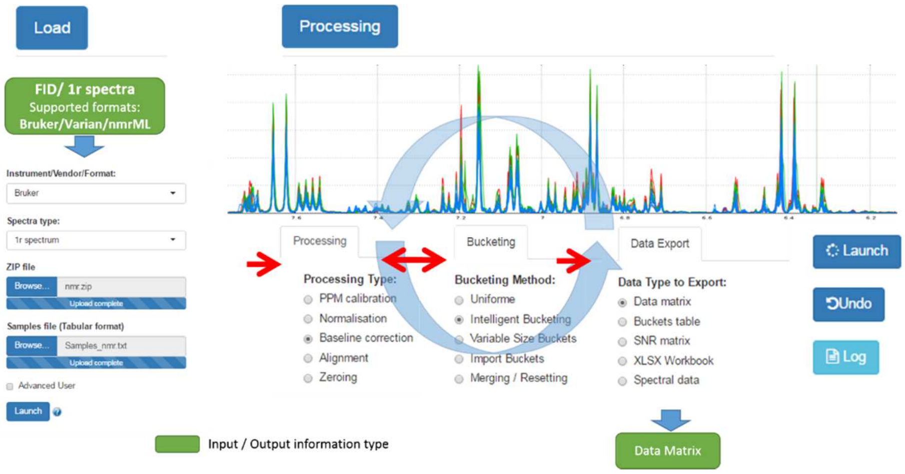

Given the nature of the 1D NMR spectra and due to the diversity of problems encountered during the various stages of processing:

- baseline correction,
- ppm calibration,
- removal of solvents and other contaminants
- re-alignment of areas having high variations in chemical shifts between spectra, ...

and depending on:

- the biological context (humans, plants, micro-organisms),
- the type of sample source (tissue or biofluid like plasma, urine, plant extracts ...),
- the analytical protocol (choice of NMR sequence, use of additives for calibration and / or quantification, use of buffer solution to stabilize pH, etc ...).

It is essential to process this type of data, with an interactive interface that enables spectra visualization.

(C) Copyright INRA UMR 1332 BFP - MetaboHub 2016

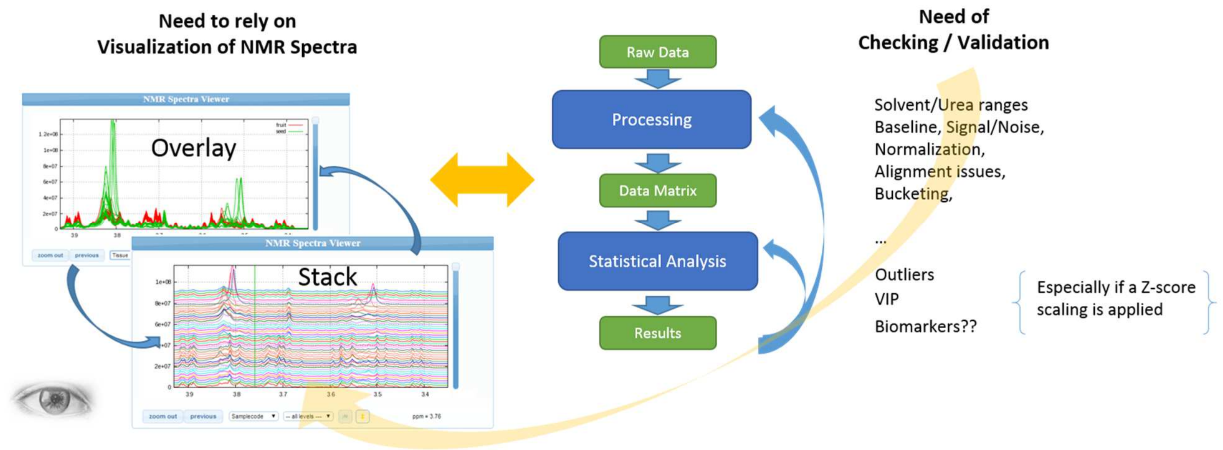

The expert's eyes are crucial to select the parameters, and to validate the treatments

> Apart for very well-mastered and very reproducible use cases (see Batch mode execution), the implementation of NMR spectra processing workflows executed in batch mode (regarding as a black-box) seems to us very hazardous, and can produce output aberrations. So, it is crucial to proceed in an interactive way with a NMR spectra viewer to allow the expert eye to disentangle the intertwined peaks.

Major concerns having (initially) motivated the design and having served as a roadmap:

- **Ease the data preparation phase in order to be loaded via the web interface**
- **View the spectra** according to the experimental conditions, or separately,
- Allow user to apply **interactive data processing procedures** to all the spectra, either to the whole ppm range with the same set of parameters or to only a selected ppm range with specific set of parameters for each ppm range,
- **Export a data matrix** to establish statistical analysis ((un)targeted approaches) with a statistical tool (such as BioStatFlow, MetaboAnalyst ...) **so that the file manipulations are minimized**.
- Allow user to **replay the same processing workflow** (e.g. few months later) on the same dataset or a similar one

NMRProcFlow open source software provides a complete set of tools for processing and visualizing of 1D NMR data, within an interactive interface based on a spectra visualization.

(C) Copyright INRA UMR 1332 BFP - MetaboHub 2016

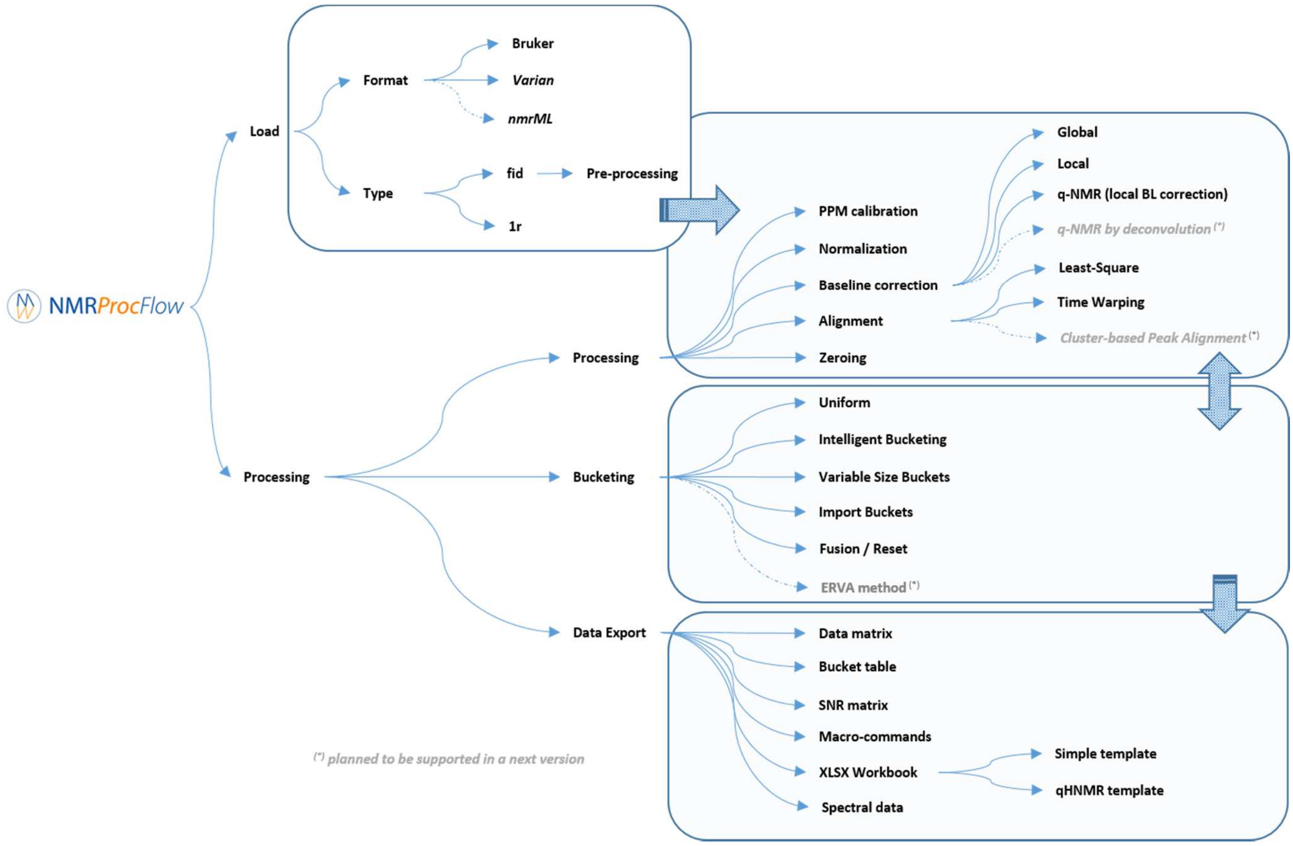

(C) Copyright INRA UMR 1332 BFP - MetaboHub 2016

Metabolomics approaches

NMRProcFlow is especially dedicated to metabolomics. The two major metabolomics approaches, namely metabolic fingerprinting and targeted metabolomics are taken into account. The workflow covers all steps from the spectral data up to the output data matrix

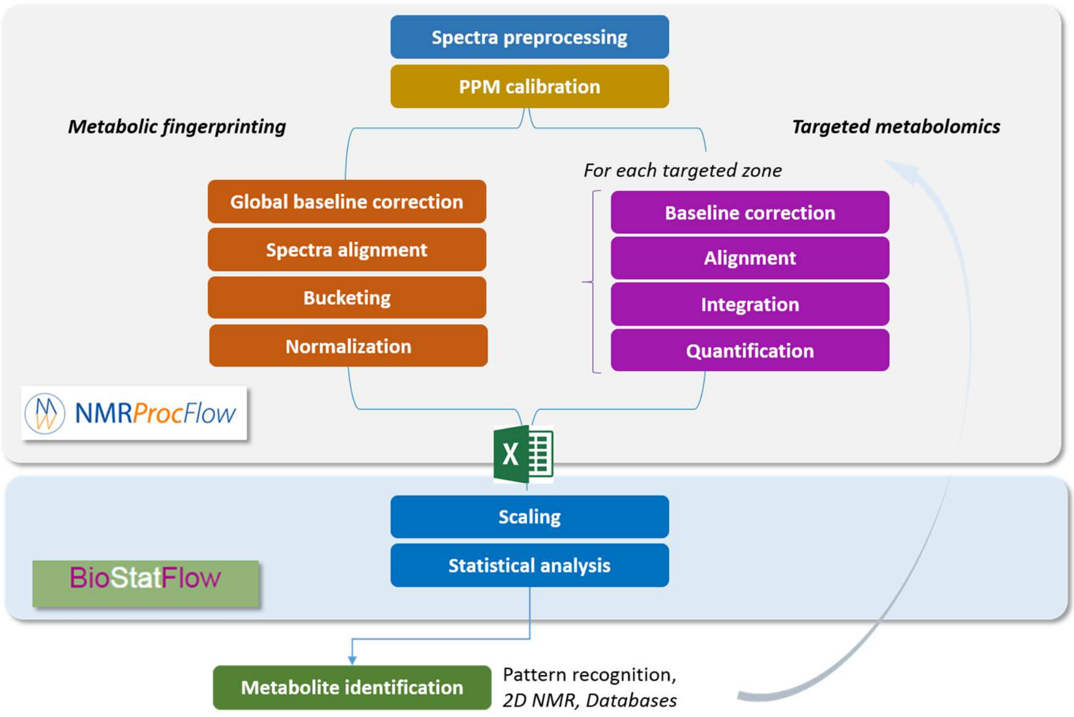

Metabolic Fingerprinting

The complex data are directly and initially used for global multivariate statistical analysis. Subsequently, **metabolite features that distinguish sample classes are identified** and then the structures of distinguishing metabolic features are established

Targeted Metabolomics

Quantitative approach wherein **a set of known metabolites are quantitated**. The identities of metabolites were initially established based on the available databases and using standard compounds. **The identified metabolite peaks are then quantified based on internal or external reference compounds**.

(C) Copyright INRA UMR 1332 BFP - MetaboHub 2016

Aims: Classification of samples & highlighting the metabolic biomarkers

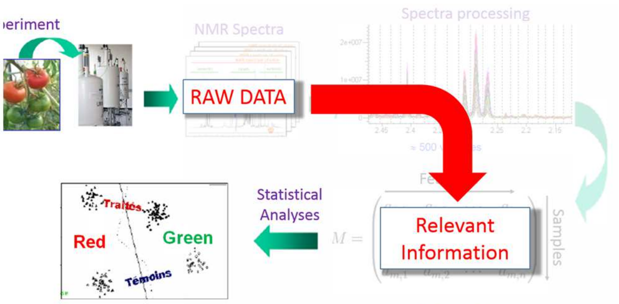

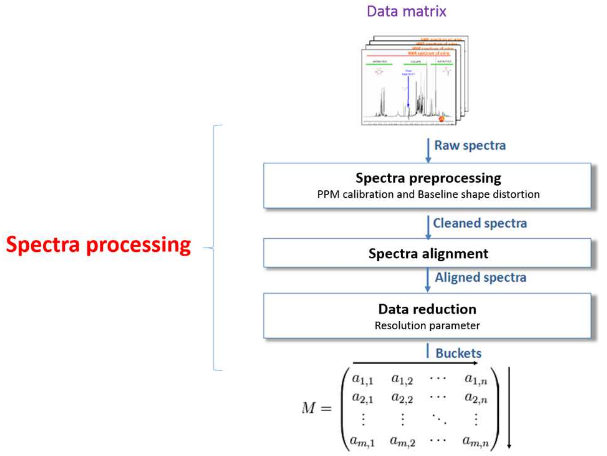

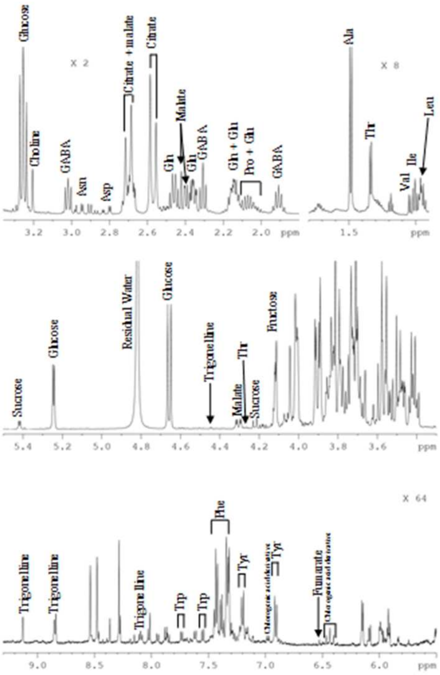

(C) Copyright INRA UMR 1332 BFP - MetaboHub 2016

Metabolic Fingerprinting

Metabolic fingerprinting refers to the use of machine output as potentially recognizable chemical pattern, specific of an individual sample. Metabolite fingerprinting by NMR is a fast, convenient, and effective tool for discriminating between groups of related samples and it identifies the most important regions of the spectra for further analysis.

So the spectra processing is an intermediate step between raw spectra and data analysis. It consists to preserve as much as possible the variance relative to the chemical compounds contained in the NMR spectra while reducing other types of variance induced by different sources of bias such as baseline and misalignment. See Spectra processing section.

Then, the identity of the metabolites of interest is established after statistical data analysis of metabolic fingerprints, and this involves to be able:

- to highlight that spectral regions having a difference between the groups are statistically significant.
- to ensure that each of these regions involves only a single metabolite, i.e. there is unique correspondence between a bucket and a resonance (spectral signature) of a metabolite

The standard approach in NMR-based metabolomics implies the division of spectra into equally sized bins, thereby simplifying subsequent data analysis. Yet, disadvantages are the loss of information and the occurrence of artifacts caused by peak shifts. Therefore we implemented the Adaptive Intelligent Binning (AI-Binning) algorithm which largely circumvents these problems. It recursively identifies bin edges in existing bins, requires only minimal user input, and avoids the use of arbitrary parameters or reference spectra. This algorithm is well adapted to meet the second point mentioned above.

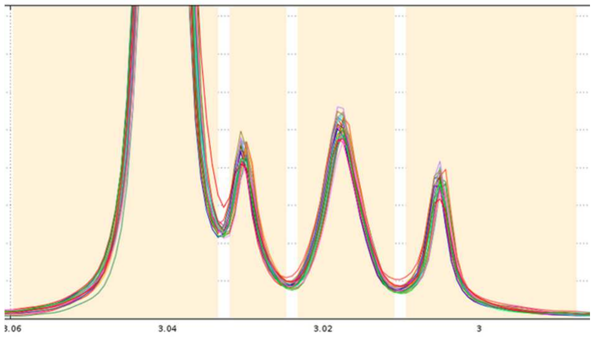

Intelligent Bucketing

De Meyer T., Sinnaeve D., Gasse B., Tsiporkova E., Rietzschel E., De Buyzere M., Gillebert T., Bekaert S., Martins J. and Criekinge W. (2008) NMR-Based Characterization of Metabolic Alterations in Hypertension Using an Adaptive, Intelligent Binning Algorithm. Analytical Chemistry 80(10):3783–3790

(C) Copyright INRA UMR 1332 BFP - MetaboHub 2016

How to further proceed?

After the bucketing, you have to export the data matrix (see Export the Data matrix). The exported matrix is formatted so that we can subsequently perform statistical analysis using BioStatFlow (*) web application. Thus the data file manipulations are minimized.

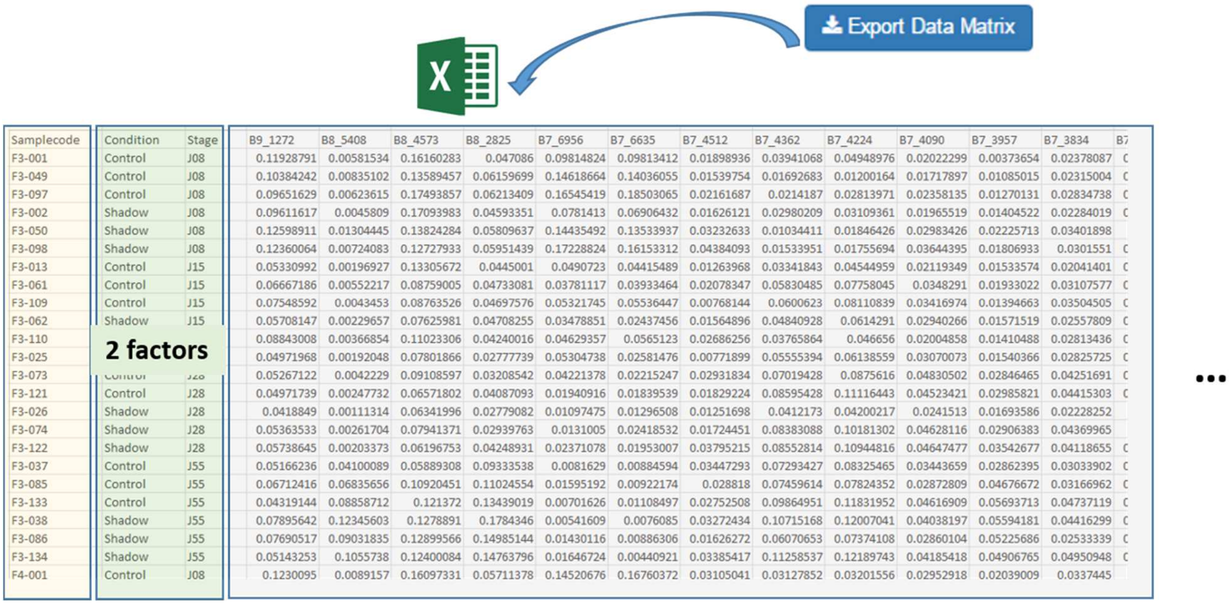

Samplecode	Condition	Stage	B9_1272	B8_5408	B8_4573	B8_2825	B7_6956	B7_6635
F3-001	Control	J08	0.11928791	0.00581534	0.16160283	0.047086	0.09814824	0.09813412
F3-049	Control	J08	0.10384242	0.00835102	0.13589457	0.06159699	0.14618664	0.14036055
F3-097	Control	J08	0.09651629	0.00623615	0.17493857	0.06213409	0.16545419	0.18503065
F3-002	Shadow	J08	0.09611617	0.0045809	0.17093983	0.04593351	0.0781413	0.06906432
F3-050	Shadow	J08	0.12598911	0.01304445	0.13824284	0.05809637	0.14435492	0.13533937
F3-098	Shadow	J08	0.12360064	0.00724083	0.12727933	0.05951439	0.17228824	0.16153312
F3-013	Control	J15	0.05330992	0.00196927	0.13305672	0.0445001	0.0490723	0.04415489
F3-061	Control	J15	0.06667186	0.00552217	0.08759005	0.04733081	0.03781117	0.03933464
F3-109	Control	J15	0.07548592	0.0043453	0.08763526	0.04697576	0.05321745	0.05536447
F3-062	Shadow	J15	0.05708147	0.00229657	0.07625981	0.04708255	0.03478851	0.02437456
F3-110	[illegible]	[illegible]	0.08843008	0.00366854	0.11023306	0.04240016	0.04629357	0.0565123
F3-025	[illegible]	[illegible]	0.04971968	0.00192048	0.07801866	0.02777739	0.05304738	0.02581476
F3-073	[illegible]	[illegible]	0.05267122	0.0042229	0.09108597	0.03208542	0.04221378	0.02215247
F3-121	Control	J28	0.04971739	0.00247732	0.06571802	0.04087093	0.01940916	0.01839539
F3-026	Shadow	J28	0.0418849	0.00111314	0.06341996	0.02779082	0.01097475	0.01296508
F3-074	Shadow	J28	0.05363533	0.00261704	0.07941371	0.02939763	0.0131005	0.02418532
F3-122	Shadow	J28	0.05738645	0.00203373	0.06196753	0.04248931	0.02371078	0.01953007
F3-037	Control	J55	0.05166236	0.04100089	0.05889308	0.09333538	0.0081629	0.00884594
F3-085	Control	J55	0.06712416	0.06835656	0.10920451	0.11024554	0.01595192	0.00922174
F3-133	Control	J55	0.04319144	0.08858712	0.121372	0.13439019	0.00701626	0.01108497
F3-038	Shadow	J55	0.07895642	0.12345603	0.1278891	0.1784346	0.00541609	0.0076085
F3-086	Shadow	J55	0.07690517	0.09031835	0.12899566	0.14985144	0.01430116	0.00886306
F3-134	Shadow	J55	0.05143253	0.1055738	0.12400084	0.14763796	0.01646724	0.00440921
F4-001	Control	J08	0.1230095	0.0089157	0.16097331	0.05711378	0.14520676	0.16760372

Samplecode	B7_4512	B7_4362	B7_4224	B7_4090	B7_3957	B7_3834
F3-001	0.01898936	0.03941068	0.04948976	0.02022299	0.00373654	0.02378087
F3-049	0.01539754	0.01692683	0.01200164	0.01717897	0.01085015	0.02315004
F3-097	0.02161687	0.0214187	0.02813971	0.02358135	0.01270131	0.02834738
F3-002	0.01626121	0.02980209	0.03109361	0.01965519	0.01404522	0.02284019
F3-050	0.03232633	0.01034411	0.01846426	0.02983426	0.02225713	0.03401898
F3-098	0.04384093	0.01533951	0.01755694	0.03644395	0.01806933	0.0301551
F3-013	0.01263968	0.03341843	0.04544959	0.02119349	0.01533574	0.02041401
F3-061	0.02078347	0.05830485	0.07758045	0.0348291	0.01933022	0.03107577
F3-109	0.00768144	0.0600623	0.08110839	0.03416974	0.01394663	0.03504505
F3-062	0.01564896	0.04840928	0.0614291	0.02940266	0.01571519	0.02557809
F3-110	0.02686256	0.03765864	0.046656	0.02004858	0.01410488	0.02813436
F3-025	0.00771899	0.05555394	0.06138559	0.03070073	0.01540366	0.02825725
F3-073	0.02931834	0.07019428	0.0875616	0.04830502	0.02846465	0.04251691
F3-121	0.01829224	0.08595428	0.11116443	0.04523421	0.02985821	0.04415303
F3-026	0.01251698	0.0412173	0.04200217	0.0241513	0.01693586	0.02228252
F3-074	0.01724451	0.08383088	0.10181302	0.04628116	0.02906383	0.04369965
F3-122	0.03795215	0.08552814	0.10944816	0.04647477	0.03542677	0.04118655
F3-037	0.03447293	0.07293427	0.08325465	0.03443659	0.02862395	0.03033902
F3-085	0.028818	0.07459614	0.07824352	0.02872809	0.04676672	0.03166962
F3-133	0.02752508	0.09864951	0.11831952	0.04616909	0.05693713	0.04737119
F3-038	0.03272434	0.10715168	0.12007041	0.04038197	0.05594181	0.04416299
F3-086	0.01626272	0.06070653	0.07374108	0.02860104	0.05225686	0.02533339
F3-134	0.03385417	0.11258537	0.12189743	0.04185418	0.04906765	0.04950948
F4-001	0.03105041	0.03127852	0.03201556	0.02952918	0.02039009	0.0337445

Note that the factors are embedded in the file, provided they have been specified in the first step (file samples)

Import the Data Matrix to BioStatFlow

After exporting, the data matrix is formatted so that we can subsequently perform statistical analysis using BioStatFlow(*) web application. Thus the data file manipulations are minimized.

(C) Copyright INRA UMR 1332 BFP - MetaboHub 2016

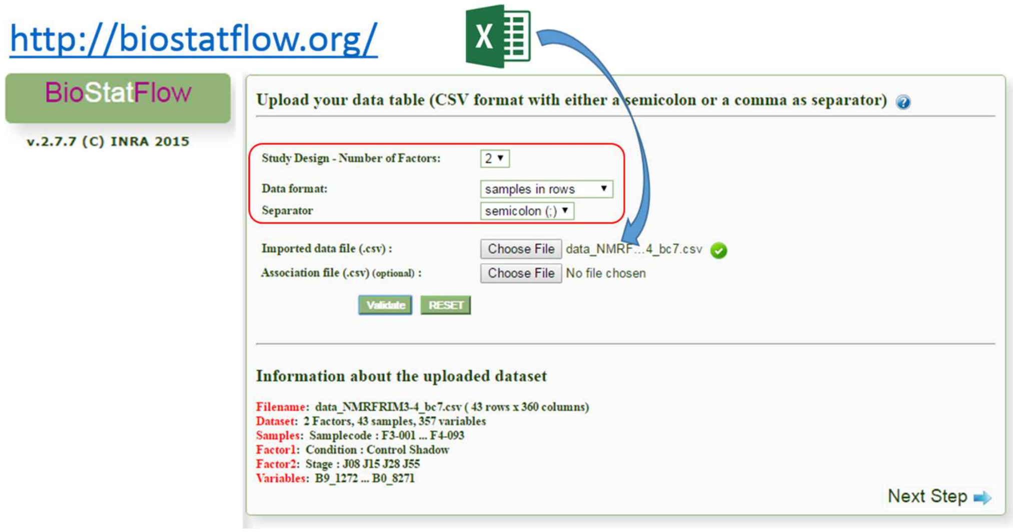

(*) See MetaboNews Issue 42 - February 2015

See online some slides showing a simple session of BioStatFlow in action: Example of a BioStatFlow session (http://nmrprocflow.org/themes/pdf/BioStatFlow.pdf).

Import the Data Matrix to MetaboAnalyst

After exporting, the data matrix is formatted so that we can subsequently perform statistical analysis using MetaboAnalyst (Xia et al. 2015). See online some slides showing a simple session with MetaboAnalyst (http://nmrprocflow.org/themes/pdf/MetaboAnalyst.pdf)

Xia, J., Sinelnikov, I., Han, B., and Wishart, D.S. (2015) MetaboAnalyst 3.0 - making metabolomics more meaningful . Nucl. Acids Res. 43, W251-257.

Help in the identification

Still in the embryonic stage, we currently develop tools that will greatly help in the identification. See online some slides showing about Help in the identification (http://nmrprocflow.org/themes/pdf/Help_in_the_Identification.pdf)

(C) Copyright INRA UMR 1332 BFP - MetaboHub 2016

Targeted Metabolomics

This refers to quantitative approaches wherein a set of known metabolites are quantitated. The identities of metabolites have been initially established based on the available databases and using standard compounds. The identified metabolite peaks are then quantified based on internal or external reference compounds. Here, we suggest two references as good reviews that could be read with great profit:

Santosh Kumar Bharti, Raja Roy (2012) Quantitative 1H NMR, spectroscopy, Trends in Analytical Chemistry 35:5-26, doi:10.1016/j.trac.2012.02.007

Patrick Giraudeau, Illa Tea, Gérald S. Remaud, Serge Akoka (2014) Reference and normalization methods: Essential tools for the intercomparison of NMR spectra, Journal of Pharmaceutical and Biomedical Analysis 93:3–16, doi:10.1016/j.trac.2012.02.007

In this case the identity of the metabolites of interest is established before statistical data analysis, and this involves to be able:

- to identify the ppm areas for which the quantification will be performed based on both knowledge and well-established metabolomic profiles,
- to ensure that each of these areas is not polluted by the neighbor areas

To fulfill these two points, it is necessary to locally correct the baseline in order to i) eliminate the residual effects due to the presence of macromolecules in extracts, ii) but also reduce the prevalence of a high intense peak on the less intense ones.

Typical approach for quantifying compounds via 1H NMR

A typical approach for quantifying compounds via 1H NMR is based on Calibration-curve method (i.e. external reference optionaly coupled with an internal reference such as ERETIC). For more explanation and details, see the reference given above.

The figure below shows a ppm area where are located 3 amino acids (valine, leucine and isoleucine), (**A**) before any local baseline correction, and (**B**) after local baseline correction ('q-NMR' type) in NMRProcFlow, (**C**) The 1H NMR reference patterns for each of these amino acids are shown for this ppm area. The colored areas (one color per compound) correspond to the areas from which the quantification will be done. The peak highlighted by the arrow is left aside because it is the sum of 2 amino acids, namely Leu+Ile. In order to check the quality of the baseline correction, the graphs below show the correlation between 2 buckets belonging to the same compound, before and after the baseline correction.

(C) Copyright INRA UMR 1332 BFP - MetaboHub 2016

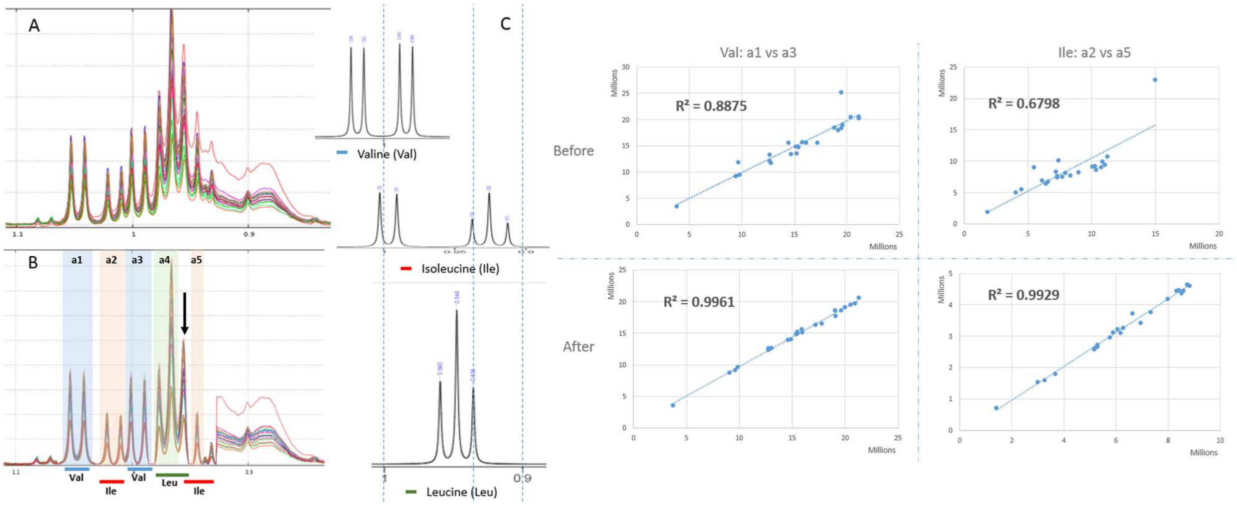

After the processing and bucketing steps, NMRProcFlow allows users to export all data needed for the quantification in a same XLSX workbook. Two workbook templates were currently available: A simple one and a template dedicated for the quantification.

The simple template just aggregates the buckets table, the SNR matrix and the data matrix, each data type being within a separate tab.

The 'qHNMR' template, in the same way as the simple template aggregates information like the samples table, the buckets table, the SNR matrix and the data matrix within separate tabs, but also includes another tab with the pre-calculated quantifications according to a formula from data provided in the others tabs. Some information are set by default in both 'samples' and 'buckets' tabs. Just adjust them with the appropriate values and the quantifications within the eponymous tab will be automatically updated as depicted in the figure below:

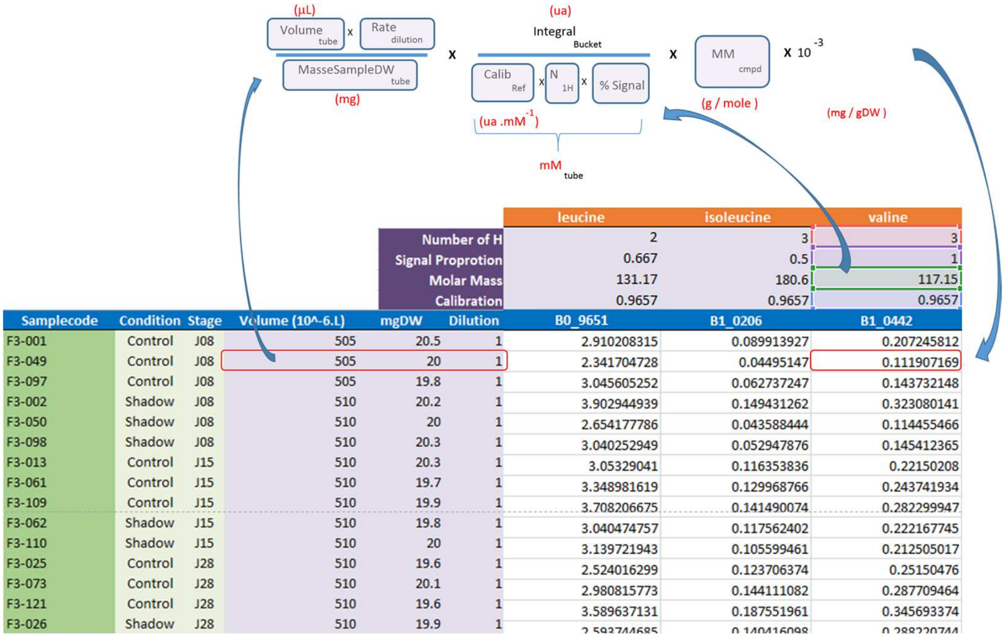

						leucine	isoleucine	valine
					Number of H	2	3	3
					Signal Proprotion	0.667	0.5	1
					Molar Mass	131.17	180.6	117.15
					Calibration	0.9657	0.9657	0.9657
Samplecode	Condition	Stage	Volume (10^-6.L)	mgDW	Dilution	B0_9651	B1_0206	B1_0442
F3-001	Control	J08	505	20.5	1	2.910208315	0.089913927	0.207245812
F3-049	Control	J08	505	20	1	2.341704728	0.04495147	0.111907169
F3-097	Control	J08	505	19.8	1	3.045605252	0.062737247	0.143732148
F3-002	Shadow	J08	510	20.2	1	3.902944939	0.149431262	0.323080141
F3-050	Shadow	J08	510	20	1	2.654177786	0.043588444	0.114455466
F3-098	Shadow	J08	510	20.3	1	3.040252949	0.052947876	0.145412365
F3-013	Control	J15	510	20.3	1	3.05329041	0.116353836	0.22150208
F3-061	Control	J15	510	19.7	1	3.348981619	0.129968766	0.243741934
F3-109	Control	J15	510	19.9	1	3.708206675	0.141490074	0.282299947
F3-062	Shadow	J15	510	19.8	1	3.040474757	0.117562402	0.222167745
F3-110	Shadow	J15	510	20	1	3.139721943	0.105599461	0.212505017
F3-025	Control	J28	510	19.6	1	2.524016299	0.123706374	0.25150476
F3-073	Control	J28	510	20.1	1	2.980815773	0.144111082	0.287709464
F3-121	Control	J28	510	19.6	1	3.589637131	0.187551961	0.345693374
F3-026	Shadow	J28	510	19.9	1	[illegible]	[illegible]	[illegible]

(C) Copyright INRA UMR 1332 BFP - MetaboHub 2016

See online some slides about the targeted approach along with NMRProcFlow: Targeted metabolomics (http://nmrprocflow.org/themes/pdf/Targeted.pdf)

(C) Copyright INRA UMR 1332 BFP - MetaboHub 2016

Data preparation phase

The current version of NMRProcFlow accepts raw data come from two major vendors namely Bruker GmbH & Agilent Technologies (Varian). We have planned to support the nmrML format in a next version (See Futur work section)

Bruker

Regarding Bruker, two types of raw data are accepted: Free Induction Decay (fid) and pre-processed raw spectra (1r). In both cases, the folder structure must follow that of the Bruker TopSpin software. "Pre-processed" means that it assumes that Fourier transform and phase correction have been applied on all spectra so that their corresponding processing directory (under 'pdata') exists along with their real spectrum (i.e 1r file). In the case where the input raw data are FID, the spectral pre-processing is automatically performed. See the Spectral pre-processing for 1D NMR section.

To ease the preparation phase, **simply zip the entire directory including all spectra of the experiment**. This means that it is useless to perform any prior selection or having to rename the numbers of experiment and processing. The figure below shows an example of ZIP file **along with its corresponding samples file.**

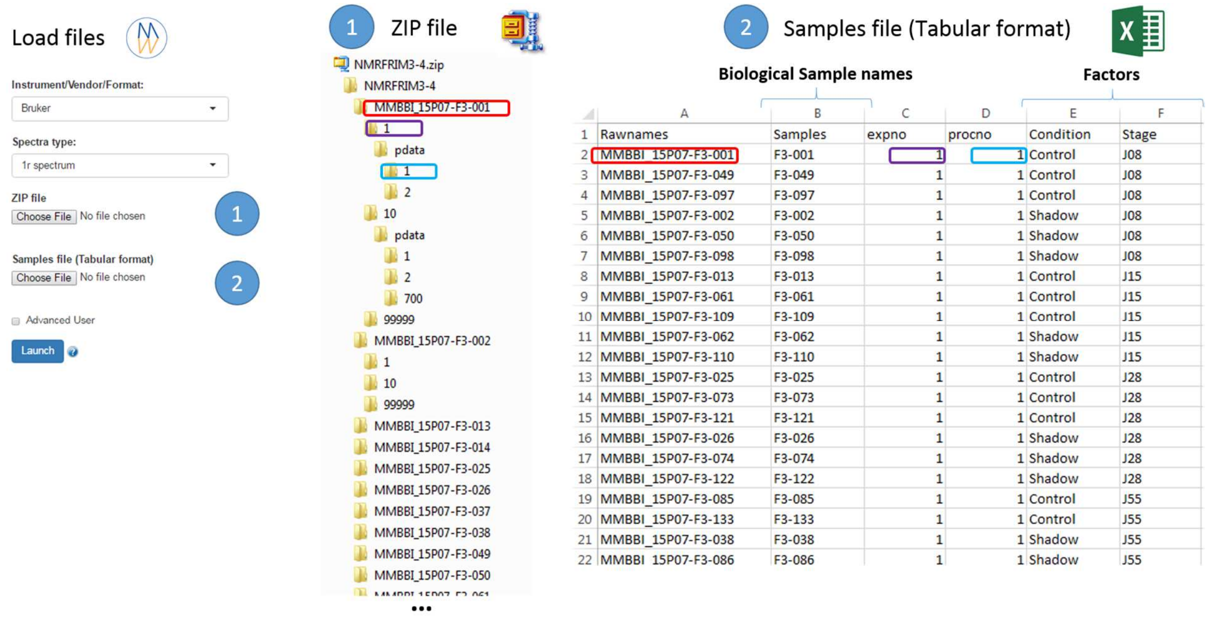

Information provided within the samples file must correspond to the directories contained in the ZIP file.

The colored boxes show the correspondences.

(C) Copyright INRA UMR 1332 BFP - MetaboHub 2016

Supported directory structures for Bruker NMR spectra

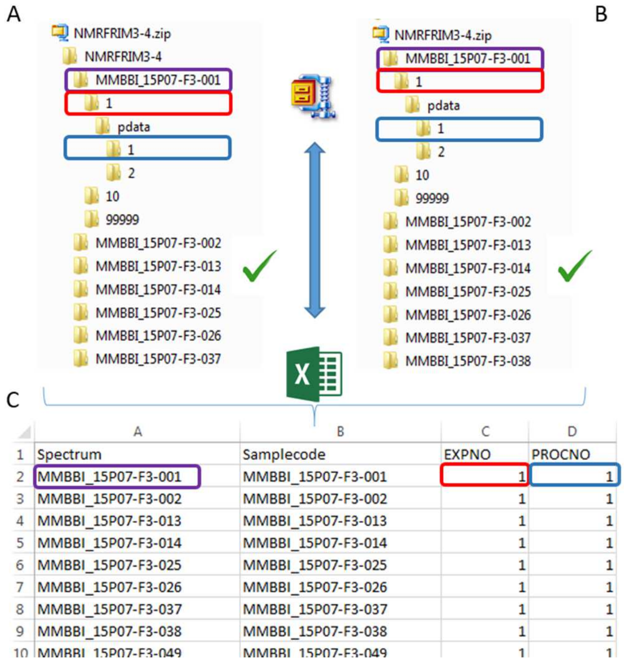

	A	B	C	D
1	Spectrum	Samplecode	EXPNO	PROCNO
2	MMBBI_15P07-F3-001	MMBBI_15P07-F3-001	1	1
3	MMBBI_15P07-F3-002	MMBBI_15P07-F3-002	1	1
4	MMBBI_15P07-F3-013	MMBBI_15P07-F3-013	1	1
5	MMBBI_15P07-F3-014	MMBBI_15P07-F3-014	1	1
6	MMBBI_15P07-F3-025	MMBBI_15P07-F3-025	1	1
7	MMBBI_15P07-F3-026	MMBBI_15P07-F3-026	1	1
8	MMBBI_15P07-F3-037	MMBBI_15P07-F3-037	1	1
9	MMBBI_15P07-F3-038	MMBBI_15P07-F3-038	1	1
10	MMBBI_15P07-F3-049	MMBBI_15P07-F3-049	1	1

*(**A**): Each sample has its own directory (e.g MMBBI_15P07-F3-001) containing the different acquisition spectra (1, 10, 99999), the whole being contained under a root directory (i.e. NMRFRIM3-4). (**B**): Same as (**A**), but without the root directory. (**C**) is the sample file corresponding to both directory structures.*

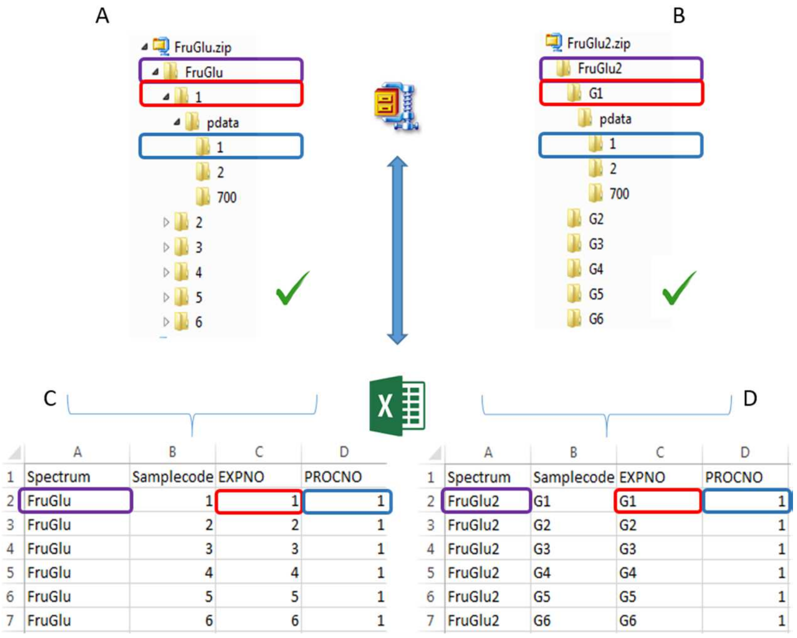

	A	B	C	D
1	Spectrum	Samplecode	EXPNO	PROCNO
2	FruGlu	1	1	1
3	FruGlu	2	2	1
4	FruGlu	3	3	1
5	FruGlu	4	4	1
6	FruGlu	5	5	1
7	FruGlu	6	6	1

	A	B	C	D
1	Spectrum	Samplecode	EXPNO	PROCNO
2	FruGlu2	G1	G1	1
3	FruGlu2	G2	G2	1
4	FruGlu2	G3	G3	1
5	FruGlu2	G4	G4	1
6	FruGlu2	G5	G5	1
7	FruGlu2	G6	G6	1

A root directory contains all acquisition spectra (one for each sample).

*Acquisition spectra names can be a number (**A**), or a string (**B**). (**C**) and (**D**) are the sample files corresponding to the directory structures. Both directory structures shown in (**E**) are not supported.*

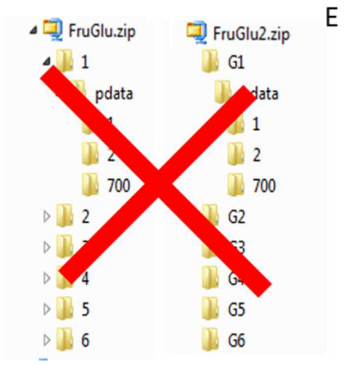

(C) Copyright INRA UMR 1332 BFP - MetaboHub 2016

Information provided within the samples file must correspond to the directories contained in the ZIP file. (*The colored boxes show the correspondences*)

- The '**rawdata**' column may include all directories or just a subset contained in the ZIP file.
- The '**Samplecode**' column can be filled with the biological sample name or can be just a copy-paste of the 'Rawdata' column.
- The '**expno**' and '**procno**' columns correspond to the experiment number (i.e. FID) and the processing number (i.e. 1r) respectively.
- Several **factor** columns can be added which will allow spectra to be visualized according to theirs factor levels.

In this way, it becomes **easy to select each NMR spectrum that we want to include** into the spectra serial in order to be processed together. In the absence of the file of samples provided as an input, NMRProcFlow will consider all of the root directories in the zip file by default, looking for the smallest FID identifier (expno) and the smallest processing identifier (procno) for each of them.

To facilitate the generation of the samples file, it is possible to proceed as follows:

- Upload the ZIP file only, and then NMRProcFlow will produce a text file containing the acquisition and processing parameters for each spectrum taken into account by default,
- Download the parameters file ('Export Parameters'),
- Edit this file to serve as a starting template for generating the samples file.

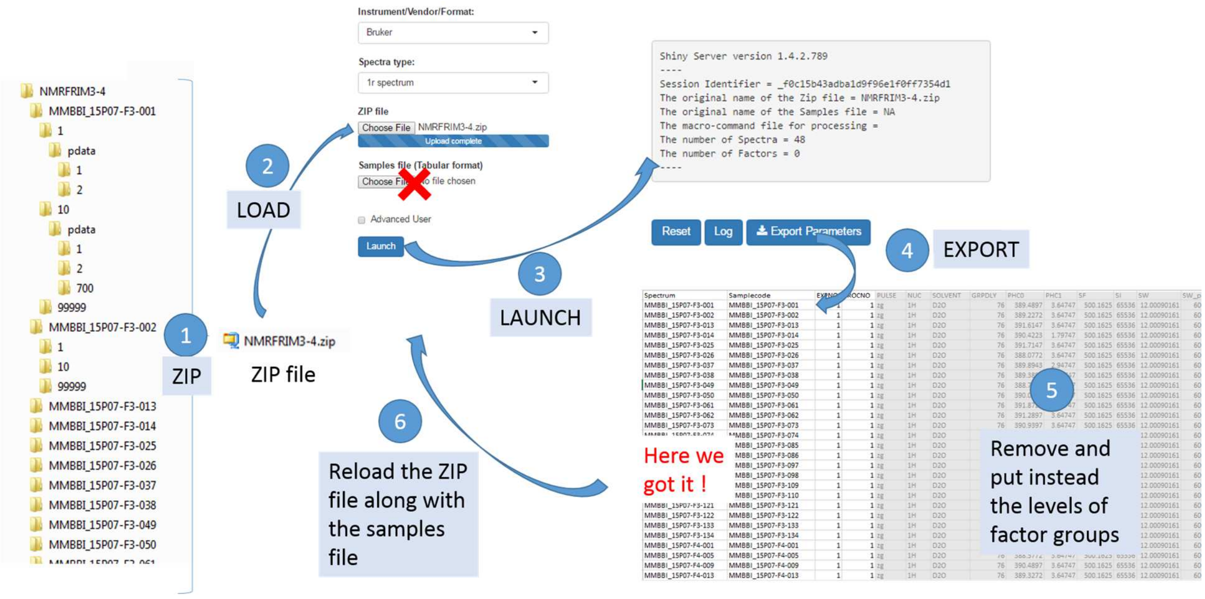

Once uploaded files, you can click on 'Launch' to start the pretreatment.

Once completed, the list of spectra considered with their acquisition and pre-processing parameters is provided

(C) Copyright INRA UMR 1332 BFP - MetaboHub 2016

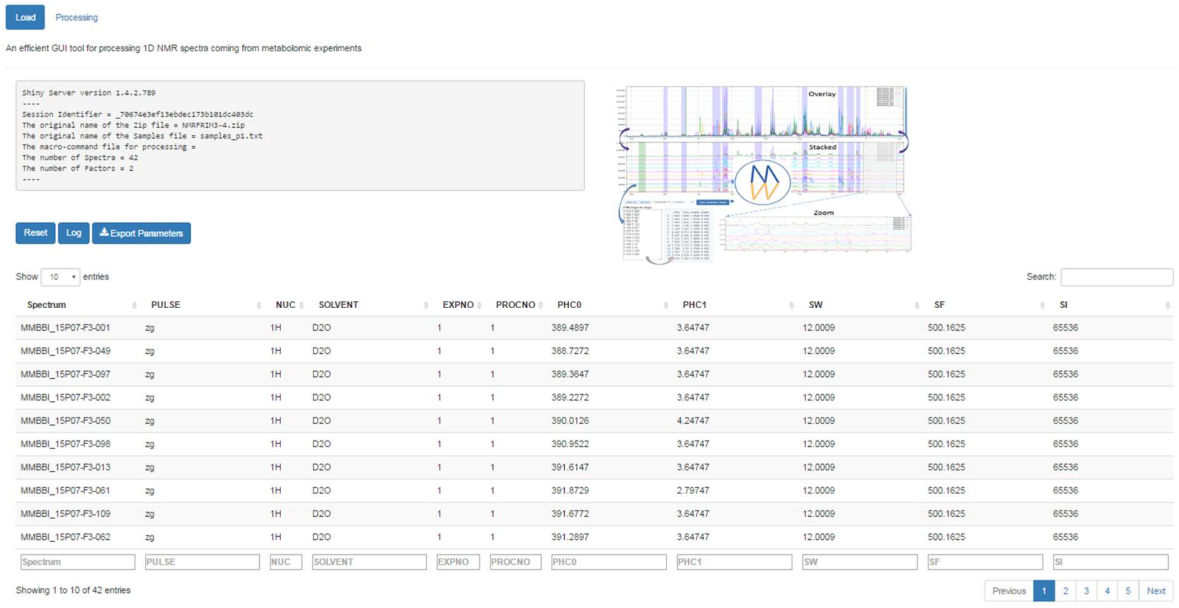

Spectrum	PULSE	NUC	SOLVENT	EXPNO	PROCNO	PHC0	PHC1	SW	SF	SI
MMBBI_15P07-F3-001	zg	1H	D2O	1	1	389.4897	3.64747	12.0009	500.1625	65536
MMBBI_15P07-F3-049	zg	1H	D2O	1	1	388.7272	3.64747	12.0009	500.1625	65536
MMBBI_15P07-F3-097	zg	1H	D2O	1	1	389.3647	3.64747	12.0009	500.1625	65536
MMBBI_15P07-F3-002	zg	1H	D2O	1	1	389.2272	3.64747	12.0009	500.1625	65536
MMBBI_15P07-F3-050	zg	1H	D2O	1	1	390.0126	4.24747	12.0009	500.1625	65536
MMBBI_15P07-F3-098	zg	1H	D2O	1	1	390.9522	3.64747	12.0009	500.1625	65536
MMBBI_15P07-F3-013	zg	1H	D2O	1	1	391.6147	3.64747	12.0009	500.1625	65536
MMBBI_15P07-F3-061	zg	1H	D2O	1	1	391.8729	2.79747	12.0009	500.1625	65536
MMBBI_15P07-F3-109	zg	1H	D2O	1	1	391.6772	3.64747	12.0009	500.1625	65536
MMBBI_15P07-F3-062	zg	1H	D2O	1	1	391.2897	3.64747	12.0009	500.1625	65536
Spectrum	PULSE	NUC	SOLVENT	EXPNO	PROCNO	PHC0	PHC1	SW	SF	SI

To switch to the processing steps, you must click on the '**Processing' tab** at the top of the screen.

Varian/Agilent

Regarding Agigent/Varian, only the Free Induction Delay are accepted, given that there is no normalized folder structure for pre-processed raw data provided by the VnmrJ software. See the Spectral pre-processing for 1D NMR section to know how to choose parameters.

To ease the preparation phase, **simply zip the entire directory including all spectra of the experiment**. This means that it is useless to perform any prior selection or having to rename the numbers of experiment and processing. The figure below shows an example of ZIP file **along with its corresponding samples file.**

(C) Copyright INRA UMR 1332 BFP - MetaboHub 2016

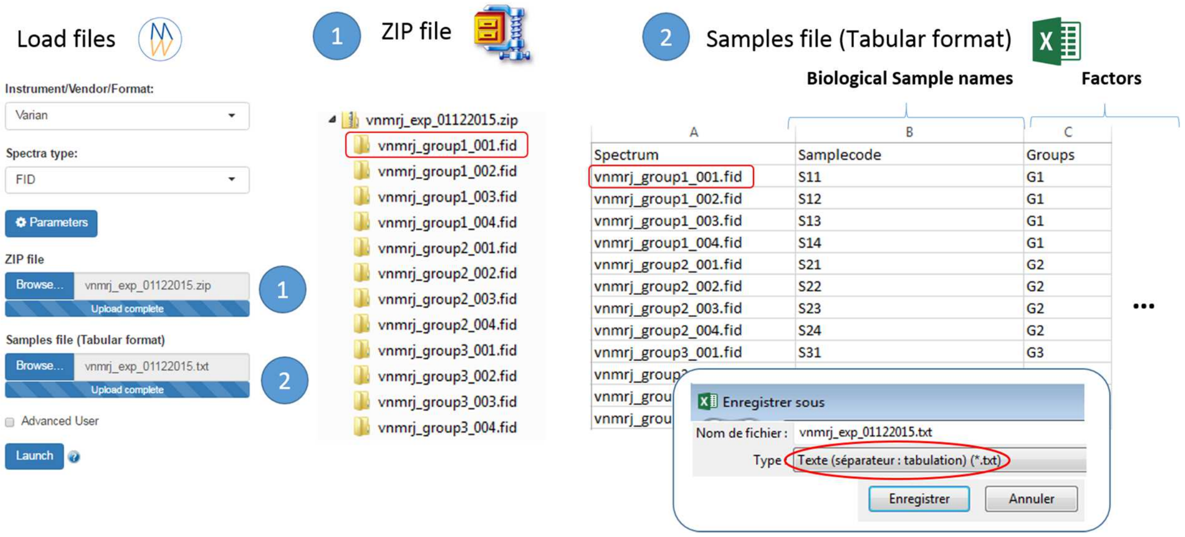

Information provided within the samples file must correspond to the directories contained in the ZIP file. (*The colored boxes show the correspondences*)

- The '**rawdata**' column may include all directories or just a subset contained in the ZIP file.
- The '**Samplecode**' column can be filled with the biological sample name or can be just a copy-paste of the 'Rawdata' column.
- Several **factor** columns can be added which will allow spectra to be visualized according to theirs factor levels.

In this way, it becomes **easy to select each NMR spectrum that we want to include** into the spectra serial in order to be processed together. In the absence of the file of samples provided as an input, NMRProcFlow will consider all of the root directories in the zip file by default, looking for all FID files.

To facilitate the generation of the samples file, see the corresponding section in the 'Bruker' tab

Once uploaded files, you can click on 'Launch' to start the pretreatment.

Once completed, the list of spectra considered with their acquisition and pre-processing parameters is provided

(C) Copyright INRA UMR 1332 BFP - MetaboHub 2016

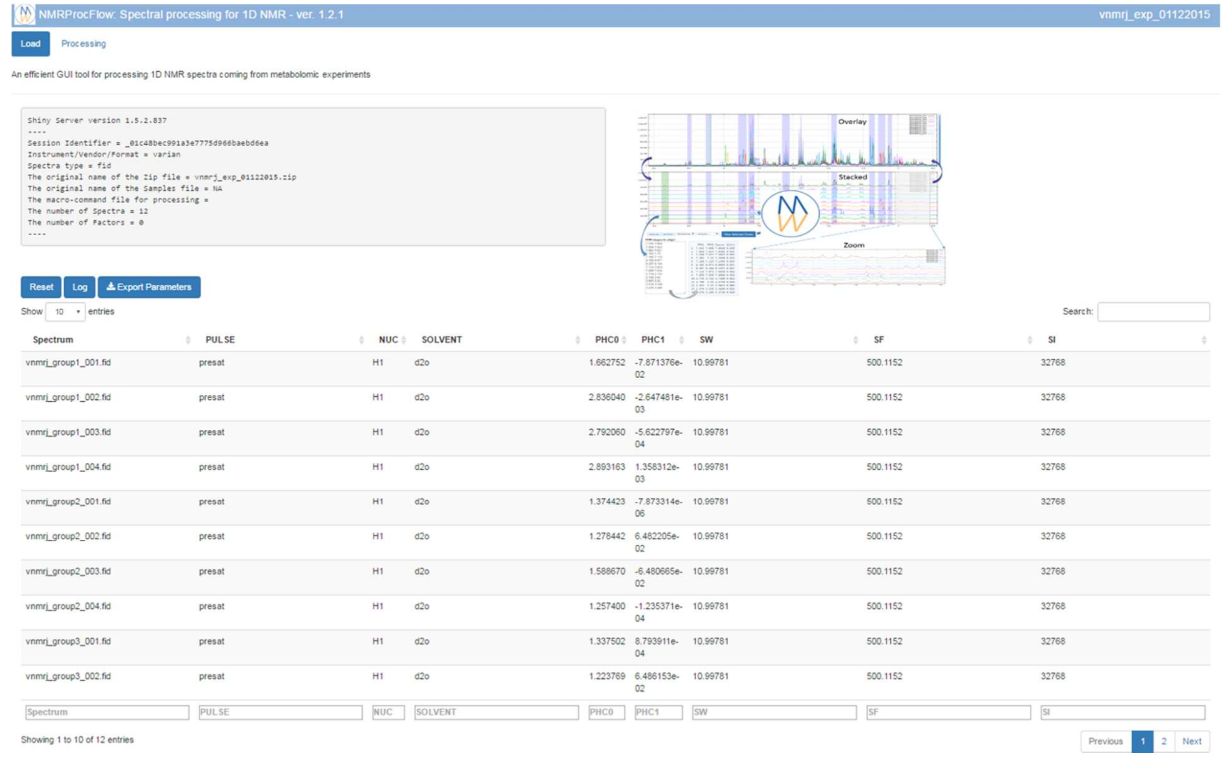

Spectrum	PULSE	NUC	SOLVENT	PHC0	PHC1	SW	SF	SI
vnmrj_group1_001.fid	presat	H1	d2o	1.662752	-7.871376e-02	10.99781	500.1152	32768
vnmrj_group1_002.fid	presat	H1	d2o	2.836040	-2.647481e-03	10.99781	500.1152	32768
vnmrj_group1_003.fid	presat	H1	d2o	2.792060	-5.622797e-04	10.99781	500.1152	32768
vnmrj_group1_004.fid	presat	H1	d2o	2.893163	1.358312e-03	10.99781	500.1152	32768
vnmrj_group2_001.fid	presat	H1	d2o	1.374423	-7.873314e-06	10.99781	500.1152	32768
vnmrj_group2_002.fid	presat	H1	d2o	1.278442	6.482205e-02	10.99781	500.1152	32768
vnmrj_group2_003.fid	presat	H1	d2o	1.588670	-6.480665e-02	10.99781	500.1152	32768
vnmrj_group2_004.fid	presat	H1	d2o	1.257400	-1.235371e-04	10.99781	500.1152	32768
vnmrj_group3_001.fid	presat	H1	d2o	1.337502	8.793911e-04	10.99781	500.1152	32768
vnmrj_group3_002.fid	presat	H1	d2o	1.223769	6.486153e-02	10.99781	500.1152	32768
Spectrum	PULSE	NUC	SOLVENT	PHC0	PHC1	SW	SF	SI

To switch to the processing steps, you must click on the '**Processing' tab** at the top of the screen.

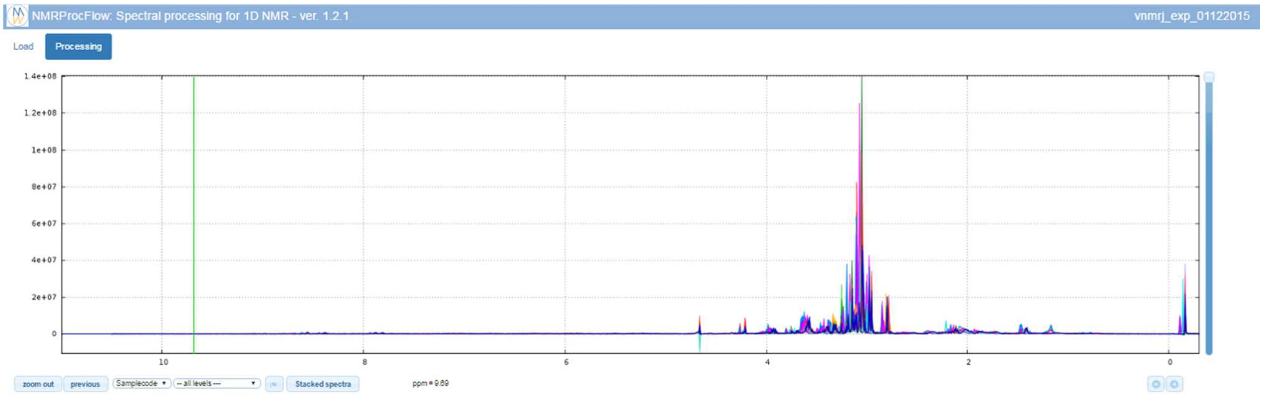

(C) Copyright INRA UMR 1332 BFP - MetaboHub 2016

Spectral pre-processing for 1D NMR

The spectral preprocessing for 1D NMR can be automatically applied in case where the input raw data are FID. The term pre-processing designates here the transformation of the NMR spectrum from time domain to frequency domain, including the phase correction and the Fast Fourier-Transform (FFT). Here, we suggest a reference that could be read with great profit:

James Keeler (2010) Understanding NMR Spectroscopy, 2nd Edition, Ed Wiley

In NMRProcFlow, you can adjust some parameters. Just click on the 'Parameters' button to bring up the window of the parameters to be adjusted, as shown below:

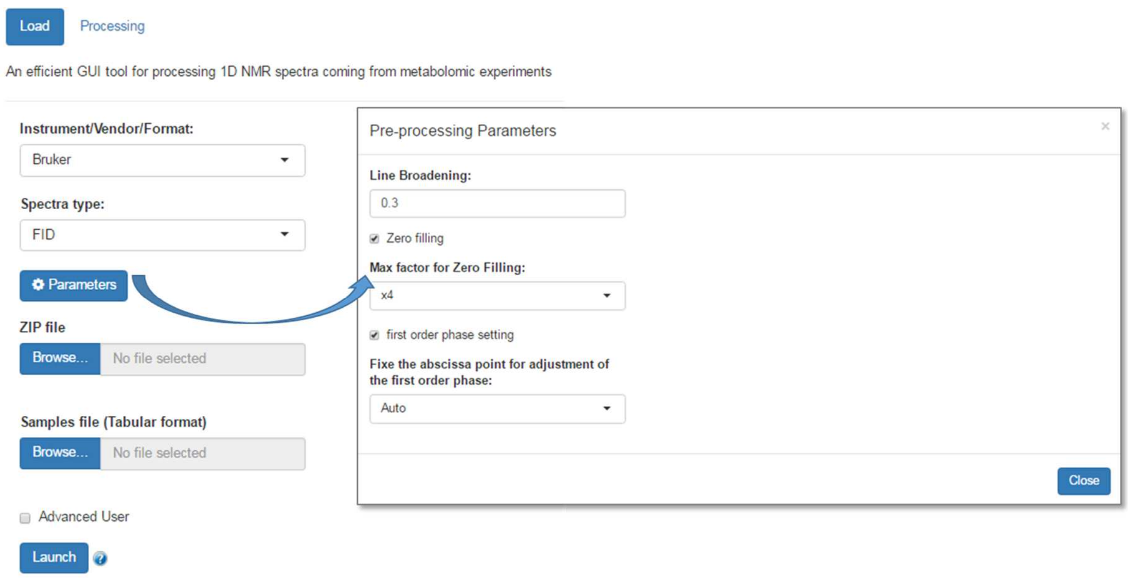

- **Line Broadening**: Apodization is based on a Line Broadening (i.e an exponential) applied on the fid in order to improve the signal-noise ratio. You can modify the parameter (LB). Zero means no apodization. If necessary, by playing very slightly on the LB parameter, sometimes this may greatly improve the phase correction. **Warning**: higher the LB value, poorer the resolution.
- **Zero filling**: This consists of adding zeros at the end of the FID signal such as the resulting size is an even multiple of the initial size. Contrary of an apodization, this has less effect on the spectra resolution, while improving the signal-to-noise ratio.
- **First order phase** can be adjusted (by default) or not. In case of the First order phase needs to be adjusted, the abscissa point (α) used to set the first order phase can be automatically fixed, otherwise you can choose a value in the dropbox list.

Note: You can optimize the parameters to apply on your own raw spectra from the small application online at the URL http://www.bordeaux.inra.fr/pmb/spec/.

(C) Copyright INRA UMR 1332 BFP - MetaboHub 2016

View the spectra

The **NMR spectra viewer** is the central tool of NMRProcFlow and the core of the application. It allows the user:

- To visually explore the spectra either overlaid or stacked,
- To zoom in for intensity and/or ppm scales,
- To color each subset of spectra according to their corresponding factor levels
- To capture a ppm range using the mouse to stick it in the suitable input box in order to process this ppm range

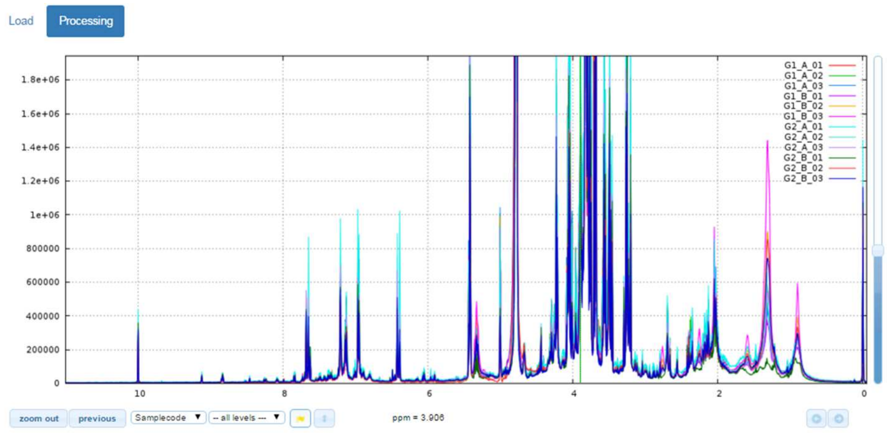

Because the **NMR spectra viewer** is the central tool of NMRProcFlow it occupies more than half of the window.

(C) Copyright INRA UMR 1332 BFP - MetaboHub 2016

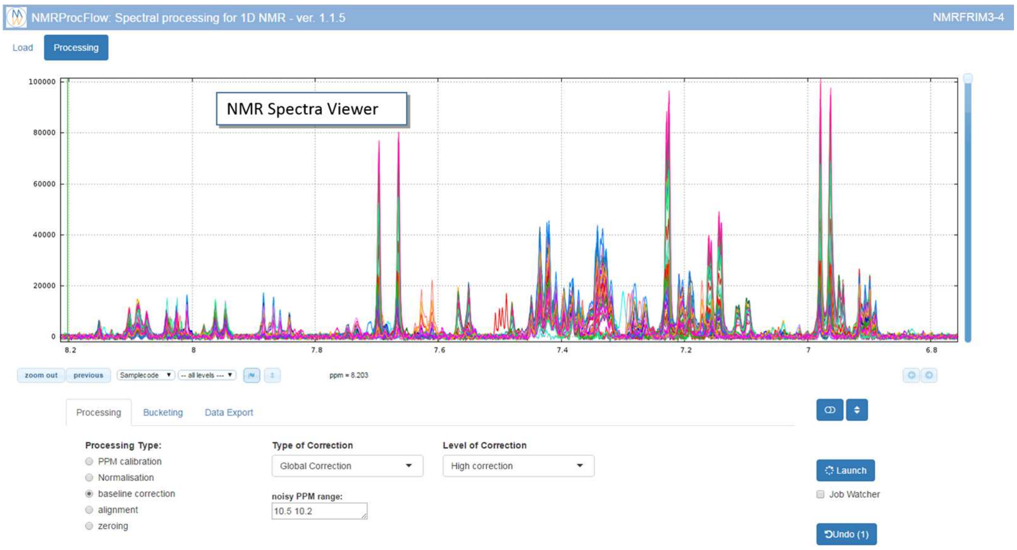

Tip: *For a better view and according to your screen resolution, think about slightly reduce the zoom of your web browser (e.g. 90%)*

Overlay / Stack and Spectra Colors

Overlaid

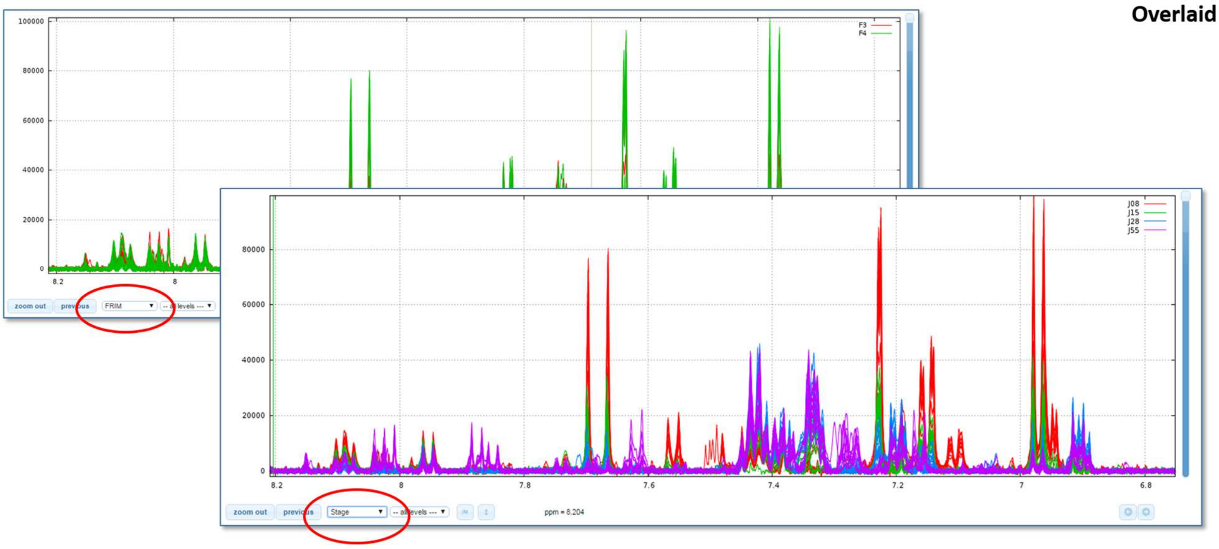

(C) Copyright INRA UMR 1332 BFP - MetaboHub 2016

Stacked

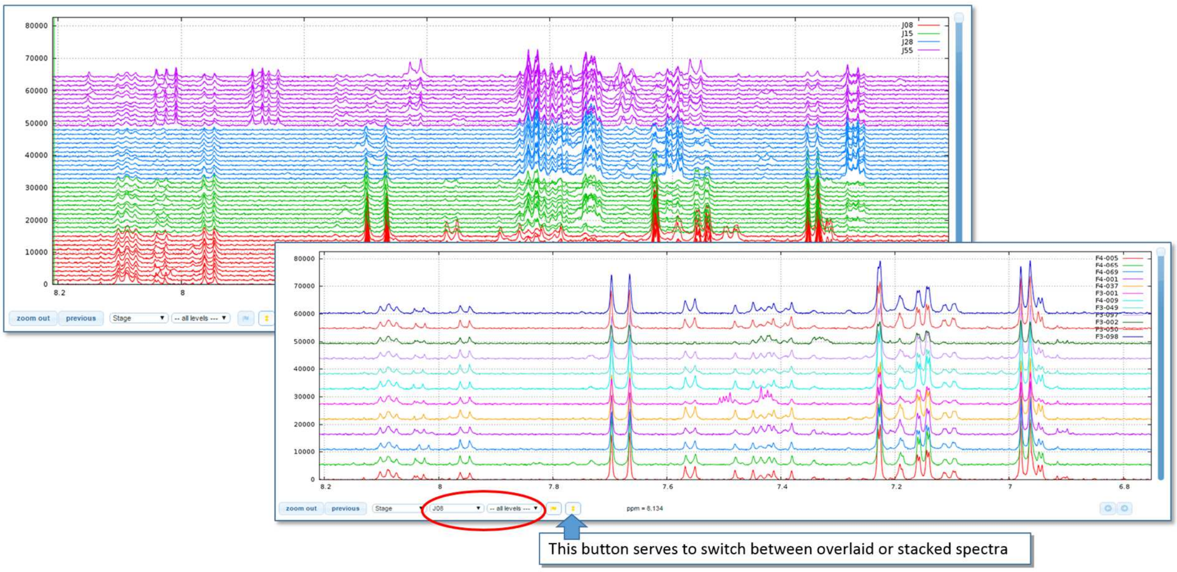

Enlarge the image height

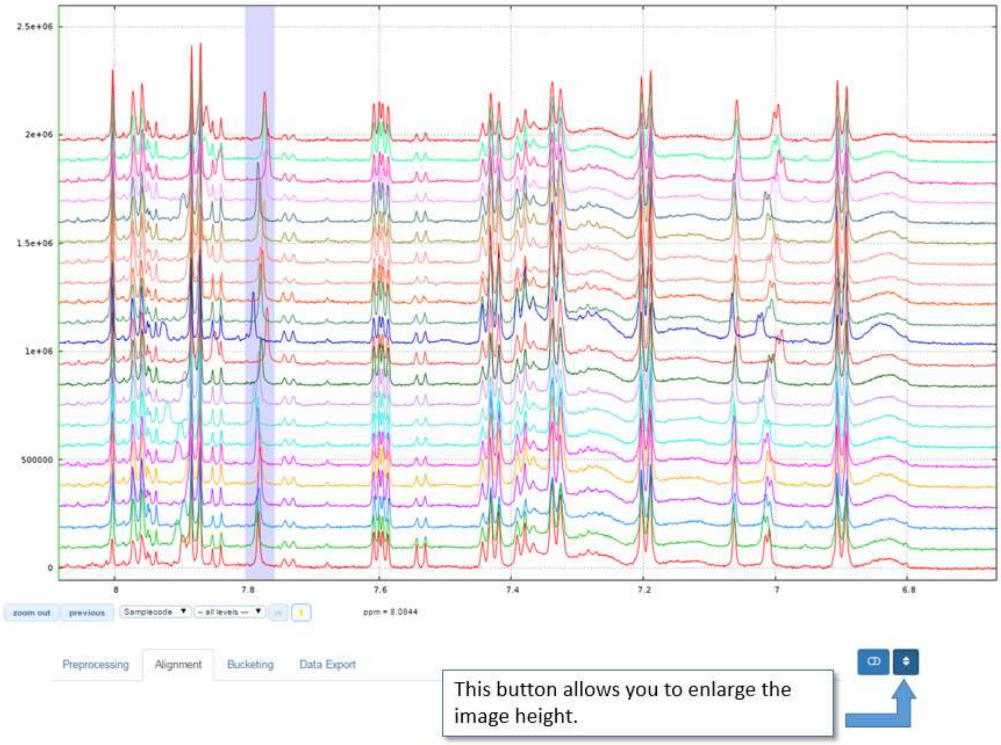

(C) Copyright INRA UMR 1332 BFP - MetaboHub 2016

Interactive data processing

It is possible to navigate between tabs and then **launch any processing in the order you want**.

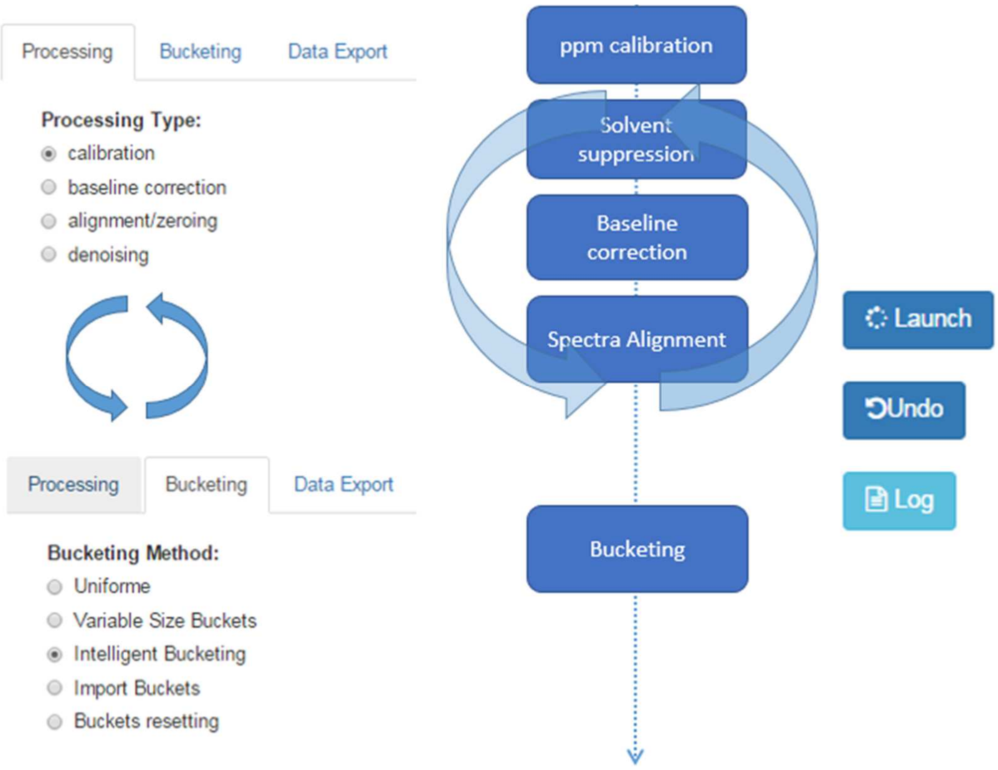

In addition, it is **possible to cancel each treatment performed in the order in which it was made**. The 'Undo' button gives the count of performed processing and that can therefore be cancelled. However, the **bucketing processing can be launched and cancelled independently** of those performed through the 'processing' tab. Indeed, in the latter case, the different kinds of processing involve modifying the spectra themselves, while the bucketing only adds an information layer (the zone of each bucket). A good practice is to launch a bucketing in order to see what the problematic zones are (typically the misalignments). After solving these ones, a bucketing can be launched again in order to see if the problem was correctly solved. If not, the previous processing can be cancelled and replayed with other parameters.

Interaction with the spectra viewer

The processing panel is organized into two distinct areas: 1) the NMR spectra viewer at the top, and 2) the input masks of the different processing modules

(C) Copyright INRA UMR 1332 BFP - MetaboHub 2016

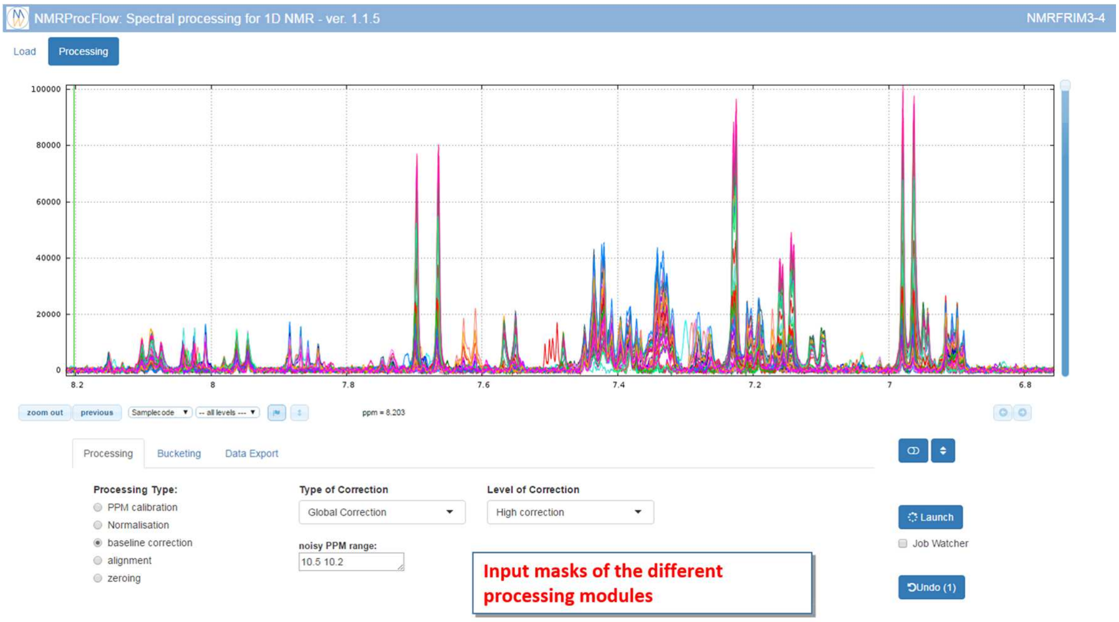

How to capture a ppm range within a box?

A ppm range can be captured using the mouse to stick it in the suitable input box in order to process this ppm range

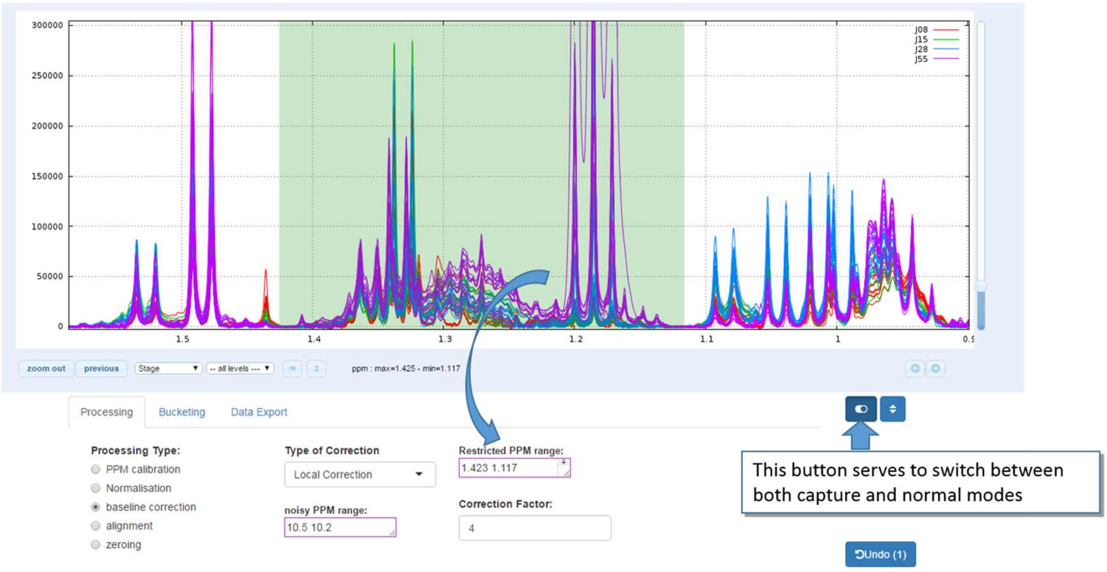

As simple as a copy-paste : Select the ppm zone (copy), then click on the desired box (paste)

(C) Copyright INRA UMR 1332 BFP - MetaboHub 2016

To select a ppm range:

- Toggle into 'capture' mode, then press down the left mouse button at the start of the range, then move the mouse up to the end of the range, then release the mouse button.
- Click on the textbox corresponding to the type of process you want to apply to this range. Textboxes that accept such ppm range capture have a purple outline.

Note: Instead of using the switch button, another way is to press down the 'Ctrl' key and maintain it down while you copy-paste the ppm zone, then release the key up.

How to capture / save images of the NMR spectra on your disk space?

Simply by clicking on the right button of your mouse just on the spectra viewer (preferably at the ppm graduations) and the context menu appear, as shown below:

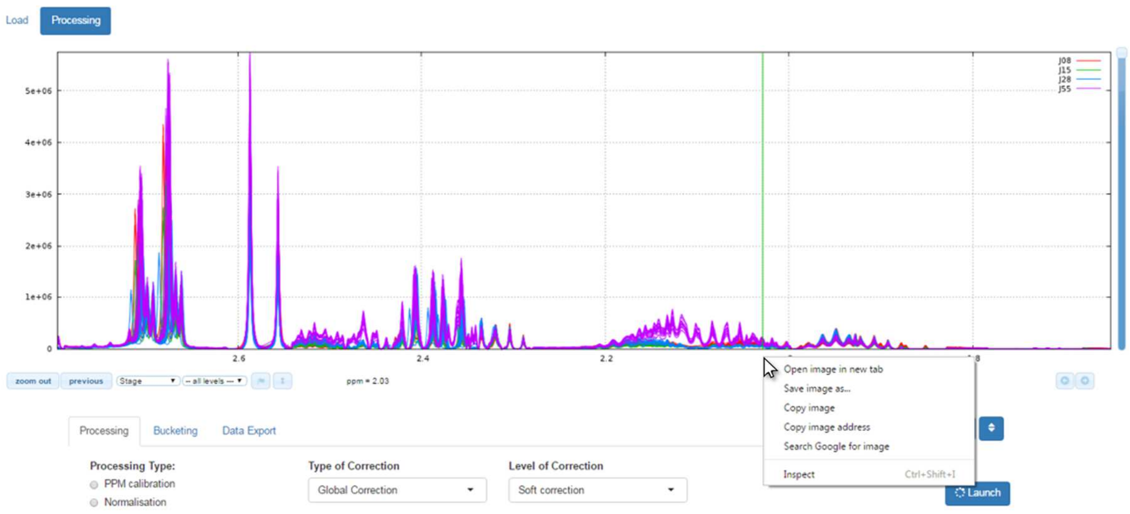

(C) Copyright INRA UMR 1332 BFP - MetaboHub 2016

Spectra processing

PPM calibration

The calibration of the scale of PPM is to adjust the chemical shifts according to a known reference. The reference compound used for chemical shift (δ=0.00) is usually the sodium salt of 3-trimethylsilylpropionic acid-d4 (TSP-d4) with deuterated methylene groups. Other references standards are 2,2- 23 dimethyl-2-silapentane-5-sulfonate sodium salt (DSS) or for organic solvent trimethylsilane (TMS). But it may be any other compound such as creatinine (4.06ppm), α-glucose (5.23ppm), alanine (a doublet along with a peak at 1.488ppm), etc...

How to proceed (see fig below)

- Capture the reference peak into the ‘Range of the PPM reference’ box
- Specify the ppm value corresponding to the highest intensity of the reference peak
- Then launch

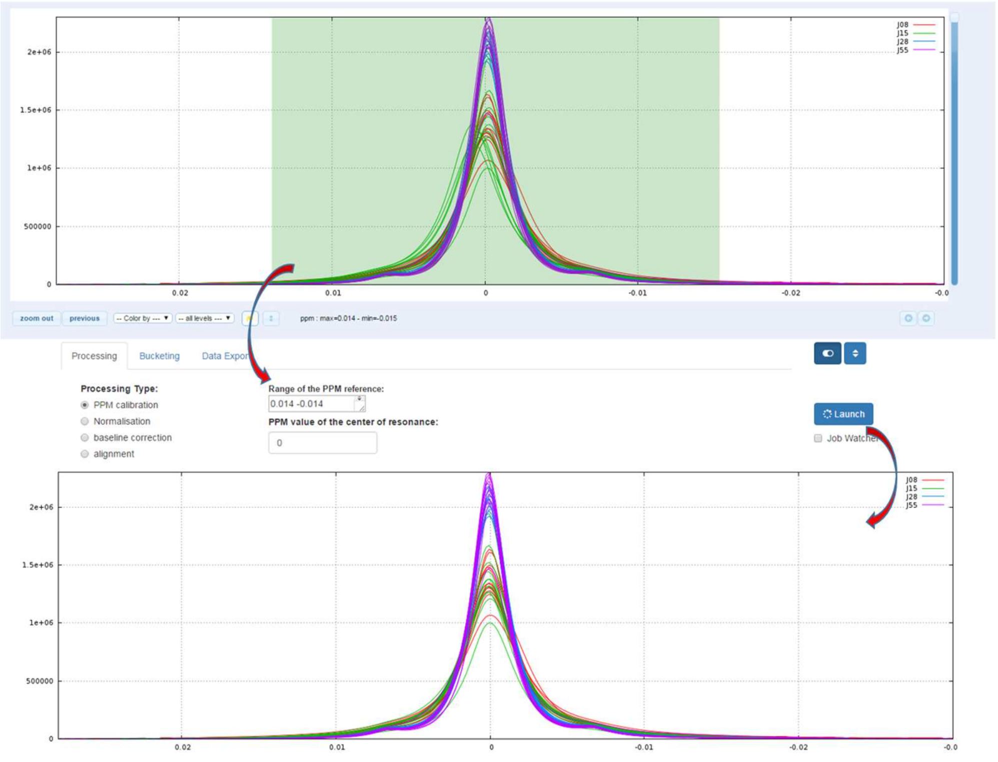

(C) Copyright INRA UMR 1332 BFP - MetaboHub 2016

Baseline Correction

Because on the one hand the peak integration is very sensitive to baseline distortions and on the other hand the baseline distortions may not affect in the same way each spectrum, we have to apply a baseline correction.

Two types of Baseline correction were implemented: **Global** and **Local**. To be more efficient, both methods need to estimate the noise level, and by default the ppm range included between 10.2 and 10.5 ppm is taken. But you can choose another one if some signal is present in this area.

1 - Global

The global baseline correction was based on [Bao et al, 2012], but only two phases were implemented: i) Continuous Wavelet Transform (CWT) and ii) the sliding window algorithm. The user must choose the correction level, from 'soft' up to 'high'.

How to proceed (see fig below)

- Choose the correction level, from 'Soft correction' up to 'High correction'
- Capture the ppm range in order to estimate the noise level
- Then launch

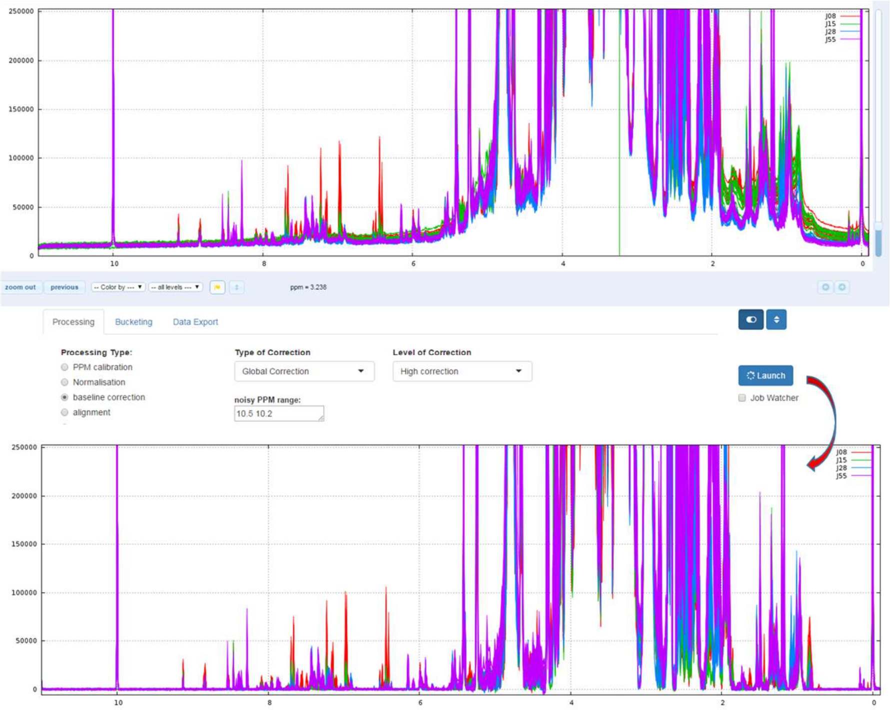

Bao et al (2012) A new baseline correction method based on iterative method, Journal of Magnetic Resonance 218 (2012) 35-43

(C) Copyright INRA UMR 1332 BFP - MetaboHub 2016

2 - Local

The airPLS (adaptive iteratively reweighted penalized least squares) algorithm based on [Zhang et al, 2010] is a baseline correction algorithm which works completely on its own, and that does only require a detail parameter for the algorithm, called Lambda. Because this Lambda parameter can vary within a very large range (from 10 up to 1.e+06), we converted this parameter within a more convenient scale for the user, called 'level correction factor' chosen by the user from '1' (soft) up to '6' (high). The lower this level correction factor is set the smoother baseline will be. Conversely, the higher this level correction factor is set, the more baseline will be corrected in details. To be more efficient, the algorithm needs to estimate the noise level, by default the spectral or ppm range considered, is between 10.2 and 10.5 ppm. But user can choose another one spectral range if some signal is present in this area.

How to proceed (see fig below)

- Capture the ‘Range of the PPM’ to be corrected
- Choose the correction level, from '1' up to '7'
- Capture the ppm range in order to estimate the noise level
- Then launch

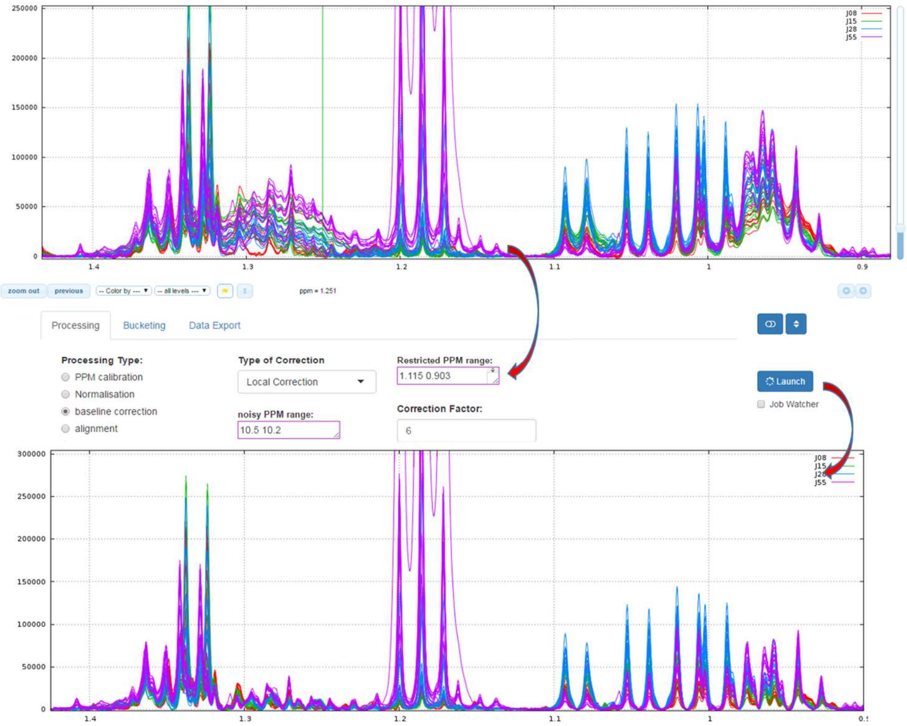

Wehrens R. (2011) Chemometrics with R: Multivariate Data Analysis in the Natural Sciences and Life Sciences, Ed Springer-Verlag Berlin Heidelberg

Zhang Z, Chen S, and Liang Y-Z (2010) Baseline correction using adaptive iteratively reweighted penalized least squares, Analyst, 2010,135, 1138-1146. doi:10.1039/B922045C

(C) Copyright INRA UMR 1332 BFP - MetaboHub 2016

Spectra alignment

The alignment step is undoubtedly one of the most tedious to solve. The misalignments are the results of changes in chemical shifts of NMR peaks largely due to differences in pH and other physicochemical interactions. To solve this prickly problem, we implement two alignment methods, one based on a **Least-Squares algorithm** and the other based on a **Parametric Time Warping** (PTW). Compliant with the NMRProcFlow philosophy and due to the diversity of problems encountered we chose for spectra alignment, the interactive approach. It means interval by interval, each interval being chosen by the user.

1 - Least- Squares algorithm

To align a set of spectra, we **need to choose or to define a reference spectrum**. You can align spectra either based on a particular spectrum chosen within the spectra set, or based on the average spectrum. In this latter case, the re-alignment procedure is executed three times, the average spectrum being recalculated at each time

In order to **limit the relative ppm shift between the spectra to be realigned and the reference spectrum**, you can set this limit by adjusting the parameter 'Relative shift max.', that sets the maximum shift between spectra and the reference. **The range goes from 0 (no ppm shift allowed) up to 1 (maximum ppm shift equal to 100% of the selected ppm range)**

1.1 - How to proceed (see fig below)

- Capture the 'Range of the PPM' to be aligned
- Set the 'Relative max/ shift'
- Choose the 'Reference Spectrum'
- Then launch

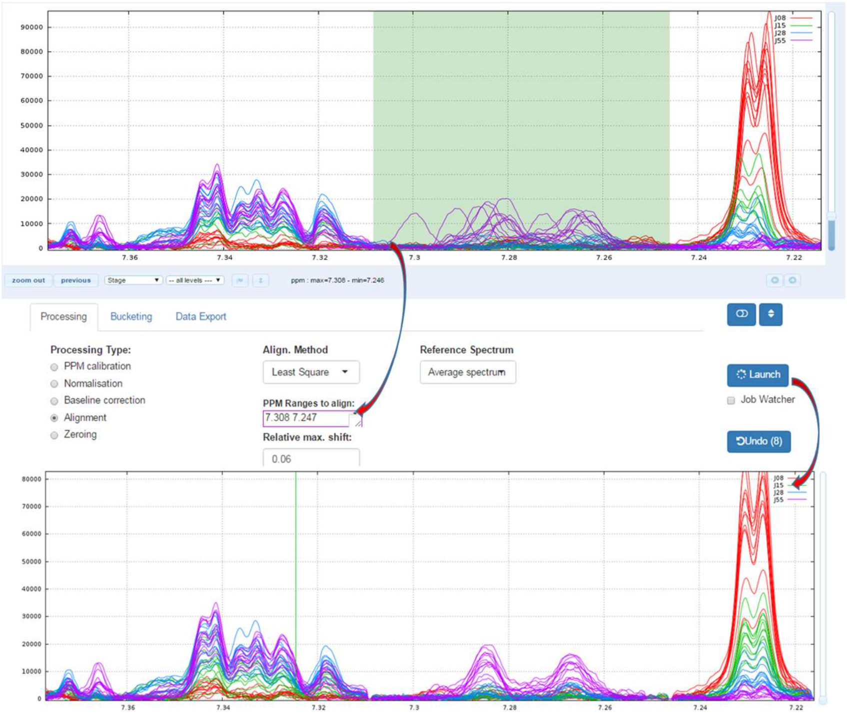

(C) Copyright INRA UMR 1332 BFP - MetaboHub 2016

1.2 - Example showing the importance of the "Relative max. shift" parameter (see fig below)

Consider that we want to align spectra within the ppm range defined by the window as shown below:

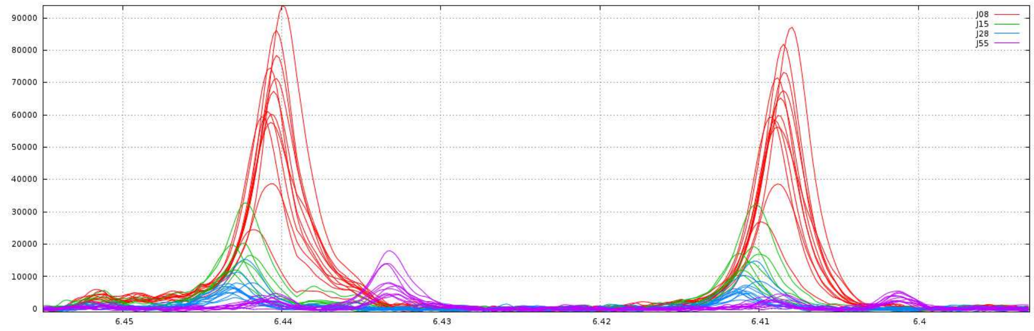

With no way to limit the shifts between spectra, (ie. this corresponds to a relative maximum shift equal to 100%), we have the following result:

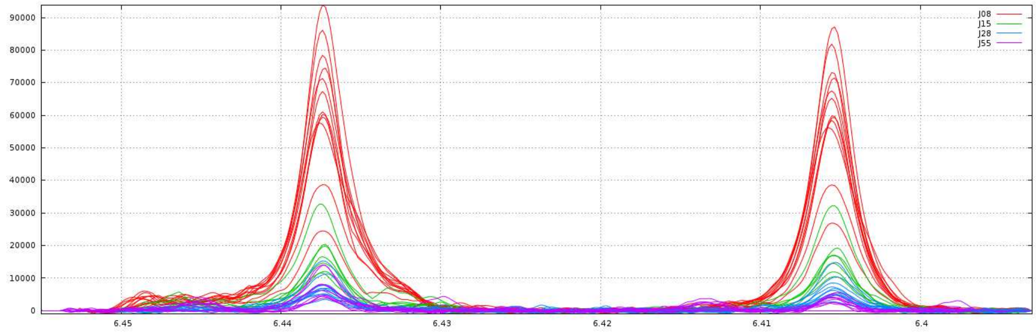

Clearly, it is not the right result. So, we set now a relative maximum shift equal to 5% (ie 0.05), and we have the right result, as shown below:

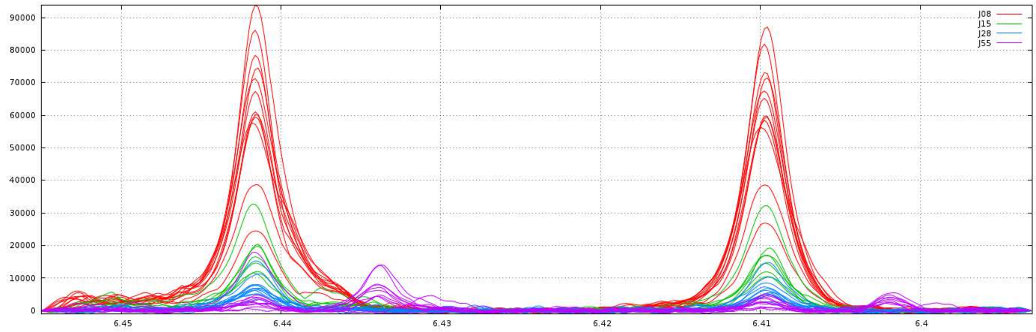

(C) Copyright INRA UMR 1332 BFP - MetaboHub 2016

2 - Parametric Time Warping

The modus operandi for this method is very similar to the previous one, apart the "Relative max. shift" parameter that is not needed. The implementation is based on the R package 'ptw' (Bloemberg et al. 2010) and on the valuable explanations in Wehrens R. (2011).

2.1 - Example of comparison between the Least-Square and PTW methods (see fig below)

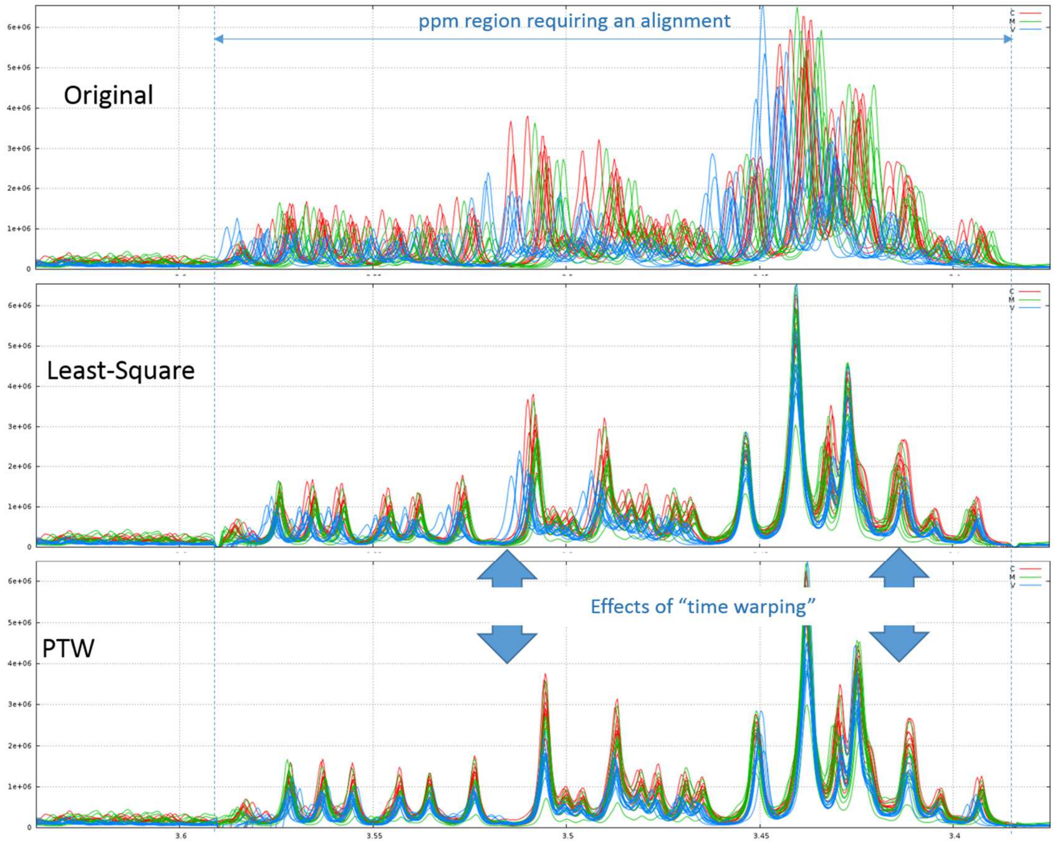

32 1H NMR spectra of fish liver extracts and according 3 diets (500MHz, zg, D20)

Clearly, in this example, PTW method is more efficient than a simple Least-Square approach.

Warnings: Wehrens R. (2011) highlights the fact that (§ 3.3.2) "alignment methods that are too flexible (such as PTW) may be led astray by the presence [so by the absence] of extra peaks, especially when these are of high intensity". Therefore, a "very esthetic alignment" must not be the only quality criterion.

(C) Copyright INRA UMR 1332 BFP - MetaboHub 2016

A good example is shown in the figure below. We first align the spectra in two steps using the Least-Square approach. The orange arrows show the absence of one peak for one stage (J03). Then, we undo the second alignment of the zone that included the problematic peak and we align again this zone using the PTW approach. All spectra are seem well-aligned apart those corresponding to the J03 stage. The red arrows show the zones where the PTW algorithm has been "astray".

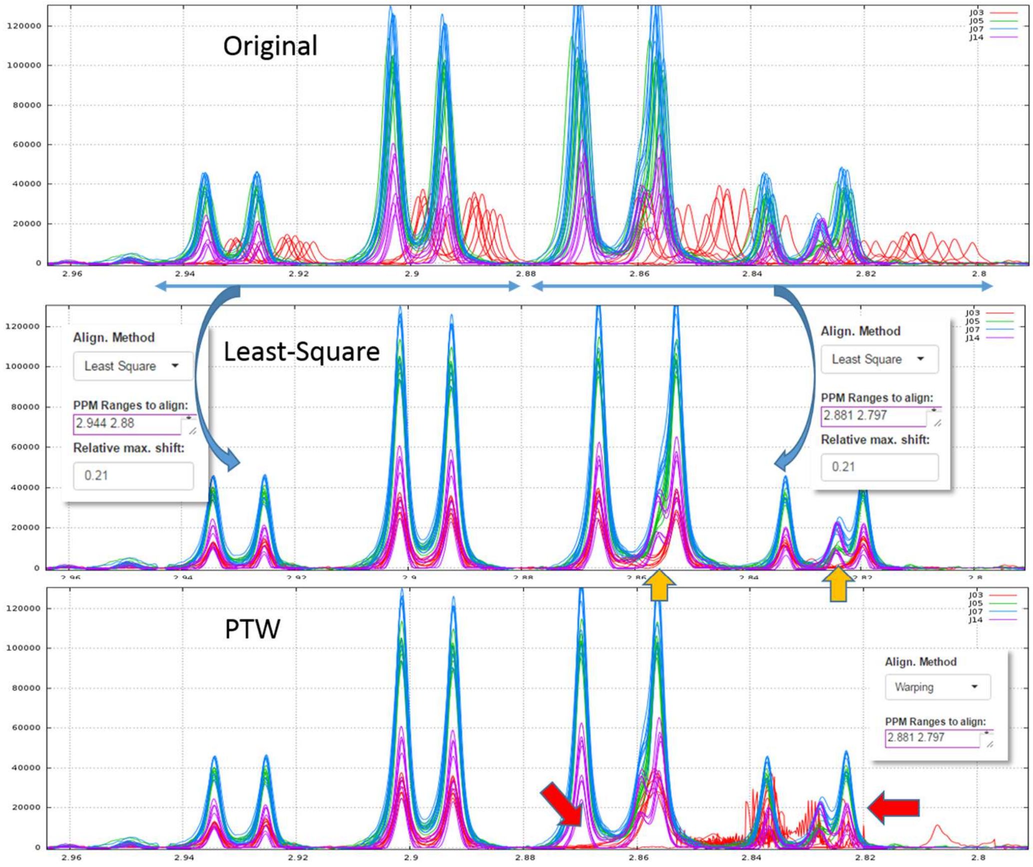

32 1H NMR spectra of supernatant of Fusarium and according 4 stages (500MHz, noesy, D20)

T.G. Bloemberg, J. Gerretzen, H.J.P. Wouters, J. Gloerich, M. van Dael, H.J.C.T. Wessels, L.P. van den Heuvel, P.H.C. Eilers, L.M.C. Buydens, and R. Wehrens. Improved parametric time warping for proteomics. Chemom. Intell. Lab. Systems, 2010.

Wehrens R. (2011). Chemometrics with R: Multivariate Data Analysis in the Natural Sciences and Life Sciences, Ed Springer-Verlag Berlin Heidelberg

(C) Copyright INRA UMR 1332 BFP - MetaboHub 2016

Bucketing

Intelligent Bucketing

An NMR spectrum may contain several thousands of points, and therefore of variables. In order to reduce the data dimensionality binning is commonly used. In binning the spectra are divided into bins (so called buckets) and the total area within each bin is calculated to represent the original spectrum. The more simple approach consists to divide all the spectra with uniform areas width (typically 0.04 ppm). Due to the arbitrary division of peaks, one bin may contain pieces from two or more peaks which may affect the data analysis. We have chosen to implement the Adaptive, Intelligent Binning method [De Meyer et al. 2008] that attempt to split the spectra so that each area common to all spectra contains the same resonance, i.e. belonging to the same metabolite. In such methods, the width of each area is then determined by the maximum difference of chemical shift among all spectra.

How to proceed (see fig below)

- Specify a relevant zone in order to estimate the noise level.
- Choose a resolution factor between 0.1 and 0.6 (0.5 is the default value); the smaller value the greater resolution
- Select one or more PPM zones for applying the binning and put them in the box of 'PPM ranges'
- Choose the threshold of the Signal/Noise Ratio (SNR) so that the buckets having a lower average integration will be excluded.
- Then launch

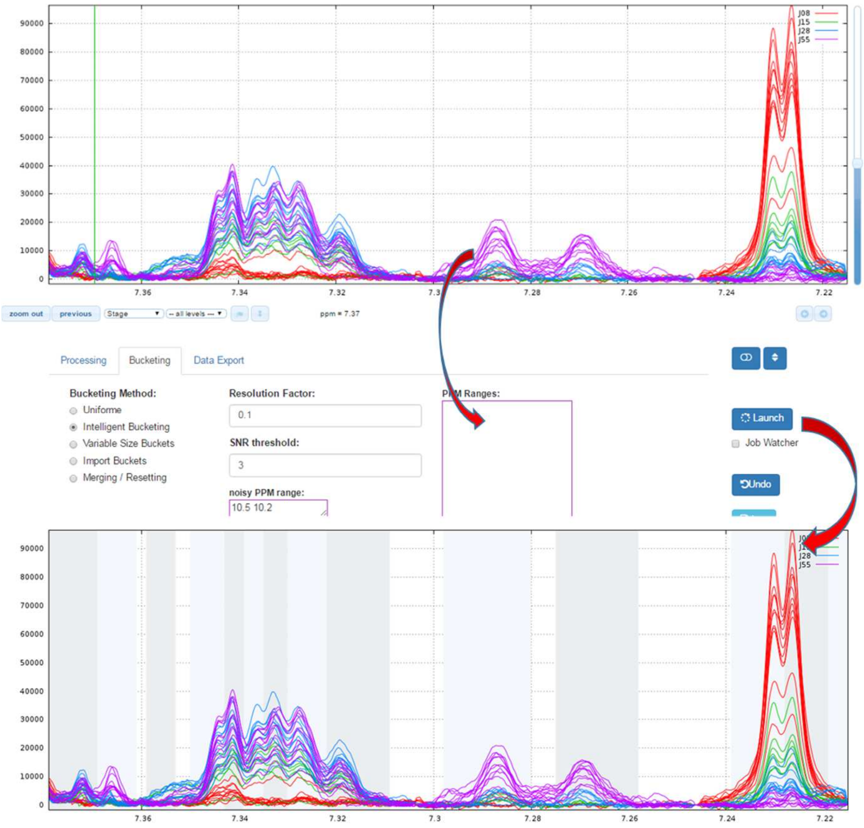

(C) Copyright INRA UMR 1332 BFP - MetaboHub 2016

Warnings:Do not choose a resolution parameter too small in an area where alignment has not been made. NMRProcFlow makes it possible to adapt area by area the right resolution.

de Meyer T, Sinnaeve D, van Gasse B, Tsiporkova E, Rietzschel E, de Buyzere M, Gillebert T, Bekaert S, Martins J, van Criekinge W (2008) NMR-based characterization of metabolic alterations in hypertension using an adaptive, intelligent binning algorithm. Anal Chem 80:3783–3790

Variable Size Buckets

Then it is always possible to make some adjustments by merging or resetting one or more buckets.

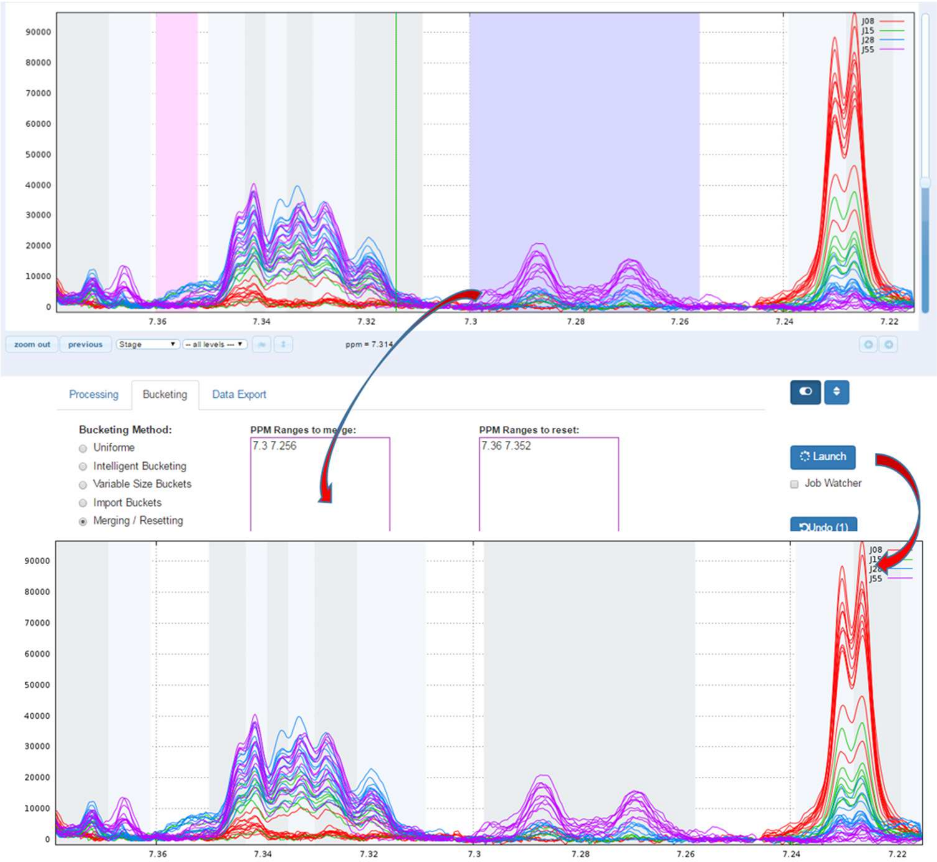

(C) Copyright INRA UMR 1332 BFP - MetaboHub 2016

Import Buckets

Another way for obtain buckets is to choose yourself ppm ranges you want to integrate. This method is typically used for the Targeted metabolomics approach where only few peaks corresponding to targeted compounds are selected whose their size is depending of the signal pattern.

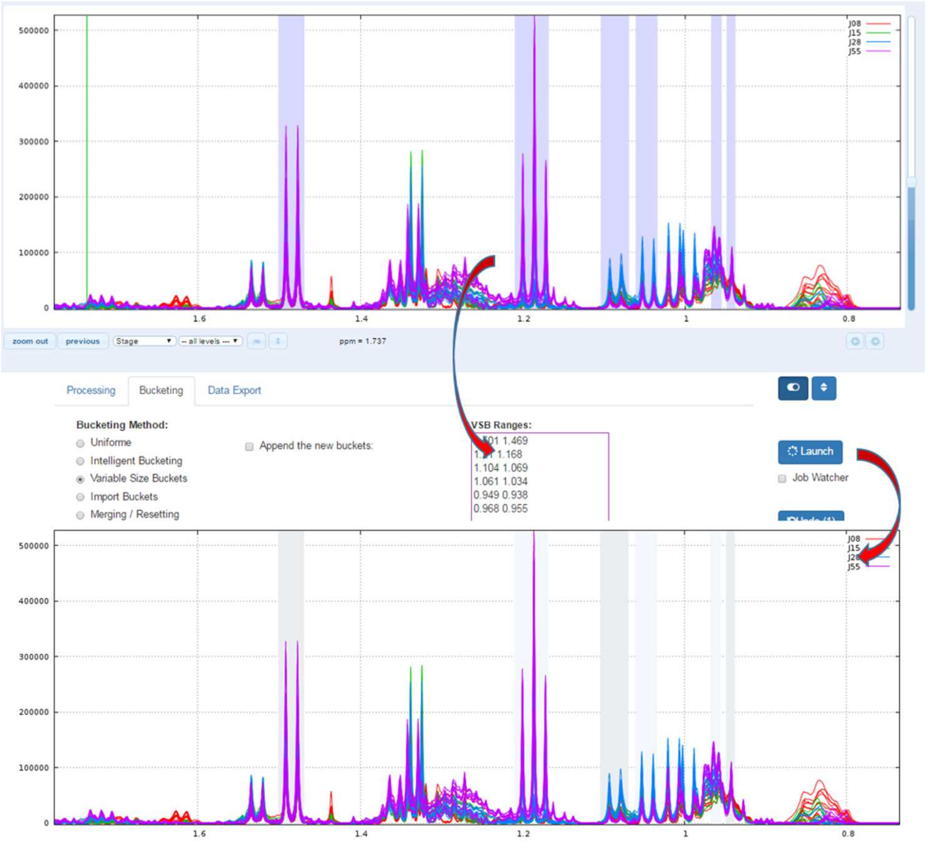

(C) Copyright INRA UMR 1332 BFP - MetaboHub 2016

Merging / Resetting

An exported buckets table can be imported into NMRProcFlow in order to retrieve the same bucketing obtained in a previous work session

How to proceed (see fig below)

- Choose the format corresponding of the imported file
- Check if the imported file have an header line or no
- Choose the corresponding columns for the lower and upper ppm bounds of the buckets.
- Check if imported buckets will be append to those already defined or not. If not, all buckets previously defined will be erased.
- Then launch

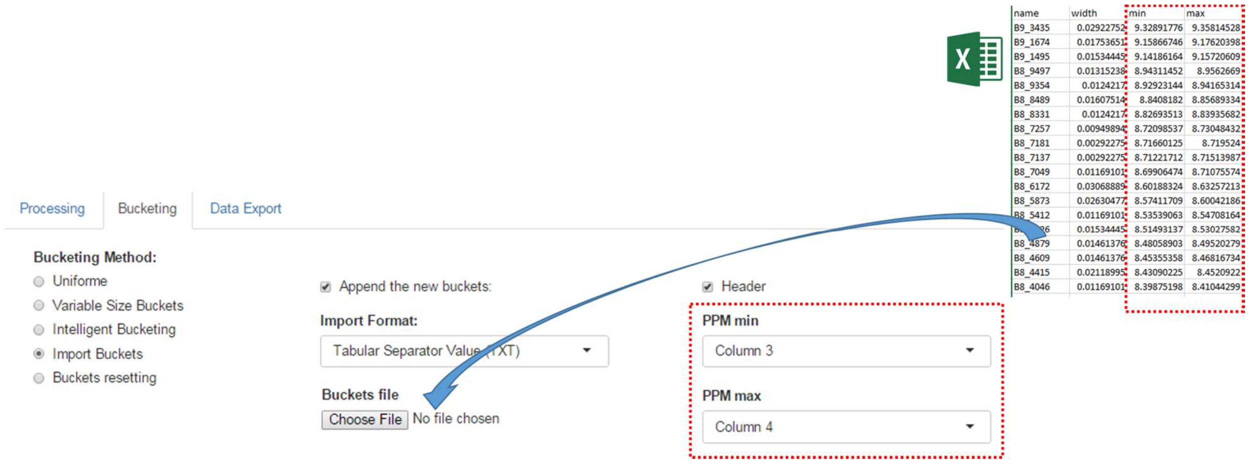

(C) Copyright INRA UMR 1332 BFP - MetaboHub 2016

Data Export

Data Matrix

Before exporting, in order to make all spectra comparable each other, we have to account for variations of the overall concentrations of samples. In NMR metabolomics, the total intensity normalization (called the Constant Sum Normalization) is often used so that all spectra correspond to the same overall concentration. It simply consists to normalize the total intensity of each individual spectrum to a same value. But other methods such as Probabilistic Quotient Normalization [Dieterle et al. 2006] assumes that biologically interesting concentration changes influence only parts of the NMR spectrum, while dilution effects will affect all metabolite signals. Probabilistic Quotient Normalization (PQN) starts by the calculation of a reference spectrum based on the median spectrum. Next, for each variable of interest the quotient of a given test spectrum and reference spectrum is calculated and the median of all quotients is estimated. Finally, all variables of the test spectrum are divided by the median quotient. We suggest the reference [Kohl et al. 2012] as a good review that could be read with great profit.

An internal reference can be used to normalize the data. Typically, an Electronic reference (ERETIC) can be used for that (see Akoka et al. 1999). Integral value of each bucket will be divided by the integral value of the PPM range given as reference..

How to proceed (see fig below)

- Choose a Normalization Method
- Choose an Export Format
- Choose the threshold of the Signal/Noise Ratio (SNR) so that the buckets having a lower average integration will be excluded.
- Specify if necessary, the PPM range of the internal reference signal (typically, the ERETIC signal). Otherwise, leave empty this box.
- Then, click on Export

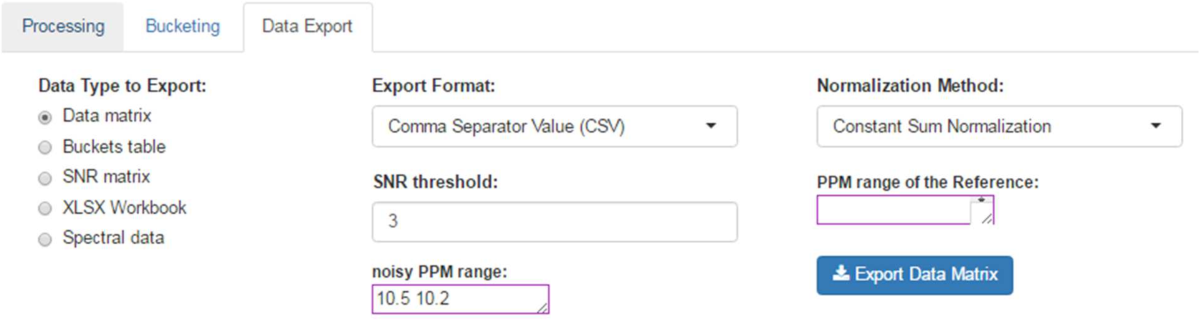

After exporting, the data matrix is formatted so that we can subsequently perform statistical analysis using BioStatFlow web application. Thus the data file manipulations are minimized. See Metabolic Fingerprinting

(C) Copyright INRA UMR 1332 BFP - MetaboHub 2016

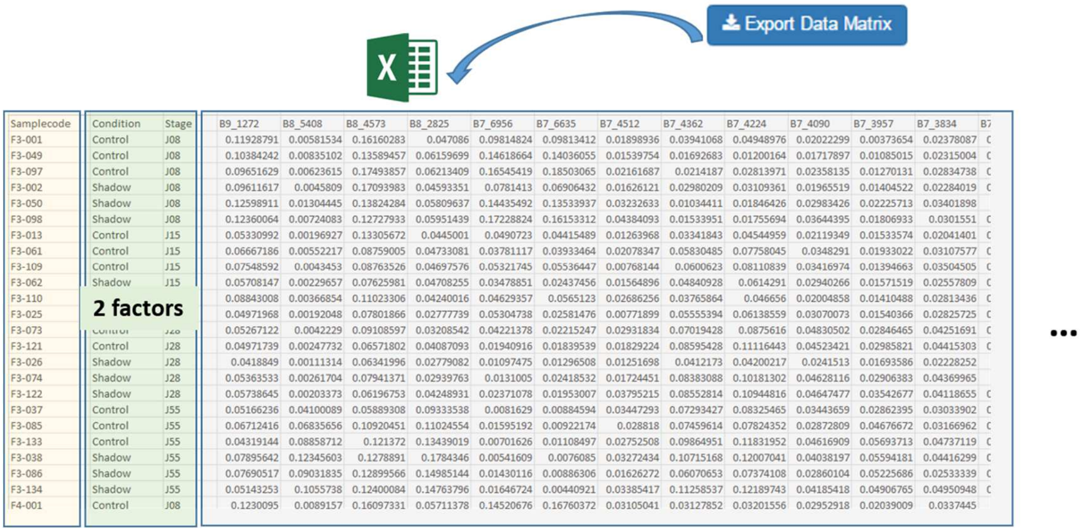

Note that the factors are embedded in the file, provided they have been specified in the first step (file samples)

Dieterle F., Ross A., Schlotterbeck G. and Senn H. (2006). Probabilistic Quotient Normalization as Robust Method to Account for Dilution of Complex Biological Mixtures. Application in 1H NMR Metabonomics. Analytical Chemistry, 78:4281-4290.

Kohl SM, Klein MS, Hochrein J, Oefner PJ, Spang R, Gronwald W. (2012) State-of-the art data normalization methods improve NMR-based metabolomic analysis, Metabolomics 146-160, DOI:10.1007/s11306-011-0350-z

Akoka S1, Barantin L, Trierweiler M. (1999) Concentration Measurement by Proton NMR Using the ERETIC Method., Anal. Chem 71(13):2554-7. doi: 10.1021/ac981422i.

Buckets table

The buckets table can be exported in order to be saved on your local space disk. Then it can be used as an association file along with the data matrix within BioStatFlow, or be imported into NMRProcFlow (see below)

(C) Copyright INRA UMR 1332 BFP - MetaboHub 2016

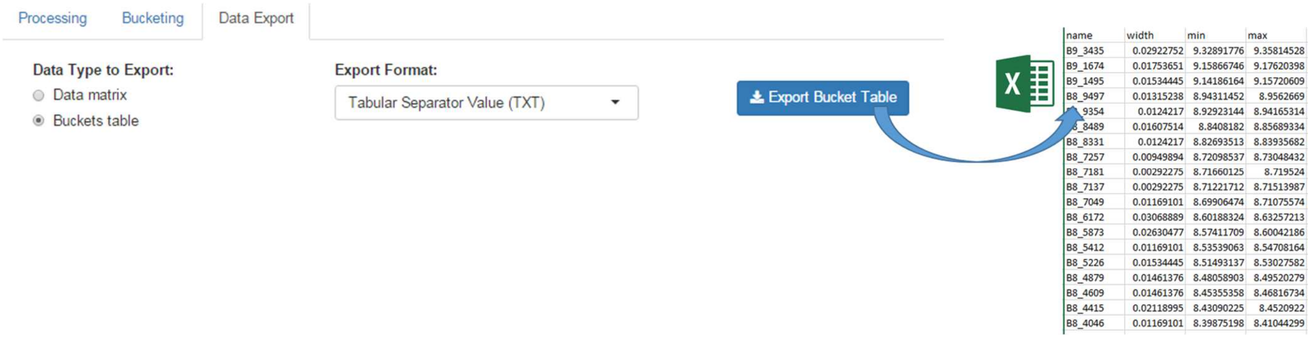

name	width	min	max
B9_3435	0.02922752	9.32891776	9.35814528
B9_1674	0.01753651	9.15866746	9.17620398
B9_1495	0.01534445	9.14186164	9.15720609
B8_9497	0.01315238	8.94311452	8.9562669
[illegible]_9354	0.0124217	8.92923144	8.94165314
[illegible]_8489	0.01607514	8.8408182	8.85689334
B8_8331	0.0124217	8.82693513	8.83935682
B8_7257	0.00949894	8.72098537	8.73048432
B8_7181	0.00292275	8.71660125	8.719524
B8_7137	0.00292275	8.71221712	8.71513987
B8_7049	0.01169101	8.69906474	8.71075574
B8_6172	0.03068889	8.60188324	8.63257213
B8_5873	0.02630477	8.57411709	8.60042186
B8_5412	0.01169101	8.53539063	8.54708164
B8_5226	0.01534445	8.51493137	8.53027582
B8_4879	0.01461376	8.48058903	8.49520279
B8_4609	0.01461376	8.45355358	8.46816734
B8_4415	0.02118995	8.43090225	8.4520922
B8_4046	0.01169101	8.39875198	8.41044299

The exported buckets table can be imported into NMRProcFlow in order to retrieve the same bucketing obtained in a previous work session. See Import Buckets

SNR matrix

The **Signal-Noise Ratio** (SNR) matrix can be exported, as shown below

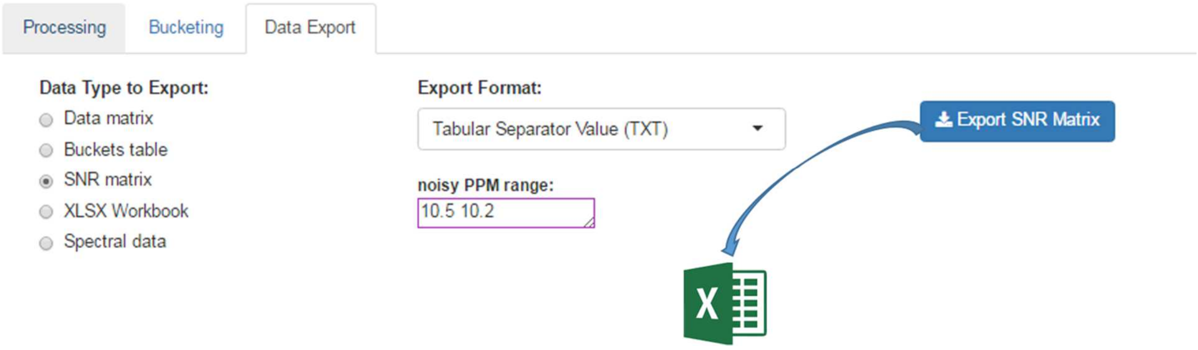

(C) Copyright INRA UMR 1332 BFP - MetaboHub 2016

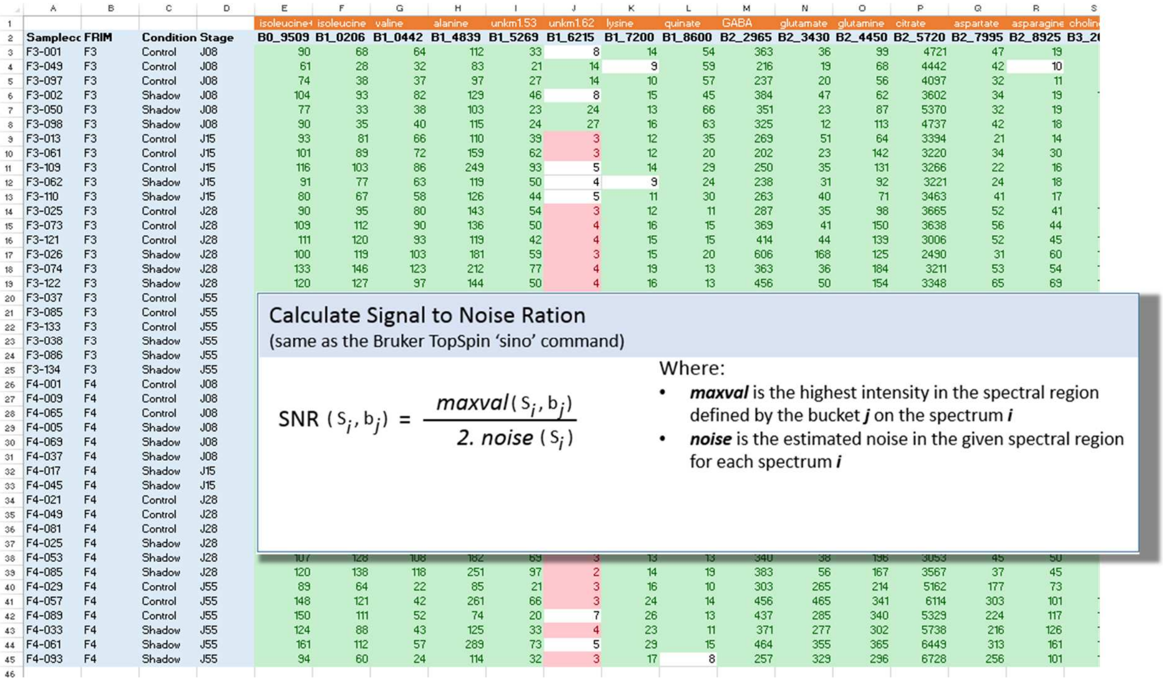

Sampleco	FRIM	Condition	Stage	isoleucine	isoleucine	valine	alanine	unkm1.53	unkm1.62	lysine	quinate	GABA	glutamate	glutamine	citrate	aspartate	asparagine
				B0_9509	B1_0206	B1_0442	B1_4839	B1_5269	B1_6215	B1_7200	B1_8600	B2_2965	B2_3430	B2_4450	B2_5720	B2_7995	B2_8925
F3-001	F3	Control	J08	90	68	64	112	33	8	14	54	363	36	99	4721	47	19
F3-049	F3	Control	J08	61	28	32	83	21	14	9	59	216	19	68	4442	42	10
F3-097	F3	Control	J08	74	38	37	97	27	14	10	57	237	20	56	4097	32	11
F3-002	F3	Shadow	J08	104	93	82	129	46	8	15	45	384	47	62	3602	34	19
F3-050	F3	Shadow	J08	77	33	38	103	23	24	13	66	351	23	87	5370	32	19
F3-098	F3	Shadow	J08	90	35	40	115	24	27	16	63	325	12	113	4737	42	18
F3-013	F3	Control	J15	93	81	66	110	39	3	12	35	269	51	64	3394	21	14
F3-061	F3	Control	J15	101	89	72	159	62	3	12	20	202	23	142	3220	34	30
F3-109	F3	Control	J15	116	103	86	249	93	5	14	29	250	35	131	3266	22	16
F3-062	F3	Shadow	J15	91	77	63	119	50	4	9	24	238	31	92	3221	24	18
F3-110	F3	Shadow	J15	80	67	58	126	44	5	11	30	263	40	71	3463	41	17
F3-025	F3	Control	J28	90	95	80	143	54	3	12	11	287	35	98	3665	52	41
F3-073	F3	Control	J28	109	112	90	136	50	4	16	15	369	41	150	3638	56	44
F3-121	F3	Control	J28	111	120	93	119	42	4	15	15	414	44	139	3006	52	45
F3-026	F3	Shadow	J28	100	119	103	181	59	3	15	20	606	168	125	2490	31	60
F3-074	F3	Shadow	J28	133	146	123	212	77	4	19	13	363	36	184	3211	53	54
F3-122	F3	Shadow	J28	120	127	97	144	50	4	16	13	456	50	154	3348	65	69
F3-037	F3	Control	J55	[illegible]	[illegible]	[illegible]	[illegible]	[illegible]	[illegible]	[illegible]	[illegible]	[illegible]	[illegible]	[illegible]	[illegible]	[illegible]	[illegible]
F3-085	F3	Control	J55	[illegible]	[illegible]	[illegible]	[illegible]	[illegible]	[illegible]	[illegible]	[illegible]	[illegible]	[illegible]	[illegible]	[illegible]	[illegible]	[illegible]
F3-133	F3	Control	J55	[illegible]	[illegible]	[illegible]	[illegible]	[illegible]	[illegible]	[illegible]	[illegible]	[illegible]	[illegible]	[illegible]	[illegible]	[illegible]	[illegible]
F3-038	F3	Shadow	J55	[illegible]	[illegible]	[illegible]	[illegible]	[illegible]	[illegible]	[illegible]	[illegible]	[illegible]	[illegible]	[illegible]	[illegible]	[illegible]	[illegible]
F3-086	F3	Shadow	J55	[illegible]	[illegible]	[illegible]	[illegible]	[illegible]	[illegible]	[illegible]	[illegible]	[illegible]	[illegible]	[illegible]	[illegible]	[illegible]	[illegible]
F3-134	F3	Shadow	J55	[illegible]	[illegible]	[illegible]	[illegible]	[illegible]	[illegible]	[illegible]	[illegible]	[illegible]	[illegible]	[illegible]	[illegible]	[illegible]	[illegible]
F4-001	F4	Control	J08	[illegible]	[illegible]	[illegible]	[illegible]	[illegible]	[illegible]	[illegible]	[illegible]	[illegible]	[illegible]	[illegible]	[illegible]	[illegible]	[illegible]
F4-009	F4	Control	J08	[illegible]	[illegible]	[illegible]	[illegible]	[illegible]	[illegible]	[illegible]	[illegible]	[illegible]	[illegible]	[illegible]	[illegible]	[illegible]	[illegible]
F4-065	F4	Control	J08	[illegible]	[illegible]	[illegible]	[illegible]	[illegible]	[illegible]	[illegible]	[illegible]	[illegible]	[illegible]	[illegible]	[illegible]	[illegible]	[illegible]
F4-005	F4	Shadow	J08	[illegible]	[illegible]	[illegible]	[illegible]	[illegible]	[illegible]	[illegible]	[illegible]	[illegible]	[illegible]	[illegible]	[illegible]	[illegible]	[illegible]
F4-069	F4	Shadow	J08	[illegible]	[illegible]	[illegible]	[illegible]	[illegible]	[illegible]	[illegible]	[illegible]	[illegible]	[illegible]	[illegible]	[illegible]	[illegible]	[illegible]
F4-037	F4	Shadow	J08	[illegible]	[illegible]	[illegible]	[illegible]	[illegible]	[illegible]	[illegible]	[illegible]	[illegible]	[illegible]	[illegible]	[illegible]	[illegible]	[illegible]
F4-017	F4	Shadow	J15	[illegible]	[illegible]	[illegible]	[illegible]	[illegible]	[illegible]	[illegible]	[illegible]	[illegible]	[illegible]	[illegible]	[illegible]	[illegible]	[illegible]
F4-045	F4	Shadow	J15	[illegible]	[illegible]	[illegible]	[illegible]	[illegible]	[illegible]	[illegible]	[illegible]	[illegible]	[illegible]	[illegible]	[illegible]	[illegible]	[illegible]
F4-021	F4	Control	J28	[illegible]	[illegible]	[illegible]	[illegible]	[illegible]	[illegible]	[illegible]	[illegible]	[illegible]	[illegible]	[illegible]	[illegible]	[illegible]	[illegible]
F4-049	F4	Control	J28	[illegible]	[illegible]	[illegible]	[illegible]	[illegible]	[illegible]	[illegible]	[illegible]	[illegible]	[illegible]	[illegible]	[illegible]	[illegible]	[illegible]
F4-081	F4	Control	J28	[illegible]	[illegible]	[illegible]	[illegible]	[illegible]	[illegible]	[illegible]	[illegible]	[illegible]	[illegible]	[illegible]	[illegible]	[illegible]	[illegible]
F4-025	F4	Shadow	J28	[illegible]	[illegible]	[illegible]	[illegible]	[illegible]	[illegible]	[illegible]	[illegible]	[illegible]	[illegible]	[illegible]	[illegible]	[illegible]	[illegible]
F4-053	F4	Shadow	J28	107	128	108	182	69	3	13	13	340	38	196	3053	45	50
F4-085	F4	Shadow	J28	120	138	118	251	97	2	14	19	383	56	167	3567	37	45
F4-029	F4	Control	J55	89	64	22	85	21	3	16	10	303	265	214	5162	177	73
F4-057	F4	Control	J55	148	121	42	261	66	3	24	14	456	465	341	6114	303	101
F4-089	F4	Control	J55	150	111	52	74	20	7	26	13	437	285	340	5329	224	117
F4-033	F4	Shadow	J55	124	88	43	125	33	4	23	11	371	277	302	5738	216	126
F4-061	F4	Shadow	J55	161	112	57	289	73	5	29	15	464	355	365	6449	313	161
F4-093	F4	Shadow	J55	94	60	24	114	32	3	17	8	257	329	296	6728	256	101

See online some slides about the SNR Exporting (http://nmrprocflow.org/themes/pdf/SNR_export.pdf)

(C) Copyright INRA UMR 1332 BFP - MetaboHub 2016

XLSX Workbook

After the processing and bucketing steps, NMRProcFlow allows users to export all data needed for the quantification in a same XLSX workbook. Two workbook templates were currently available: A simple one and a template dedicated for the quantification.

The simple template just aggregates the buckets table, the SNR matrix and the data matrix, each data type being within a separate tab.

The 'qHNMR' template, in the same way as the simple template aggregates information like the samples table, the buckets table, the SNR matrix and the data matrix within separate tabs, but also includes another tab with the pre-calculated quantifications according to a formula from data provided in the others tabs. Some information are set by default in both 'samples' and 'buckets' tabs. Just adjust them with the appropriate values and the quantifications within the eponymous tab will be automatically updated.

How to proceed (see fig below)

- Choose a template type
- Choose a Normalization Method
- Choose the threshold of the Signal/Noise Ratio (SNR) so that the buckets having a lower average integration will be excluded.
- Specify if necessary, the PPM range of the internal reference signal (typically, the ERETIC signal). Otherwise, leave empty this box.
- Then, click on Export

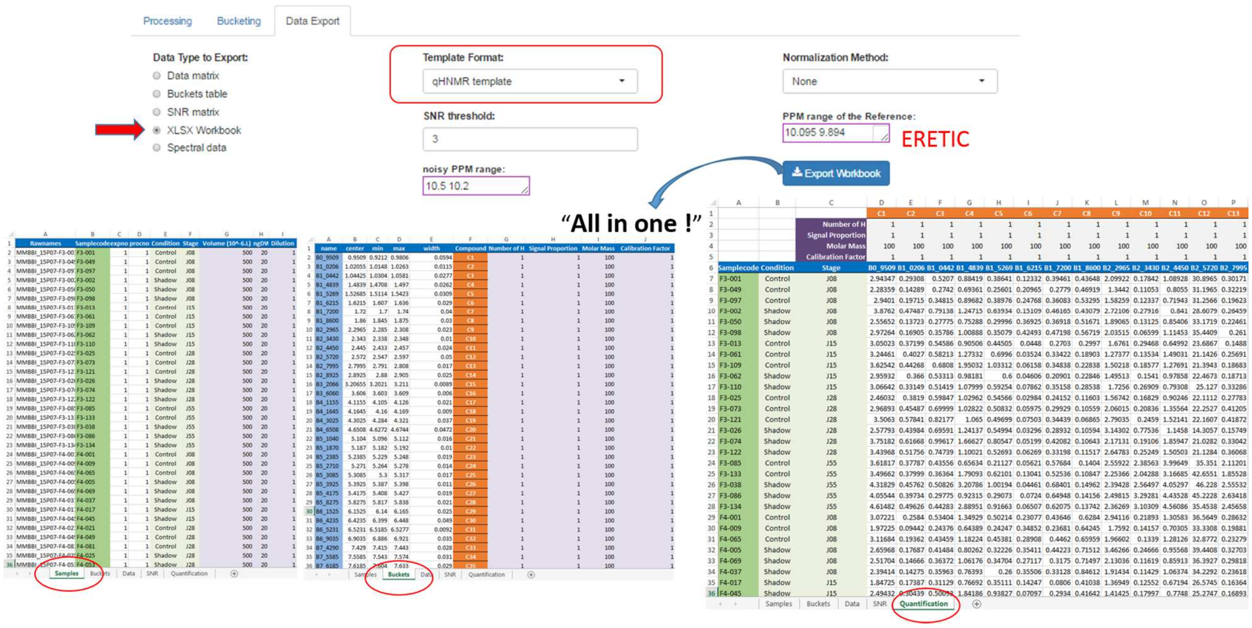

See [Targeted Metabolomics](#) for further information

(C) Copyright INRA UMR 1332 BFP - MetaboHub 2016

Restore a session

Reload a working session

1) Bookmark the URL corresponding to your session as shown in the figure below (Chrome browser). Instead of the URL itself, the chosen name is based on the ZIP name you have upload in your session, giving an easy mnemonic way to retrieve the bookmark in your list (sometimes crowded)

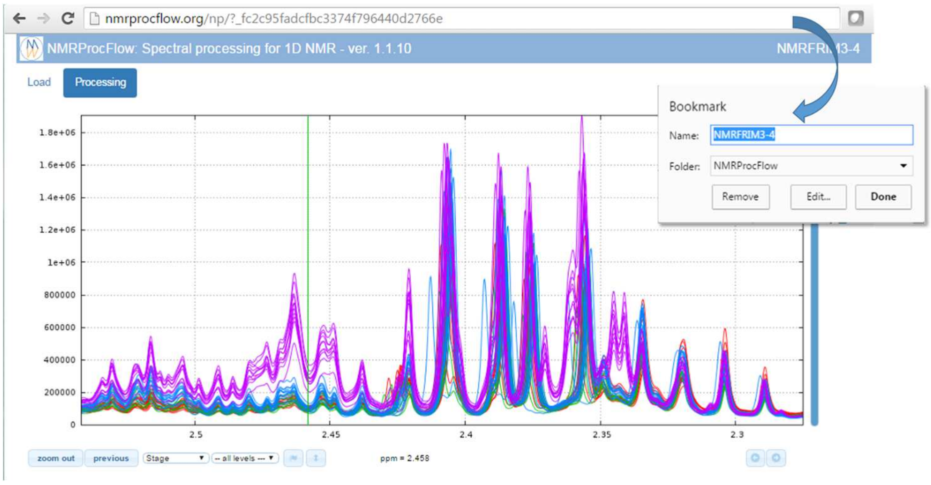

2) Recovering your working session in the same state as you left after few hours or days, depending on the period of the automatic cleaning process.

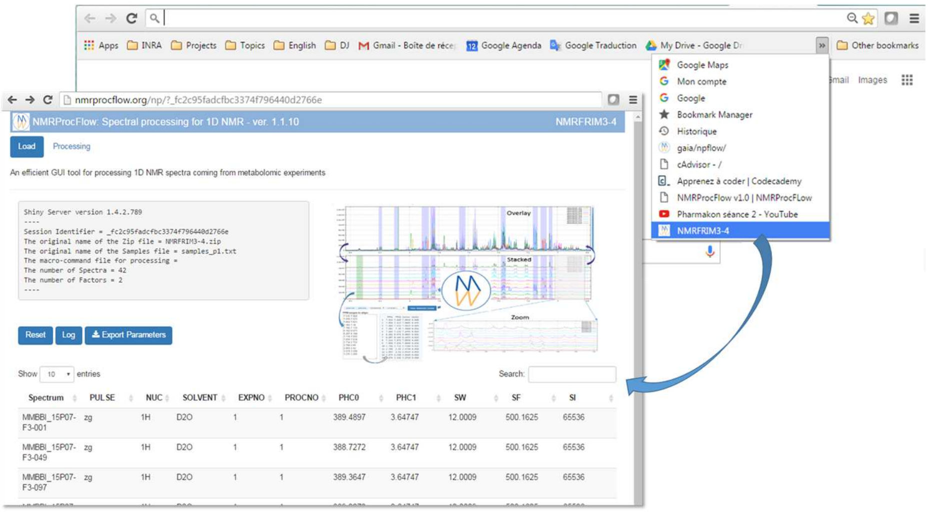

(C) Copyright INRA UMR 1332 BFP - MetaboHub 2016

Replay the same processing workflow

NMRProcFlow does not manage sessions in a medium or long term period.

- An automatic cleaning process has been implemented to periodically purge the working sessions with no activities over the past few days

The choice was to make a processing tool on the fly

- No need for large disk space and no need to backup

NMRProcFlow allows users to save on their own space a minimal set of small files ...

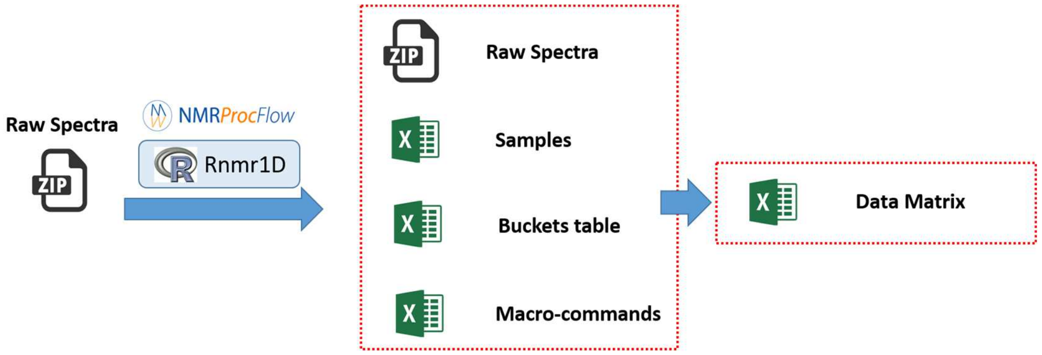

... in order to recover / replay their session

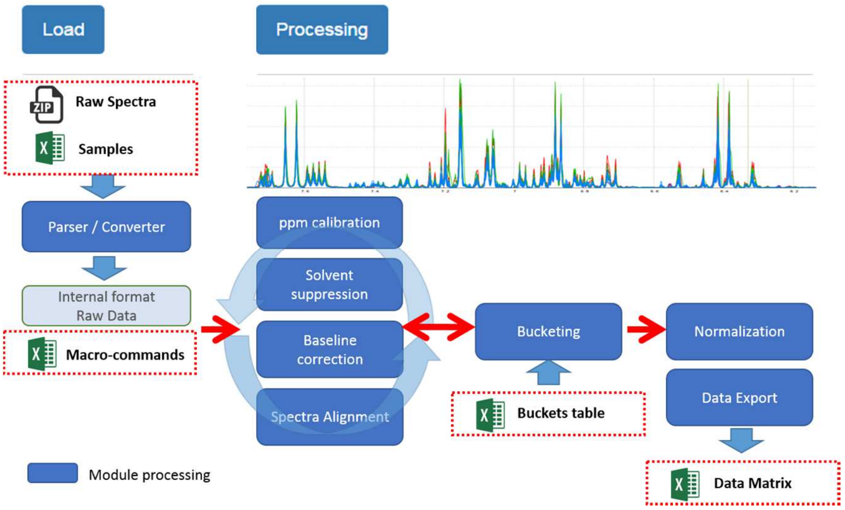

(C) Copyright INRA UMR 1332 BFP - MetaboHub 2016

How to regenerate a session with the same treatment?

1) Before existing your session, just export a file of macro-commands

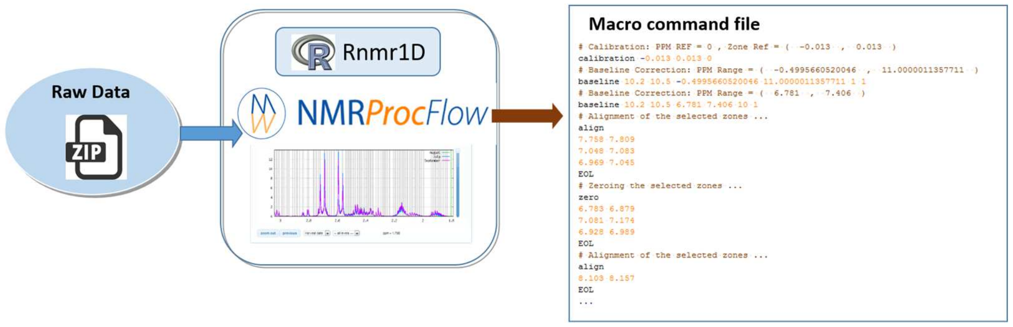

To save the processing commands in order to replay them later on the same or similar NMR spectra, just click on the CMD button

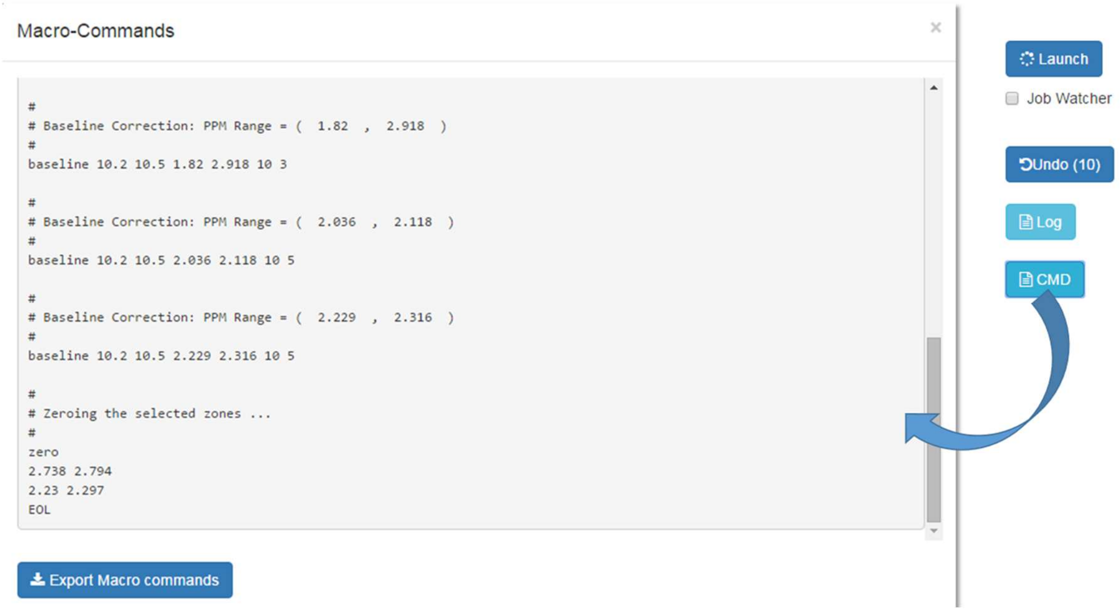

As shown in the figure below, all processing commands previously launched are logged into a macro-command file which can be saved on your local disk.

(C) Copyright INRA UMR 1332 BFP - MetaboHub 2016

2) Replay the same processing workflow ... few months later

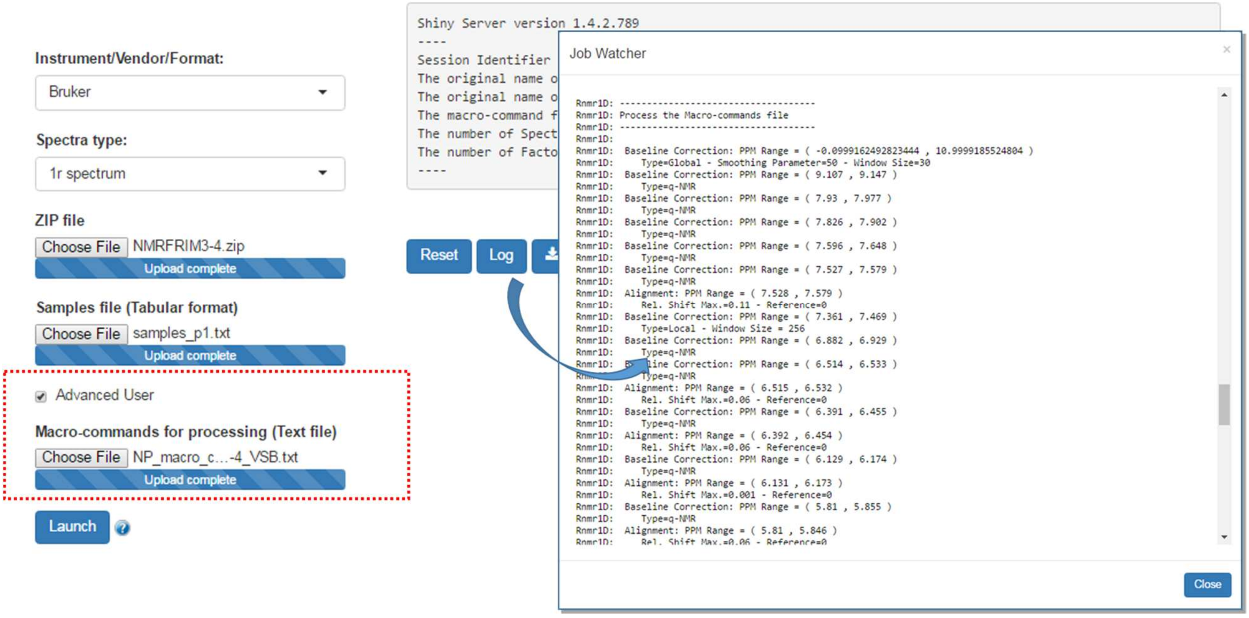

An option allows advanced users to submit a file of macro-commands along with the ZIP file of the raw spectra so that we can process the 1D NMR spectra in the same way as we proceeded them in a previous work session.

(C) Copyright INRA UMR 1332 BFP - MetaboHub 2016

Batch mode execution

NMRProcFlow allows experts to build their own spectra processing workflows, in order to become models applicable to similar NMR spectra sets, i.e. stated as use-cases.

In the Replay the same processing workflow section, we have seen how to export a macro-commands file in order to be replayed in the NMRProcFlow GUI, i.e. in interactive mode. Now the idea as depicted in the figure below, is to consider the macro-commands file as a processing model that can be applied on other subsets and replayed in batch mode.

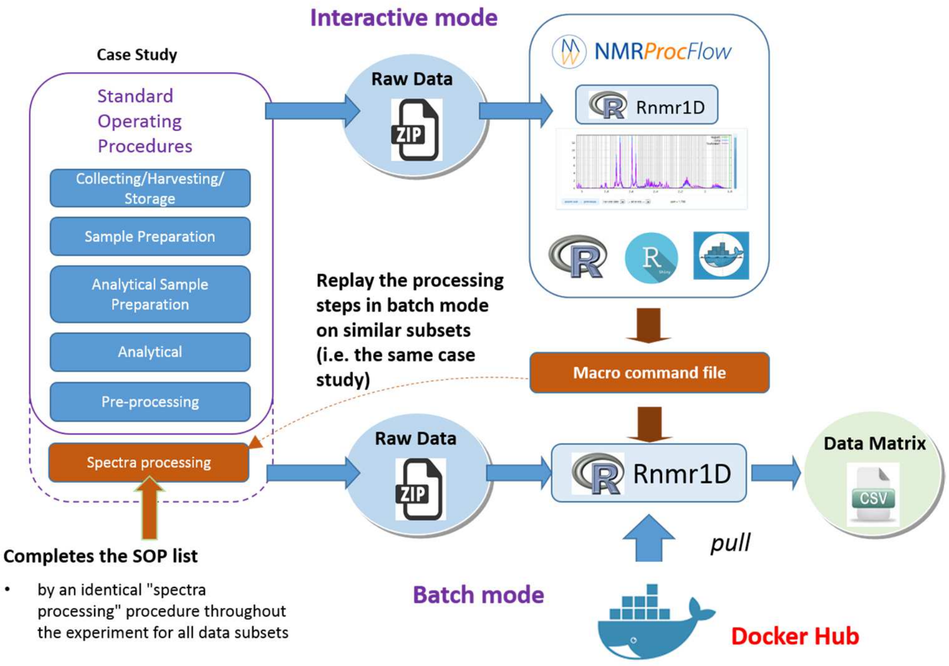

By extension, we consider the implementation of NMR spectra processing workflows executed in batch mode as relevant provided that we want to process in this way very well-mastered and very reproducible use cases, i.e. by applying the same Standard Operating Procedures (SOP).

- **Interactive mode**: A subset of NMR spectra is firstly processed in interactive mode using NMRProcFlow having a NMR spectra viewer to allow the expert eye to disentangle the intertwined peaks, in order to build a well-suited workflow. This mode could be also named the 'expert mode'.
- **Batch mode**: Then, other subsets that could be regarded as either similar or being included in the same case study, can be processed in batch mode (executed using the "rnmr1d" docker image, got from DockerHub)

Futur Work: NMRProcFlow combined with the computing power of the workflow4metabolomics infrastructure. See the presentation (http://nmrprocflow.org/themes/pdf/NMRProcFlow_W4M.pdf)

(C) Copyright INRA UMR 1332 BFP - MetaboHub 2016

How to proceed in Batch mode ?

Get the docker image

- Requirements: a recent Linux OS that support Docker (see https://www.docker.com/)
- Pull the docker image from DockerHub (may take a while depending on your network speed and the traffic)

```
$ sudo docker pull nmrprocflow/rnmr1d
```

Usage

```
$ sudo docker run -it --rm rnmr1d -h
Rnmr1D - Command Line Interface (CLI) of the NMR spectra processing module (R package '
Rnmr1D')

Usage:
  Rnmr1D [options]

Options:
  -h, --help            Show this screen.
  -d, --debug           Show more information
  -z, --zip <file>      the full path name of the ZIP file (raw.zip)
  -s, --samples <file>  the full path name of the Sample file (tabular format)
  -p, --proccmd <file>  the full path name of the Macro-commands file for processing (te
xt format)
  -b, --bucfile <file>  the full path name of the file of bucket's zones (tabular format
)
  -n, --outnorm <TYPE>  Normalization method. Possible values are: none, CSN, PQN [defau
lt: none]
  -c, --cpu <n>         the number of cores [default: 4]
  -o, --outdir <path>   the full path name of the directory to output the resulting file
s
  -l, --logfile <file>  the full path name of the LOG file [default: stderr]
```

Example

- From the "A full data set as example" section, download files in the 'input files' list, namely: '**NMRFRIM3-4.zip**', '**samples_p1.txt**', '**buckets_FRIM3-4.txt**' and '**NP_macro_cmd_NMRFRIM3-4.txt**', the whole saved under a directory, called for example '**exampledir/input**'.
- Run the docker image in order to replay the macro-command file on the NMR spectra dataset, and put the results under the '**exampledir/output**' directory.

It is important to note that in the command shown below, the '**exampledir**' directory is mounted as a volume to "**/data**" within the docker container. It means that the '**exampledir**' directory is seen by the docker container as "**/data**" in its own filesystem. This is why we specify "**/data**" as the root of all input/output files in the arguments because all commands will be internally executed within the docker container.

(C) Copyright INRA UMR 1332 BFP - MetaboHub 2016

```
sudo docker run -i --rm -v $PWD/exampledir:/data nmrprocflow/rnmr1d \
   -z /data/input/NMRFRIM3-4.zip \
   -s /data/input/samples_p1.txt \
   -p /data/input/NP_macro_cmd_NMRFRIM3-4.txt \
   -b /data/input/buckets_FRIM3-4.txt \
   -n none \
   -c 4 \
   -o /data/output
```

- In the standard output (stdout), you should see something like below:

```
Loading required package: methods
Loading required package: foreach
Loading required package: iterators
Loading required package: parallel
Rnmr1D:  Unzip the ZIP file ...
Rnmr1D:  --- READING and CONVERTING ---
[2/42]: MMBBI_15P07-F3-049

....

[41/42]: MMBBI_15P07-F4-061
Rnmr1D:  Generate the final matrix of spectra...
Rnmr1D:  Write the spec.pack file ...
Rnmr1D:  Write the list_pars.csv file ...
Rnmr1D: ------------------------------------
Rnmr1D: Process the Macro-commands file
Rnmr1D: ------------------------------------
Rnmr1D:
Rnmr1D:  Baseline Correction: PPM Range = ( -0.0999162492823444 , 10.9999185524804 )
Rnmr1D:     Type=Global - Smoothing Parameter=50 - Window Size=30
Rnmr1D:  Baseline Correction: PPM Range = ( 9.107 , 9.147 )
Rnmr1D:     Type=q-NMR
Rnmr1D:  Baseline Correction: PPM Range = ( 7.93 , 7.977 )
Rnmr1D:     Type=q-NMR
Rnmr1D:  Baseline Correction: PPM Range = ( 7.826 , 7.902 )
Rnmr1D:     Type=q-NMR
Rnmr1D:  Baseline Correction: PPM Range = ( 7.596 , 7.648 )
Rnmr1D:     Type=q-NMR
Rnmr1D:  Baseline Correction: PPM Range = ( 7.527 , 7.579 )
Rnmr1D:     Type=q-NMR
Rnmr1D:  Alignment: PPM Range = ( 7.528 , 7.579 )
Rnmr1D:     Rel. Shift Max.=0.11 - Reference=0

...

Rnmr1D:  Baseline Correction: PPM Range = ( 1.012 , 1.065 )
Rnmr1D:     Type=q-NMR
Rnmr1D:  Write the spec.pack file ...
Rnmr1D:
Rnmr1D: ------------------------------------
Rnmr1D: Process the file of buckets
Rnmr1D: ------------------------------------
Rnmr1D:
Rnmr1D: NB Buckets = 38
Rnmr1D:
Rnmr1D:
   user  system elapsed
 14.094   3.323   8.649
```


(C) Copyright INRA UMR 1332 BFP - MetaboHub 2016

- Visualize / explore the processed NMR spectra using the NMR viewer
- First, launch the NMR viewer by creating the corresponding docker container

```
sudo docker run -d -v $PWD/exampledir:/opt/data -p 8080:80 nmrprocflow/nmrview nmrview
```

- Then, in your favorite web browser, go to the URL:

```
http://<your_hostname>:8080/nv/view/output
```


(C) Copyright INRA UMR 1332 BFP - MetaboHub 2016

Examples in action

Example 1: Spectra alignment performed on a ppm window

This example is based on 1H NMR spectra from leaves of grapevine (D2O Solvent, NOESY Pulse sequence, pH 6, NMR 500MHz)

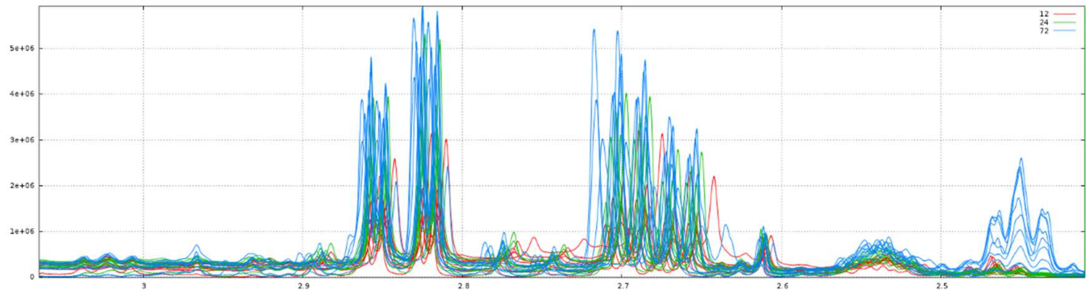

Global Baseline correction ...

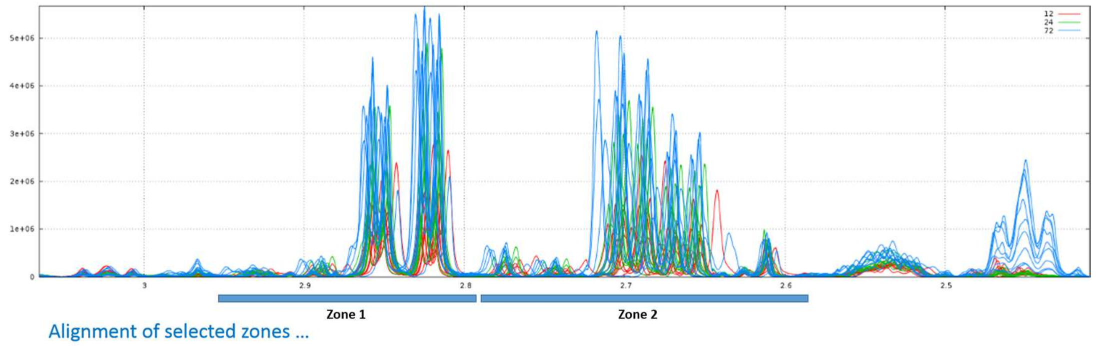

Alignment of selected zones ...

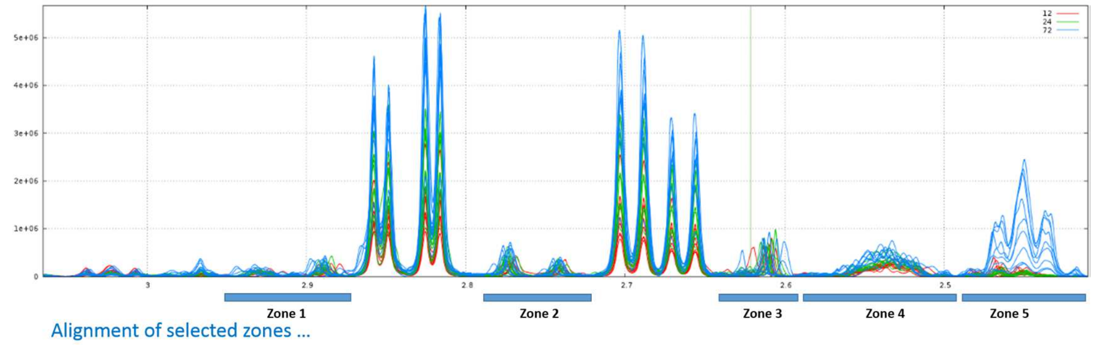

Alignment of selected zones ...

(C) Copyright INRA UMR 1332 BFP - MetaboHub 2016

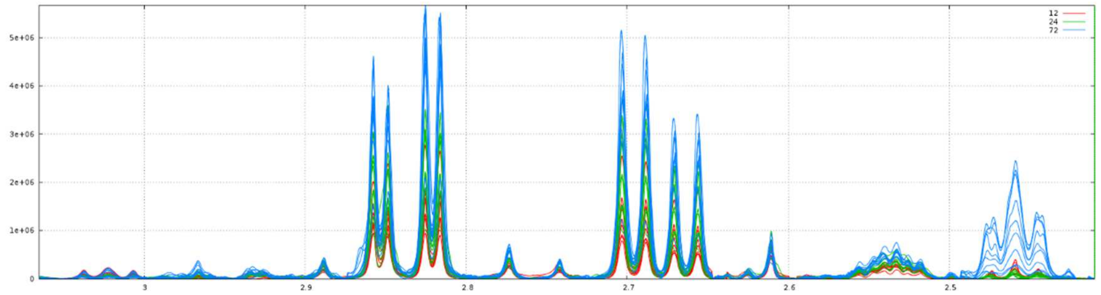

It seems fine

Example 2: A complete processing performed on a ppm window

Here is a subset of NMR spectra (Brain of Mice, D2O Solvent, NOESY Pulse sequence, pH 4, NMR 600MHz) within a ppm range

Example coming from MetaToul Platform: Cabaton, N. et al (2013).Effects of low doses of bisphenol A on the metabolome of perinatally exposed CD-1 mice. Environmental Health Perspectives, 121 (5), 586-593. DOI : 10.1289/ehp.1205588

For more details, see W4M - NMR Mus musculus dataset (http://workflow4metabolomics.org/node/48)

Before applying the several processing steps

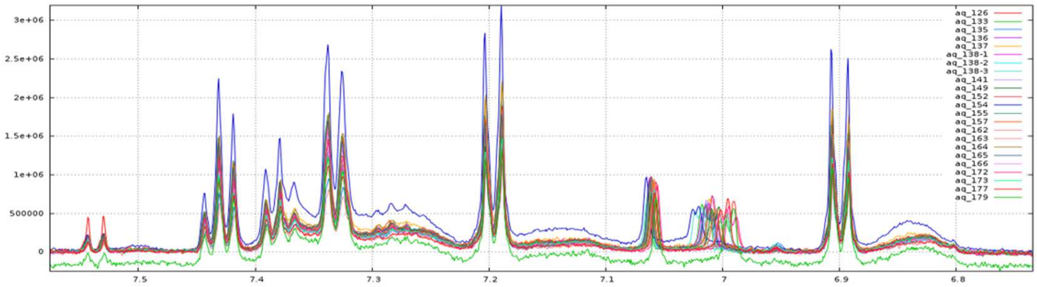

After treatments

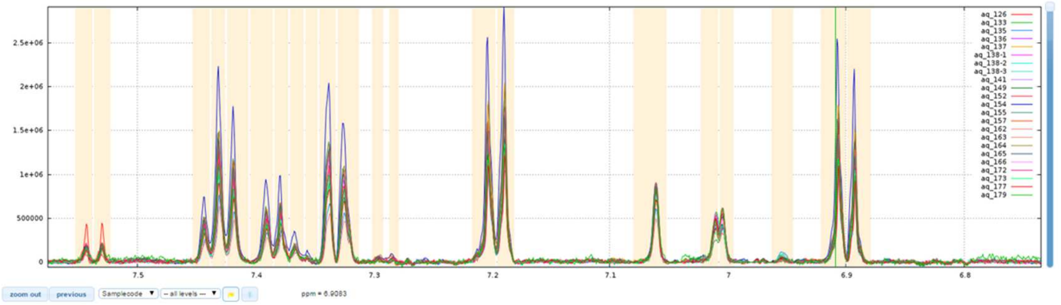

(C) Copyright INRA UMR 1332 BFP - MetaboHub 2016

Among the performed treatments:

- there was an overall correction of the base line, then several local corrections to eliminate the effect of the presence of proteins within the analytical samples;
- thereafter, a realignment was carried out on the two areas between 6.98 and 7.08 ppm;
- finally a bucketing called "intelligent binning" was performed

Example 3: Another complete processing performed on a ppm window

Here is a subset of NMR spectra (48 1H-NMR spectra of Tomato pericarp extracts, D2O Solvent, single Pulse sequence, pH 6, NMR 500MHz) within a ppm range. To download the complete dataset, see "A full data set as example". (http://nmrprocflow.org/ex1)

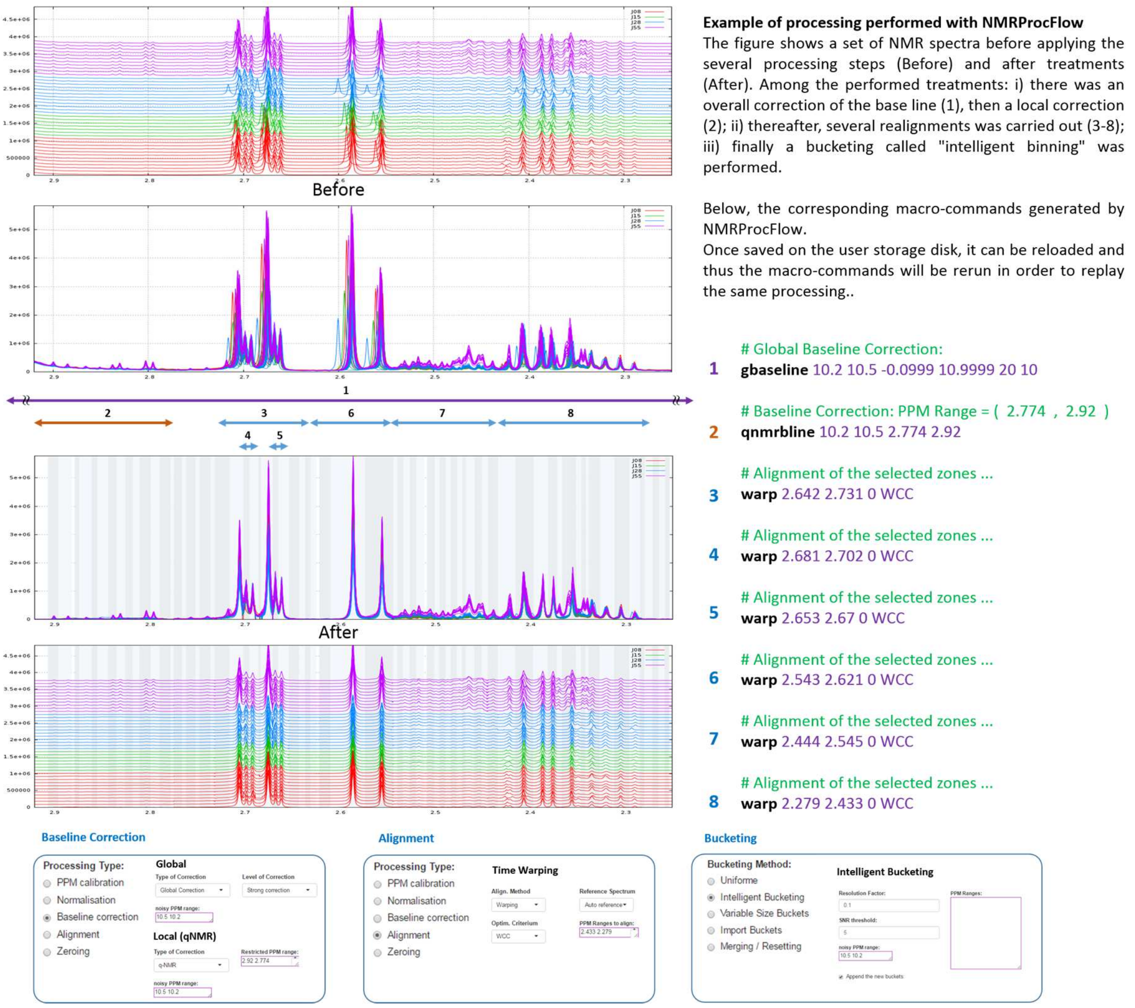

(C) Copyright INRA UMR 1332 BFP - MetaboHub 2016

Download

There are two ways to install NMRProcFlow locally, depending on the type of installation.

Desktop PC

If you want to install NMRProcFlow on your desktop PC , laptop or netbook, then the best solution is to use a virtualization platform like Oracle VirtualBox

Server

If the installation is to be done on a Linux server, then undoubtedly, the best solution is to use Docker containers.

(C) Copyright INRA UMR 1332 BFP - MetaboHub 2016

Virtual Appliance

We provided virtual appliance so that users can install and use NMRProcFlow on their own computer, enabling them to process sensitive and confidential data, and also with a larger data size (the size of the ZIP is limited to 200 MB on the online version).

- **Installation guides**:
    - For VirtualBox
        - http://nmrprocflowthemes/pdf/NMRProcFlow_install_VBox.pdf
    - For VMware Player
        - http://nmrprocflowthemes/pdf/NMRProcFlow_install_VMware.pdf

- **VirtualBox**:
    - Tested and valided with Oracle VM VirtualBox 5.1.10 under Windows 7 Pro and Windows 10 Pro
    - You can download the Oracle VM VirtualBox software along with the Oracle VM VirtualBox Extension Pack (https://www.virtualbox.org/wiki/Downloads)
    - Download the virtual disk (VDI) - (1.2 Go):
        - http://nmrprocflow.org/themes/ova/npflow_vbox_vdi_x64.zip

- **VMware Player** *(Warning: it will be no longer supported in a near futur)*:
    - Tested and valided with VMware (R) Player 6.0.7 under Windows 7 Pro
    - You can download the VMware Player software https://pcappsstore.blogspot.fr/2015/12/vmware-player-for-windows.html
    - Download the corresponding OVA file (707 Mo)
        - http://nmrprocflow.org/themes/ova/npflow_vmware_x64.ova

(C) Copyright INRA UMR 1332 BFP - MetaboHub 2016

- **Note for Windows 10 Pro**:
  - Disable the Hyper-V feature otherwise the virtual machine platforms do not work in 64-bit mode
  - See http://www.poweronplatforms.com/enable-disable-hyper-v-windows-10-8/
  - Use only VirtualBox from version 5.1.10 or higher

- **Note for Mac OS X 10.x**:
  - We have proceed no installation tests on Mac OS X 10.x but severals users have successful installed NMRProcFlow using VirtualBox 5.1.10. Many online helps exist, and we have selected the following:
  - Installing VirtualBox and extension packs:
    - https://www.virtualbox.org/manual/ch01.html#intro-installing
  - How to Setup Nat Network and Port Forwarding Virtualbox:
    - https://www.youtube.com/watch?v=nxFfaXVcEMc

(C) Copyright INRA UMR 1332 BFP - MetaboHub 2016

Docker Images

What is Docker?

Docker is a tool designed to make it easier to create, deploy, and run applications by using containers. Containers allow a developer to package up an application with all of the parts it needs, such as libraries and other dependencies, and ship it all out as one package. By doing so, thanks to the container, the developer can rest assured that the application will run on any other Linux machine regardless of any customized settings that machine might have that could differ from the machine used for writing and testing the code.

Installation

Requirements: a recent OS that support Docker

Get the docker images

- Pull the two docker images from DockerHub (may take a while depending on your network speed and the traffic)

```
$ sudo docker pull nmrprocflow/nmrview
$ sudo docker pull nmrprocflow/nmrproc
```

Create minimal configuration files:

- **npflow.conf**

```
# The URL root of the PROXY if applicable
PROXY_URL_ROOT=

# Duration (in days) of validity of a session
# before its destruction (counted from the last change)
PURGESESSIONS=2

# Max ZIP size (Mo)
MAXZIPSIZE=400

# NB CORES (0 means Auto)
CORES=0

# User connexion management
# 0 : no connexion management
# 1 : connexion management based on the /opt/data/conf/userlist file
#     Its structure is one user per line and each line following the format:
#        login;LastName;FirstName;Country;Institution;Email;Password
```


(C) Copyright INRA UMR 1332 BFP - MetaboHub 2016

```
#     a minimal set of this 'userlist' file could be: npflow;;;;;;nppass
USRCONMGR=0
```

- **nview.conf**

```
# The URL root of the PROXY if applicable
PROXY_URL_ROOT=
```

Create a shell script file (Linux):

- **npflow.sh**

```
#!/bin/bash
MYDIR=`dirname $0` && [ ! `echo "$0" | grep '^\/'` ] && MYDIR=`pwd`/$MYDIR

DATADIR=/opt/data

# nmrview Container
VIEW_IMAGE=nmrprocflow/nmrview
VIEW_CONTAINER=nmrview
VIEW_CONF=$MYDIR/nview.conf

# nmrspec Container
SPEC_PORT=8080
SPEC_IMAGE=nmrprocflow/nmrspec
SPEC_CONTAINER=nmrspec
SPEC_CONF=$MYDIR/npflow.conf

CMD=$1

# If you use a named volume, (assumes that your docker version >= 1.9)
# - First you have to create the /opt/data volume
# sudo docker create -v /opt/data --name npflow_data_volume ubuntu
# - Second, uncomment the line below, and
#   comment the line with 'VOLS' specified further with a local directory .
#VOLS="--volumes-from npflow_data_volume"

# If you use a local directory, first you have to create the /opt/data directory
VOLS="-v $DATADIR:/opt/data"

usage() { echo "usage: sh $0 start|stop|ps|restart|logs|update";  exit 1; }

case "$CMD" in
   start)
        # run NMRviewer
        sudo docker run -d --env-file $VIEW_CONF $VOLS --name $VIEW_CONTAINER $VI
EW_IMAGE

        # run NMRProcFlow
        sudo docker run -d --env-file $SPEC_CONF $VOLS  -p $SPEC_PORT:80 \
                               --link $VIEW_CONTAINER:nvapp --name $SPEC_CONTAINE
R $SPEC_IMAGE
```


(C) Copyright INRA UMR 1332 BFP - MetaboHub 2016

```
        # show Logs
        sudo docker logs $VIEW_CONTAINER
        sudo docker logs $SPEC_CONTAINER
        ;;
   stop)
        sudo docker rm -f $SPEC_CONTAINER $VIEW_CONTAINER
        ;;
   restart)
        ( sh $0 stop; sh $0 start)
        ;;
   logs)
        sudo docker logs $VIEW_CONTAINER
        sudo docker logs $SPEC_CONTAINER
        ;;
   ps)
        sudo docker ps | head -1
        sudo docker ps | grep "nmrprocflow/"
        ;;
   update)
        sudo docker pull $VIEW_IMAGE
        sudo docker pull $SPEC_IMAGE
        ;;
   *) usage
      exit 2
esac
```

Start the application (Linux)

```
sh ./npflow.sh start
```

Then, in your favorite web browser, check on :

```
http://<your_vm_host>:8080/npflow/
```

Stop the application (Linux)

```
sh ./npflow.sh stop
```

Status of the application (Linux)

```
sh ./npflow.sh ps
```


(C) Copyright INRA UMR 1332 BFP - MetaboHub 2016

View logs of the application (Linux)

```
sh ./npflow.sh logs
```

NGINX configuration (Linux)

For avanced users: In case you would like to use a proxy server, the better is to install and set NGINX, an HTTP and reverse proxy server.

In the /etc/nginx/conf.d/my-site.conf, you should add three 'location' sections as shown below:

```
server {
    listen 80 default;
    server_name $host;

    ...

    location /nv/ {
        proxy_pass http://localhost:8080/nv/;
    }

    location /npwatch/ {
        proxy_pass http://localhost:8080/npwatch/;
    }

    location /np/ {
        proxy_pass http://localhost:8080/npflow/;
        proxy_redirect http://localhost:8080/npflow/ $scheme://$host/npflow/;
        proxy_http_version 1.1;
        proxy_set_header Upgrade $http_upgrade;
        proxy_set_header Connection "upgrade";
        proxy_set_header Host $host;
    }

    ...

}
```


(C) Copyright INRA UMR 1332 BFP - MetaboHub 2016
